# Curcumin, thymoquinone, and 3, 3′-diindolylmethane combinations attenuate lung and liver cancers progression

**DOI:** 10.3389/fphar.2022.936996

**Published:** 2022-06-29

**Authors:** Amna A. Saddiq, Ali H. El-Far, Shymaa Abdullah Mohamed Abdullah, Kavitha Godugu, Omar A. Almaghrabi, Shaker A. Mousa

**Affiliations:** ^1^ College of Sciences, Department of Biology, University of Jeddah, Jeddah, Saudi Arabia; ^2^ Department of Biochemistry, Faculty of Veterinary Medicine, Damanhour University, Damanhour, Egypt; ^3^ Molecular Biology Unit, Medical Technology Center and Applied Medical Chemistry Department, Medical Research Institute, Alexandria University, Alexandria, Egypt; ^4^ Pharmaceutical Research Institute, Albany College of Pharmacy and Health Sciences, Rensselaer, NY, United States; ^5^ College of Sciences, Department of Biology, University of Jeddah, Jeddah, Saudi Arabia; ^6^ Pharmaceutical Research Institute, Albany College of Pharmacy and Health Sciences, Rensselaer, NY, United States

**Keywords:** curcumin, thymoquinone, 3,3′-diindolylmethane, combination, apoptosis, cell migration, tumor growth, tumor angiogenesis

## Abstract

Cancer can develop due to abnormal cell proliferation in any body’s cells, so there are over a hundred different types of cancer, each with its distinct behavior and response to treatment. Therefore, many studies have been conducted to slow cancer progression and find effective and safe therapies. Nutraceuticals have great attention for their anticancer potential. Therefore, the current study was conducted to investigate the anticancer effects of curcumin (Cur), thymoquinone (TQ), and 3, 3′-diindolylmethane (DIM) combinations on lung (A549) and liver (HepG2) cancer cell lines’ progression. Results showed that triple (Cur + TQ + DIM) and double (Cur + TQ, Cur + DIM, and TQ + DIM) combinations of Cur, TQ, and DIM significantly increased apoptosis with elevation of caspase-3 protein levels. Also, these combinations exhibited significantly decreased cell proliferation, migration, colony formation activities, phosphatidylinositol 3-kinase (PI3K), and protein kinase B (AKT) protein levels with S phase reduction. Triple and double combinations of Cur, TQ, and DIM hindered tumor weight and angiogenesis of A549 and HepG2 implants in the chorioallantoic membrane model. Interestingly, Cur, TQ, and DIM combinations are considered promising for suppressing cancer progression *via* inhibiting tumor angiogenesis. Further preclinical and clinical investigations are warranted.

## Introduction

Cancer ranks as a leading cause of death worldwide (Bray et al., 2021). The International Agency for Research on Cancer estimated that lung cancer remained the leading cause of cancer death, with an estimated 1.8 million deaths (18%), followed by colorectal (9.4%), liver (8.3%), stomach (7.7%), and female breast (6.9%) cancers worldwide (Sung et al., 2021). Thai et al. (2021) reported that lung cancer was estimated at 2 million new cases and 1.76 million deaths per year worldwide. In the same context, liver cancer is one of the leading causes worldwide deaths. Hepatitis B virus, hepatitis C virus, alcohol consumption, and nonalcoholic fatty liver disease are the primary causes of liver cancer (Lin et al., 2020).

**FIGURE 1:**
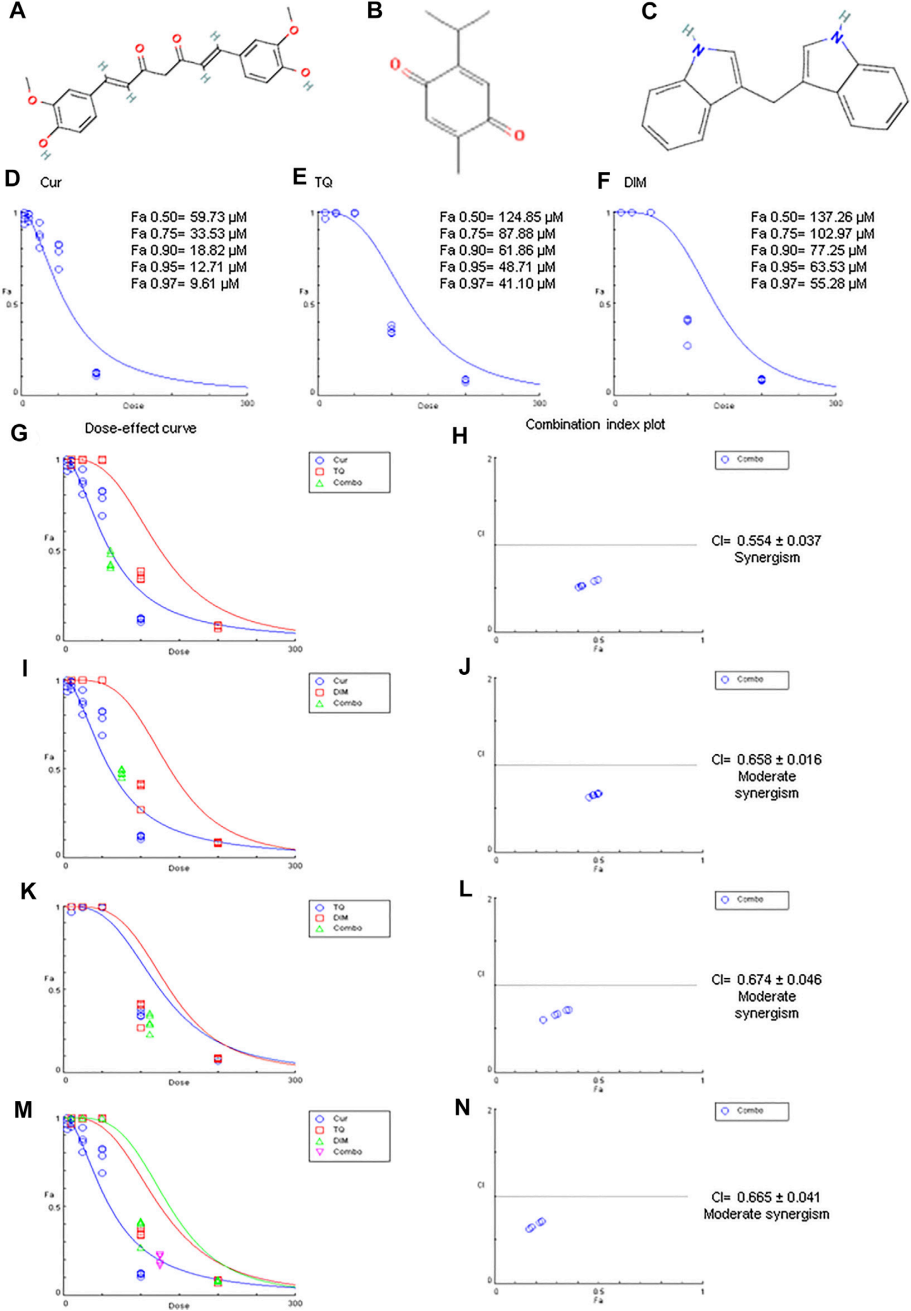
Chemical structures, Fa values, and combination index of curcumin (Cur), thymoquinone (TQ), and 3,3′-diindolylmethane (DIM) against A549. **(A)** Chemical structure of Cur. **(B)** Chemical structure of TQ. **(C)** Chemical structure of DIM. **(D)** Fa values of Cur against A549. **(E)** Fa values of TQ against A549. **(F)** Fa values of TQ against A549. **(G)** Dose-effect curve of Fa 95 of Cur (12.71 µM) and TQ (48.71 µM). **(H)** Combination index plot of Fa 95 of Cur and TQ. **(I)** Dose-effect curve of Fa 95 of Cur (12.71 µM) and DIM (63.53 µM). **(J)** Combination index plot of Fa 95 of Cur and DIM. **(K)** Dose-effect curve of Fa 95 of TQ (48.71 µM) and DIM (63.53 µM). **(L)** Combination index plot of Fa 95 of TQ and DIM. **(M)** Dose-effect curve of Fa 95 of Cur (12.71 µM), TQ (48.71 µM), and DIM (63.53 µM). **(N)** Combination index plot of Fa 95 of Cur, TQ, and DIM. The CI values represent the mean of four experiments. CI > 1.3: antagonism; CI (1.1–1.3): moderate antagonism; CI (0.9–1.1): additive effect; CI (0.8–0.9): slight synergism; CI (0.6–0.8): moderate synergism; CI (0.4–0.6): synergism; CI (0.2–0.4): strong synergism.

A healthy diet that reduces cancer incidence has led many researchers to focus on natural products to prevent cancer (den Hollander et al., 2013). Nutraceuticals fight cancer by inhibiting proliferation, migration, metastasis, angiogenesis, cell cycle arrest, and increasing cancer cells’ sensitivity to radiotherapy and chemotherapy (Li et al., 2017). The American Institute for Cancer Research and the World Cancer Research Fund stated that 30%–40% of all cancers could be prevented by appropriate diets and physical activity (Glade, 1999). Also, natural products in medicinal plants have been used to treat human diseases for thousands of years in Asia (Koehn and Carter, 2005). Curcumin (Cur) (Figure 1A), the active ingredient of *Curcuma longa* L. (Kew Medicinal Plant Names Services (MPNS) - validated), is the most studied compound described as a potential anticancer agent due to its multi-targeted signaling/molecular pathways (Sharma and Martins, 2020; Shah et al., 2021). Also, Cur targeted phosphatidylinositol 3-kinase (PI3K)/protein kinase B (AKT), Janus kinase/signal transducer and activator of transcription, and nuclear factor kappa B (NF-κB) pathways (Wong et al., 2021) in almost most cancer forms (El-Far et al., 2020a; Joshi et al., 2021). The taxonomic hierarchy of *Curcuma longa* L. is shown in Table 1 (USDA Plants Database, 2022a).

**TABLE 1:** Taxonomic hierarchy of *Curcuma longa* L. and *Nigella sativa* L.

	*Curcuma longa* L	*Nigella sativa* L
Kingdom	*Plantae* - Plants	*Plantae* - Plants
Subkingdom	*Tracheobionta* - Vascular plants	*Tracheobionta* - Vascular plants
Superdivision	*Spermatophyta* - Seed plants	*Spermatophyta* - Seed plants
Division	*Magnoliophyta* - Flowering plants	*Magnoliophyta* - Flowering plants
Class	*Liliopsida* - Monocotyledons	*Magnoliopsida* - Dicotyledons
Subclass	Zingiberidae	Magnoliidae
Order	*Zingiberales*	*Ranunculales*
Family	Zingiberaceae - Ginger family	Ranunculaceae - Buttercup family
Genus	*Curcuma* L. - curcuma	*Nigella* L. - nigella
Species	*Curcuma longa* L. - common turmeric	*Nigella sativa* L.—black cumin

Thymoquinone (TQ) (Figure 1B), the bioactive constituent of *Nigella sativa* L. (MPNS - validated) seeds, is a well-known natural bioactive compound used for the management of several cancer types (El-Far, 2015; El-Far AH. et al., 2018, 2020b, 2020a, El-Far et al., 2021 A. H.). TQ induced their anticancer potential by preventing inflammation and oxidative stress, inhibiting angiogenesis and metastasis, and inducing apoptosis of cancer cells (Alhmied et al., 2021). In the same context, 3,3′-diindolylmethane (DIM) (Figure 1C) is present in cruciferous vegetables, including broccoli, Brussels sprouts, cabbage, cauliflower, kale, turnips, collard greens, kohlrabi, and mustard rutabaga has antiproliferative and anticancer activities in various cancer cells (Zhang et al., 2014; Munakarmi et al., 2021). The taxonomic hierarchy of *Nigella sativa* L. is shown in Table 1 (USDA Plants Database, 2022b).

Cur, TQ, and DIM were incorporated in clinical trials (https://clinicaltrials.gov/) for cancer therapy, as stated in Supplementary Tables S1–S3, respectively. We have not found a study about the combination of Cur, TQ, and DIM exploring their role in cancer repression. Therefore, in the current study, we investigated the combinatory effect of Cur, TQ, and DIM on lung (A549) and liver (HepG2) cancer cell lines’ progression.

## Materials and methods

### Cell lines

Lung (A549) and liver (HepG2) cancer cell lines were purchased from ATCC (Manassas, VA, United States). A549 and HepG2 cells were grown in low-glucose Dulbecco’s Modified Eagle Medium supplemented with 10% fetal bovine serum and 1% penicillin/streptomycin solution.

### MTT [3-(4,5-dimethylthiazol-2-yl)-2,5-diphenyltetrazolium bromide] and combination index assays

The half-maximal inhibitory concentration (IC_50_) of Cur, TQ, and DIM was determined by seeding A549 or HepG2 cells in 24-well plates (3 × 10^4^ per well in 1.5 ml) and incubated for 48 h at 37°C in a 5% CO_2_ incubator. Cells were treated with 1 ml of Cur (0, 5, 10, 25, 50, or 100 μM dissolved in DMSO), TQ (0, 10, 25, 50, 100, or 200 μM dissolved in DMSO), and DIM (0, 10, 25, 50, 100, or 200 μM dissolved in DMSO) and incubated for 24 h, then treated with MTT reagent (1.25 mg/ml) and incubated for 2 h. The resulting formazan crystals were dissolved in 1 ml DMSO, and the optical density was determined using a microplate reader at 570 nm (El-Far et al., 2020a). DMSO was used in a concentration of 0.1% of medium.

The data of the MTT assay was analyzed by CompuSyn software (https://www.combosyn.com/) to determine the IC_50_ of Cur and TQ against A549 and HepG2 cells. These experiments were repeated three times (Chou, 2011). The combination index of Cur, TQ, and DIM were determined by CompuSyn software at Fa 95 against A549 and HepG2 cells, shown in Table 2. The Fa is the fraction affected, so; the Fa 95 value means the concentration of the drug at which 5% of cells are affected by this drug.

**TABLE 2:** Summarizes the data of Fa values and the concentrations of curcumin (Cur), thymoquinone (TQ), 3,3′-diindolylmethane (DIM) and their combinations that used in all assays.

Assays	Drugs	A549	HepG2
Fa values	Cur (μM)	Fa 0.50 = 59.73	Fa 0.50 = 101.80
		Fa 0.75 = 33.53	Fa 0.75 = 56.58
		Fa 0.90 = 18.82	Fa 0.90 = 31.45
		Fa 0.95 = 12.71	Fa 0.95 = 21.10
		Fa 0.97 = 9.61	Fa 0.97 = 15.88
	TQ (μM)	Fa 0.50 = 124.85	Fa 0.50 = 105.78
		Fa 0.75 = 87.88	Fa 0.75 = 61.71
		Fa 0.90 = 61.86	Fa 0.90 = 36.00
		Fa 0.95 = 48.71	Fa 0.95 = 24.95
		Fa 0.97 = 41.10	Fa 0.97 = 19.22
	DIM (μM)	Fa 0.50 = 137.26	Fa 0.50 = 163.18
		Fa 0.75 = 102.97	Fa 0.75 = 109.75
		Fa 0.90 = 77.25	Fa 0.90 = 73.82
		Fa 0.95 = 63.53	Fa 0.95 = 56.36
		Fa 0.97 = 55.28	Fa 0.97 = 46.52
Combination index	Cur (μM)	Fa 95 = 12.71	Fa 95 = 21.10
	TQ (μM)	Fa 95 = 48.71	Fa 95 = 24.95
	DIM (μM)	Fa 95 = 63.53	Fa 95 = 56.36
Cytotoxicity percentages	Cur (μM)	Fa 95 = 12.71	Fa 95 = 21.10
		0.75 (Fa 95) = 9.53	0.75 (Fa 95) = 15.83
		0.50 (Fa 95) = 6.36	0.50 (Fa 95) = 10.55
		0.25 (Fa 95) = 3.18	0.25 (Fa 95) = 5.28
	TQ (μM)	Fa 95 = 48.71	Fa 95 = 24.95
		0.75 (Fa 95) = 36.53	0.75 (Fa 95) = 18.71
		0.50 (Fa 95) = 24.36	0.50 (Fa 95) = 12.48
		0.25 (Fa 95) = 12.18	0.25 (Fa 95) = 6.24
	DIM (μM)	Fa 95 = 63.53	Fa 95 = 56.36
		0.75 (Fa 95) = 47.65	0.75 (Fa 95) = 42.27
		0.50 (Fa 95) = 31.77	0.50 (Fa 95) = 28.18
		0.25 (Fa 95) = 15.88	0.25 (Fa 95) = 14.09
Annexin-V positive cell percentages	Cur (μM)	0.75 (Fa 95) = 9.53	0.75 (Fa 95) = 15.83
	TQ (μM)	0.75 (Fa 95) = 36.53	0.75 (Fa 95) = 18.71
	DIM (μM)	0.75 (Fa 95) = 47.65	0.75 (Fa 95) = 42.27
Cell cycle analysis	Cur (μM)	0.75 (Fa 95) = 9.53	0.75 (Fa 95) = 15.83
	TQ (μM)	0.75 (Fa 95) = 36.53	0.75 (Fa 95) = 18.71
	DIM (μM)	0.75 (Fa 95) = 47.65	0.75 (Fa 95) = 42.27
Cell proliferation assay	Cur (μM)	0.75 (Fa 95) = 9.53	0.75 (Fa 95) = 15.83
	TQ (μM)	0.75 (Fa 95) = 36.53	0.75 (Fa 95) = 18.71
	DIM (μM)	0.75 (Fa 95) = 47.65	0.75 (Fa 95) = 42.27
Migration assay	Cur (μM)	0.75 (Fa 95) = 9.53	0.75 (Fa 95) = 15.83
	TQ (μM)	0.75 (Fa 95) = 36.53	0.75 (Fa 95) = 18.71
	DIM (μM)	0.75 (Fa 95) = 47.65	0.75 (Fa 95) = 42.27
Colony formation assay	Cur (μM)	Fa 95 = 12.71	Fa 95 = 21.10
		0.75 (Fa 95) = 9.53	0.75 (Fa 95) = 15.83
		0.50 (Fa 95) = 6.36	0.50 (Fa 95) = 10.55
		0.25 (Fa 95) = 3.18	0.25 (Fa 95) = 5.28
	TQ (μM)	Fa 95 = 48.71	Fa 95 = 24.95
		0.75 (Fa 95) = 36.53	0.75 (Fa 95) = 18.71
		0.50 (Fa 95) = 24.36	0.50 (Fa 95) = 12.48
		0.25 (Fa 95) = 12.18	0.25 (Fa 95) = 6.24
	DIM (μM)	Fa 95 = 63.53	Fa 95 = 56.36
		0.75 (Fa 95) = 47.65	0.75 (Fa 95) = 42.27
		0.50 (Fa 95) = 31.77	0.50 (Fa 95) = 28.18
		0.25 (Fa 95) = 15.88	0.25 (Fa 95) = 14.09
Enzyme-linked immunosorbent (ELISA) assay	Cur (μM)	Fa 95 = 12.71	Fa 95 = 21.10
		0.75 (Fa 95) = 9.53	0.75 (Fa 95) = 15.83
		0.50 (Fa 95) = 6.36	0.50 (Fa 95) = 10.55
		0.25 (Fa 95) = 3.18	0.25 (Fa 95) = 5.28
	TQ (μM)	Fa 95 = 48.71	Fa 95 = 24.95
		0.75 (Fa 95) = 36.53	0.75 (Fa 95) = 18.71
		0.50 (Fa 95) = 24.36	0.50 (Fa 95) = 12.48
		0.25 (Fa 95) = 12.18	0.25 (Fa 95) = 6.24
	DIM (μM)	Fa 95 = 63.53	Fa 95 = 56.36
		0.75 (Fa 95) = 47.65	0.75 (Fa 95) = 42.27
		0.50 (Fa 95) = 31.77	0.50 (Fa 95) = 28.18
		0.25 (Fa 95) = 15.88	0.25 (Fa 95) = 14.09
Chorioallantoic membrane (CAM) model	Cur (μg)	3.5	5.8
	TQ (μg)	6	3
	DIM (μg)	11.7	10.4

### Cytotoxicity assay

After determination of Fa 75, 90, 95, and 97 of Cur, TQ, and DIM using CompuSyn software against A549 and HepG2 cells, cells were seeded in 12-well plates (3 × 10^4^ per well in 1.5 ml) and incubated for 48 h at 37°C in a 5% CO_2_ incubator. Control, Cur, TQ, and DIM groups of A549 cells were treated with Fa 95, 0.75 (Fa 95), 0.50 (Fa 95), and 0.25 (Fa 95) in 1 ml of the medium. Similarly, control, Cur, TQ, and DIM groups of HepG2 cells were treated with Fa 95, 0.75 (Fa 95), 0.50 (Fa 95), and 0.25 (Fa 95) in 1 ml of the medium (El-Far et al., 2020a). After 24 h, cells were treated with MTT and visualized as described in the MTT assay. This experiment was repeated three times.

### Annexin-V assay

Apoptosis was analyzed by flow cytometry using annexin V-fluorescein isothiocyanate (annexin-FITC) and propidium iodide (PI) detection kit (BD Biosciences, San Jose, CA, United States). A549 and HepG2 cells were cultured in T25 flasks for 48 h. A549 cells were treated with 0.75 (Fa 95) of Cur, TQ, DIM, and their double (Cur + TQ, Cur + DIM, and TQ + DIM) and triple (Cur + TQ + DIM) combinations in 10 ml of medium for 24 h (Table 2). Also, HepG2 cells were treated with 0.75 (Fa 95) of Cur, TQ, and DIM and their double and triple combinations in 10 ml of medium for 24 h (Table 2). Cells were collected and centrifuged at 500 ×g for 5 min at room temperature after their trypsinization. The pellet was rinsed twice with phosphate-buffered saline (PBS) and then resuspended in a proper volume of binding buffer. After adding 10 μl of annexin V-FITC followed by gentle mixing, incubated for 15 min at room temperature in the dark and washed. The fluorescence intensity of FITC was carried on a FACSCalibur™ (Becton Dickinson) instrument using Cell Quest software (El-Far et al., 2020a; El-Far et al., 2021).

### Cell cycle analysis

Using a FACSCalibur™ flow cytometer (Becton Dickinson, San Jose, CA, United States) and Cell Quest software, the cell cycle status was analyzed in A549 and HepG2 cells that were cultured and treated with Cur, TQ, and DIM by the same method mentioned in the annexin-V assay.

### Cell proliferation assay

A549 or HepG2 cells were cultured in 12-well plates (3 × 10^4^ per well in 1.5 ml) and incubated for 48 h at 37°C in a 5% CO_2_ incubator. Cells were treated with 0.75 (Fa 95) of Cur, TQ, DIM, and their double and triple combinations in 1 ml for 24 h (Table 2). Cells were visualized and counted by an inverted light microscope (Primovert, Zeiss, Carl Zeiss Industrielle Messtechnik GmbH, Oberkochen, Germany) at magnification, ×4 objective (El-Far AHAM. et al., 2018). The total viable cell number was counted using a hemocytometer using the dye exclusion method with 0.2% trypan blue at room temperature (Thermo Fisher Scientific, Inc.). This experiment was repeated three times.

### Migration assay

Cell migration was evaluated using the monolayer denudation assay as previously described (El-Far AHAM. et al., 2018). Briefly, A549 or HepG2 cells were inoculated (5 × 10^4^ per well in 1.5 ml) and were cultured to 100% confluence in a 12-well plate. Cells were then wounded by denuding a strip of the monolayer with a 200 μl pipette tip. Cells were washed twice with PBS and then incubated with 0.75 (Fa 95) of Cur, TQ, DIM, and their double and triple combinations in 1 ml for 24 h (Table 2). The rate of wound closure was assessed in four separate fields of view using a light microscope (magnification, ×4 objective). This experiment was repeated three times.

### Colony formation assay

Two thousand five hundred cells (A549 or HepG2) per well were plated in 12-well plates and were allowed to grow for about 4–5 days until small colonies could be seen. Cells were treated with 0.75 (Fa 95) of Cur, TQ, DIM, and their double and triple combinations in 1 ml for 24 h. Also, 0.50 (Fa 95) and 0.25 (Fa 95) of Cur, TQ, and DIM were used to determine colony formation in double and triple combinations (Table 2). Cells were fixed with 4% formaldehyde in PBS and stained with 0.1% crystal violet (Guzmán et al., 2014), and images were taken for each well. This experiment was repeated three times.

### Enzyme-linked immunosorbent assay

A549 or HepG2 cells by the density of 30 × 10^4^ were cultured in a T25 flask for 48 h. Seeded cells were treated with 10 ml 0.75 (Fa 95) of Cur, TQ, DIM, and their double and triple combinations for 24 h. Also, 0.50 (Fa 95) and 0.25 (Fa 95) of Cur, TQ, and DIM (Table 2) were used to determine the effect of double and triple combinations on caspase-3, PI3K, and AKT protein levels. Cells in each flask were trypsinized and centrifuged to produce a clear cell pellet. Cells were homogenized in RIPA buffer using TissueLyser (Qiagen Co., Germantown, MD, United States). Caspase-3, PI3K, and AKT levels were determined by the FineTest ELISA kit (Wuhan Fine Biotech Co., Wuhan, Hubei, China) according to the manufacturer’s instructions at 450 nm. The Bradford method determined protein levels in all samples (Bradford, 1976).

### Chorioallantoic membrane model

The effect of Cur, TQ, DIM, and their combinations on A549 and HepG2 implants’ Chorioallantoic membrane model (CAM) of tumor weight and tumor angiogenesis were carried out as previously described by Mousa et al. (2020). Seven-day-old chick embryos were purchased from Charles River Avian Vaccine Services (Norwich, CT, United States) and incubated at 37°C with 55% relative humidity. A hypodermic needle was used to make a small hole in the shell of the air sac. A second hole was made on the broadside of the egg, directly over an avascular portion of the embryonic membrane that was identified by candling. A false air sac was created beneath the second hole by applying negative pressure at the first hole, causing the CAM to separate from the shell. A window, approximately 1.0 cm^2^, was made in the shell over the dropped CAM using a small craft grinding wheel (Dermal, Division of Emerson Electric Co., Racine, WI, United States), allowing direct access to the underlying CAM.

A549 or HepG2 cells were implanted at 1 million cells per CAM in Matrigel^®^ in the 7-day-old fertilized chick egg. Treatment effects on tumor weight and angiogenesis were determined on day 7 after tumor cell implantation. For these studies, Matrigel was thawed overnight at 4°C and placed on ice. In the exponential growth phase, cells were harvested using 0.25% trypsin–EDTA, washed, and suspended in the medium. Only suspensions of single cells with viability exceeding 95% were used. Approximately 1 × 10^6^ cells in 30 μl of medium mixed with the same volume of Matrigel were implanted on the CAM. The treatment groups were Matrigel with A549 or HepG2 cells (control), Matrigel/A549 or HepG2 with Cur (3.5 μg/CAM and 5.8 μg/CAM), TQ (6 μg/CAM and 3 μg/CAM), DIM (11.7 μg/CAM and 10.4 μg/CAM), and their combinations of Cur + DIM, Cur + TQ, TQ + DIM, and Cur + TQ + DIM (Table 2).

After incubation at 37°C with 55% relative humidity for 3 days, the CAM tissue directly beneath each filter disk was resected from control and treated CAM samples. Tissues were washed 3 times with PBS and placed in 35-mm Petri dishes. Results are presented as mean tumor weight (mg) per treatment group (*n* = 5 eggs per group) and tumor hemoglobin (Hb) (mg/dl) ± standard deviation of the mean (*n* = 5 per group).

## Results

### MTT and Fa values of Cur, TQ, and DIM against A549 cells

As shown in Figures 1D–F, MTT assay data was analyzed by CompuSyn software revealing the Fa values of Cur (Fa 0.50 = 59.73, Fa 0.75 = 33.53, Fa 0.90 = 18.82, Fa 0.95 = 12.71, and Fa 0.97 = 9.61 µM) against A549 cells. Similarly, Fa 0.50 = 124.85, Fa 0.75 = 87.88, Fa 0.90 = 61.86, Fa 0.95 = 48.71, and Fa 0.97 = 41.10 µM were determined for TQ, while Fa 0.50 = 137.26, Fa 0.75 = 102.97, Fa 0.90 = 77.25, Fa 0.95 = 63.53, and Fa 0.97 = 55.28 µM for DIM against A549 cells, as shown in Table 2.

The most exciting aspect is the combination index of Cur + TQ (Figures 1G,H), Cur + DIM (Figures 1I,J), TQ + DIM (Figures 1K,L), and Cur + TQ + DIM (Figures 1M, N), which revealed synergistic effect for Cur + TQ and moderate synergism for the rest combinations.

### Cytotoxicity percentages of Cur, TQ, and DIM against A549 cells

**FIGURE 2:**
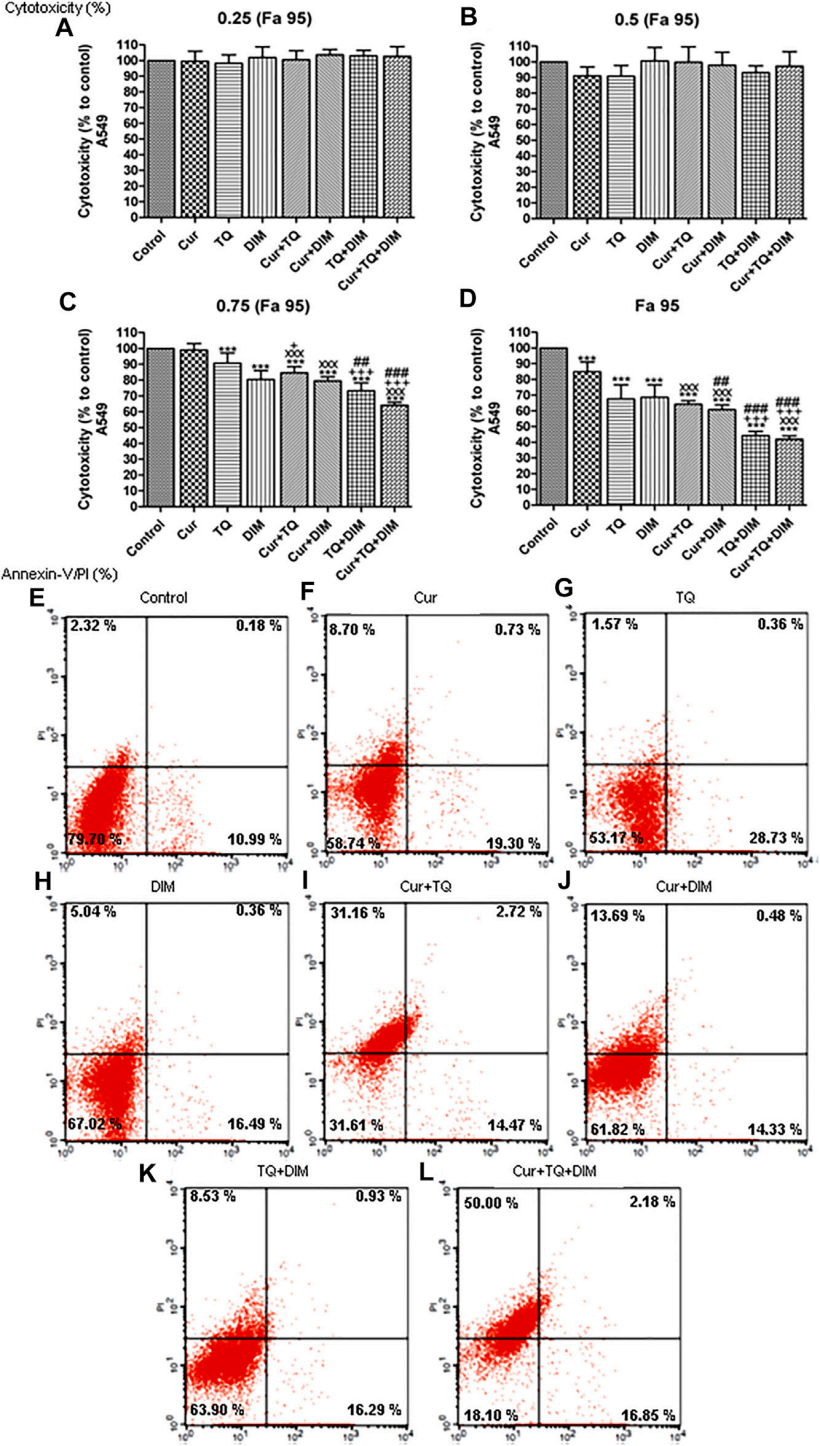
Cytotoxicity and annexin-V positive cell percentages of A549. **(A)** Cytotoxicity percentages of 0.25 (Fa 95), **(B)** 0.50 (Fa 95), **(C)** 0.75 (Fa 95), and **(D)** Fa 95 of curcumin (Cur), thymoquinone (TQ), and 3,3′-diindolylmethane (DIM) against A549. **(E)** Annexin-V positive cell percentages of control A549 cells. **(F)** Cur-treated cells with 0.75 (Fa 95) = 9.53 µM. **(G)** TQ-treated cells with 0.75 (Fa 95) = 36.53 µM. **(H)** DIM-treated cells with 0.75 (Fa 95) = 47.65 µM. **(I)** Cur + TQ treated cells with 0.75 (Fa 95) of Cur and TQ. **(J)** Cur + DIM treated cells with 0.75 (Fa 95) of Cur and DIM. **(K)** TQ + DIM treated cells with 0.75 (Fa 95) of TQ and DIM. **(L)** Cur + TQ + DIM treated cells with 0.75 (Fa 95) of Cur, TQ, and DIM. The data were analyzed with one-way ANOVA followed by Tukey’s multiple comparison test. Error bars represent mean ± SD. ^***^ *p* < 0.001 vs. control. ^xxx^ *P* < 0.001 vs. Cur. ^+^ *p* < 0.05 and ^+++^ *p* < 0.001 vs. TQ. ^##^ *p* < 0.01 and ^###^ *p* < 0.001 vs. DIM.

As shown in Figures 2A–D, Fa 95 concentrations of Cur, TQ, and DIM were used to determine the cytotoxicity percentages of Cur, TQ, DIM and their double and triple combinations. Also, 0.75 (Fa 95), 0.50 (Fa 95), and 0.25 (Fa 95) concentrations were elevated for cytotoxicity percentages compared with control untreated A549 cells. The data revealed that Cur, TQ, DIM, and their double and triple combinations at Fa 95 and 0.75 (Fa 95) concentrations significantly decreased the cytotoxicity percentages compared with control A549 cells except Cur at 0.75 (Fa 95).

### Annexin-V positive cell percentages of A549 cells treated with Cur, TQ, DIM, and their combinations

Determination of annexin-V positive cell percentages is a key marker for cell apoptosis. In Figure 2F, we can see that Cur-treated cells exhibited 28.73% of annexin-V positive cells compared with control (Figure 2E). In Figure 2G, the annexin-V positive cell percentage of TQ-treated cells is 30.84%, while DIM (Figure 2H) was 21.89% compared with control A549 cells. The combination of Cur and TQ possessed 48.35% of annexin-V positive cells (Figure 2I). Similarly, Cur + DIM (Figure 2J), TQ + DIM (Figure 2K), and Cur + TQ + DIM (Figure 2L) exhibited annexin-V positive cells of 28.50, 25.75, and 69.03%, respectively.

### Cell cycle analyses and proliferation assay of A549 cells treated with Cur, TQ, DIM, and their combinations

**FIGURE 3:**
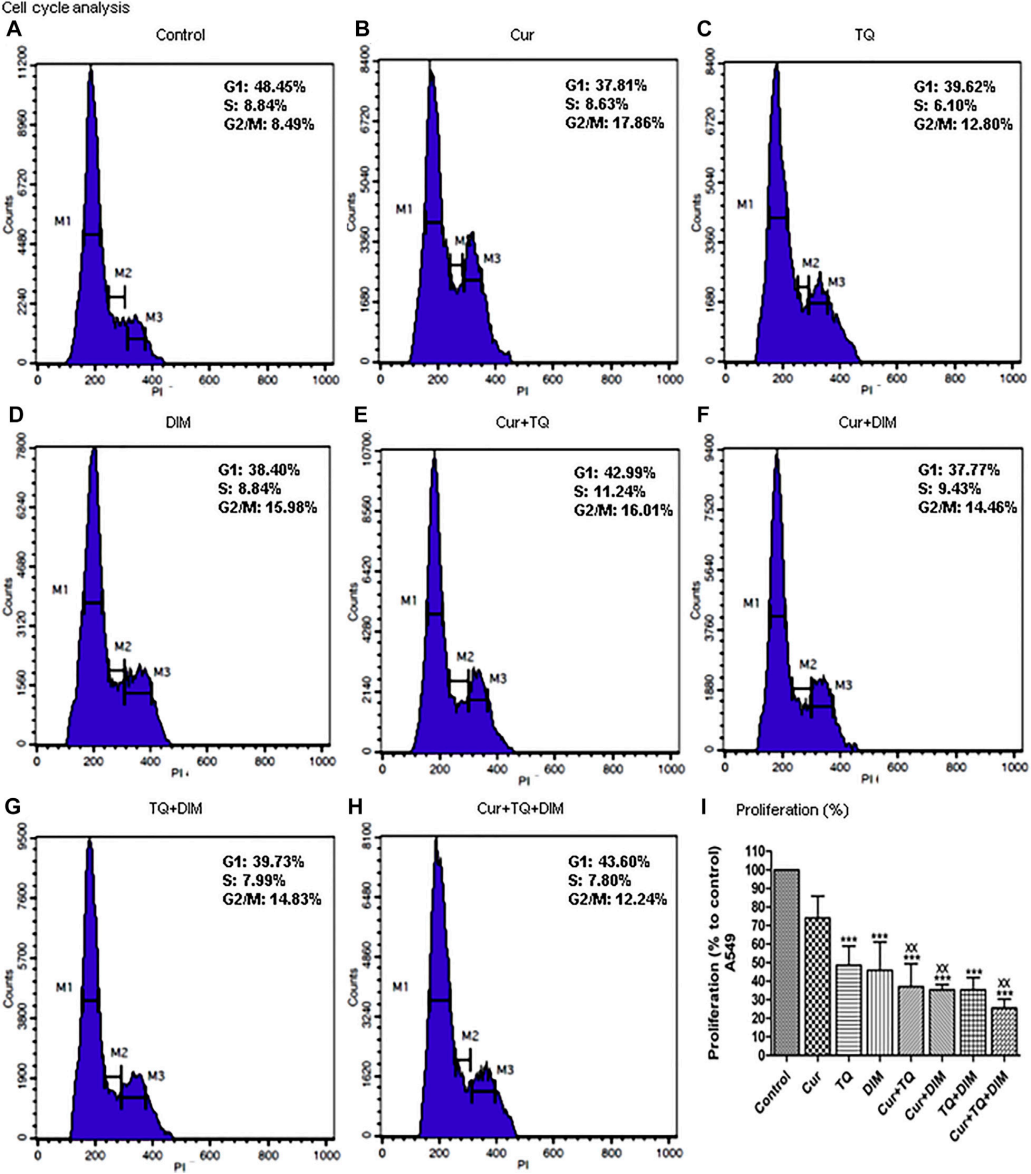
Cell cycle analysis and proliferation percentage of A549. **(A)** Cell cycle analysis of control A549. **(B)** Cell cycle analysis of Cur-treated cells with 0.75 (Fa 95) = 9.53 µM. **(C)** Cell cycle analysis of TQ-treated cells with 0.75 (Fa 95) = 36.53 µM. **(D)** Cell cycle analysis of DIM-treated cells with 0.75 (Fa 95) = 47.65 µM. **(E)** Cell cycle analysis of Cur + TQ treated cells with 0.75 (Fa 95) of Cur and TQ. **(F)** Cell cycle analysis of Cur + DIM treated cells with 0.75 (Fa 95) of Cur and DIM. **(G)** Cell cycle analysis of TQ + DIM treated cells with 0.75 (Fa 95) of TQ and DIM. **(H)** Cell cycle analysis of Cur + TQ + DIM treated cells with 0.75 (Fa 95) of Cur, TQ, and DIM. **(I)** Proliferation percentages of control and A549-treated cells with 0.75 (Fa 95) of Cur, TQ, DIM, Cur + TQ, Cur + DIM, TQ + DIM, and Cur + TQ + DIM. The data were analyzed with one-way ANOVA followed by Tukey’s multiple comparison test. Error bars represent mean ± SD. ^***^ *p* < 0.001 vs. control. ^xx^ *P* < 0.01 vs. Cur.

The results of cell cycle analysis are presented in Figures 3A–H showed S phase inhibition of A549 cells treated with Cur, TQ, DIM, Cur + TQ, Cur + DIM, TQ + DIM, and Cur + TQ + DIM by concentrations of 0.75 (Fa 95).

The results obtained from the proliferation assay of Cur-, TQ-, DIM-, Cur + TQ-, Cur + DIM-, TQ + DIM-, and Cur + TQ + DIM-treated A549 cells with concentrations of 0.75 (Fa 95) are set out in Figure 3I and revealed significant decreases in proliferation percentages of A549 cells compared with control cells.

### Migration and colony formation assays of A549 cells treated with Cur, TQ, DIM, and their combinations

**FIGURE 4:**
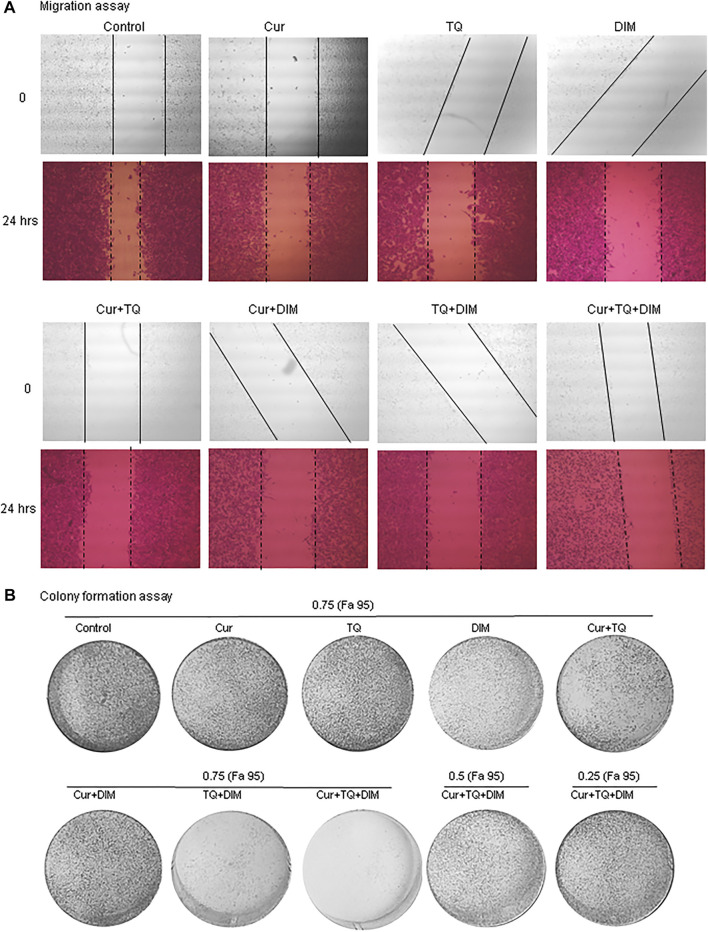
Migration and colony formation assays of A549. **(A)** Migration assay of control and A549-treated with 0.75 (Fa 95) of Cur, TQ, DIM, Cur + TQ, Cur + DIM, TQ + DIM, and Cur + TQ + DIM at 0 and 24 h. **(B)** colony formation assay of control, A549-treated with 0.75 (Fa 95) of Cur, TQ, DIM, Cur + TQ, Cur + DIM, TQ + DIM, and Cur + TQ + DIM, A549-treated with 0.50 (Fa 95) of Cur + TQ + DIM, and A549-treated with 0.25 (Fa 95) of Cur + TQ + DIM.

Migration assay was performed by induction of a wound of A549 monolayer before and 24 h after treatment with Cur, TQ, DIM, and their combinations (Figure 4A). Cur-, TQ-, DIM-, Cur + TQ-, Cur + DIM-, TQ + DIM-, and Cur + TQ + DIM-treated A549 cells with concentrations of 0.75 (Fa 95) exhibited significant reductions in migration activity. Similarly, colony formation activities of Cur-, TQ-, DIM-, Cur + TQ-, Cur + DIM-, TQ + DIM-, and Cur + TQ + DIM-treated A549 cells with concentrations of 0.75 (Fa 95), Cur + TQ + DIM at a concentration of 0.50 (Fa 95), and Cur + TQ + DIM at a concentration of 0.25 (Fa 95) were significantly decreased compared with control (Figure 4B).

### MTT and Fa values of Cur, TQ, and DIM against HepG2 cells

**FIGURE 5:**
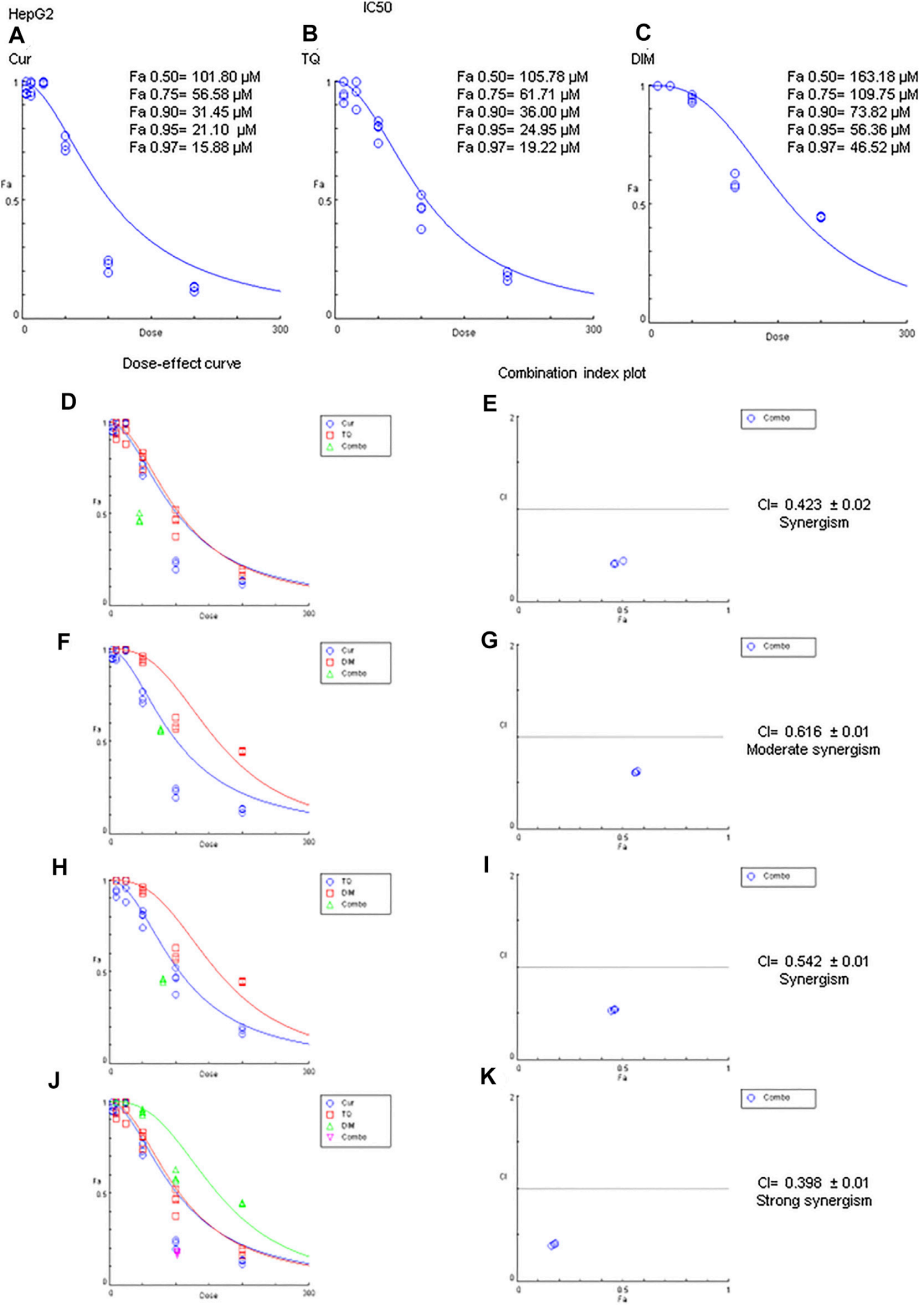
Fa values and combination index of curcumin (Cur), thymoquinone (TQ), and 3,3′-diindolylmethane (DIM) against HepG2. **(A)** Fa values of Cur against HepG2. **(B)** Fa values of TQ against HepG2. **(C)** Fa values of DIM against HepG2. **(D)** Dose-effect curve of Fa 95 of Cur (12.71 µM) and TQ (24.95 µM). **(E)** Combination index plot of Fa 95 of Cur and TQ. **(F)** Dose-effect curve of Fa 95 of Cur (21.10 µM) and DIM (56.36 µM). **(G)** Combination index plot of Fa 95 of Cur and DIM. **(H)** Dose-effect curve of Fa 95 of TQ (24.95 µM) and DIM (56.36 µM). **(H)** Combination index plot of Fa 95 of TQ and DIM. **(J)** Dose-effect curve of Fa 95 of Cur (21.10 µM), TQ (24.95 µM), and DIM (56.36 µM). **(K)** Combination index plot of Fa 95 of Cur, TQ, and DIM. The CI values represent the mean of four experiments. CI > 1.3: antagonism; CI (1.1–1.3): moderate antagonism; CI (0.9–1.1): additive effect; CI (0.8–0.9): slight synergism; CI (0.6–0.8): moderate synergism; CI (0.4–0.6): synergism; CI (0.2–0.4): strong synergism.

MTT assay has been used to determine the Fa values of Cur, TQ, and DIM against HepG2 cells (Figure 5). Fa values of Cur against HepG2 were 101.80 µM for Fa 0.50, 56.58 µM for Fa 0.75, 31.45 µM for Fa 90, 21.10 µM for Fa 0.95, and 15.88 µM for Fa 0.97 (Figure 5A). Also, Fa 0.50 = 105.78 µM, Fa 0.75 = 61.71 µM, Fa 0.90 = 36.00 µM, Fa 0.95 = 24.95 µM, and Fa 0.97 = 19.22 µM were recognized for HepG2 treated with TQ (Figure 5B). In addition, DIM exhibited Fa 0.50, Fa 0.75, Fa 0.90, Fa 0.95, and Fa 0.97 values of 163.18, 109.75, 73.82, 56.36, and 46.52 µM as shown in Figure 5C and stated in Table 2.

### Cytotoxicity percentages of Cur, TQ, and DIM against HepG2 cells

**FIGURE 6:**
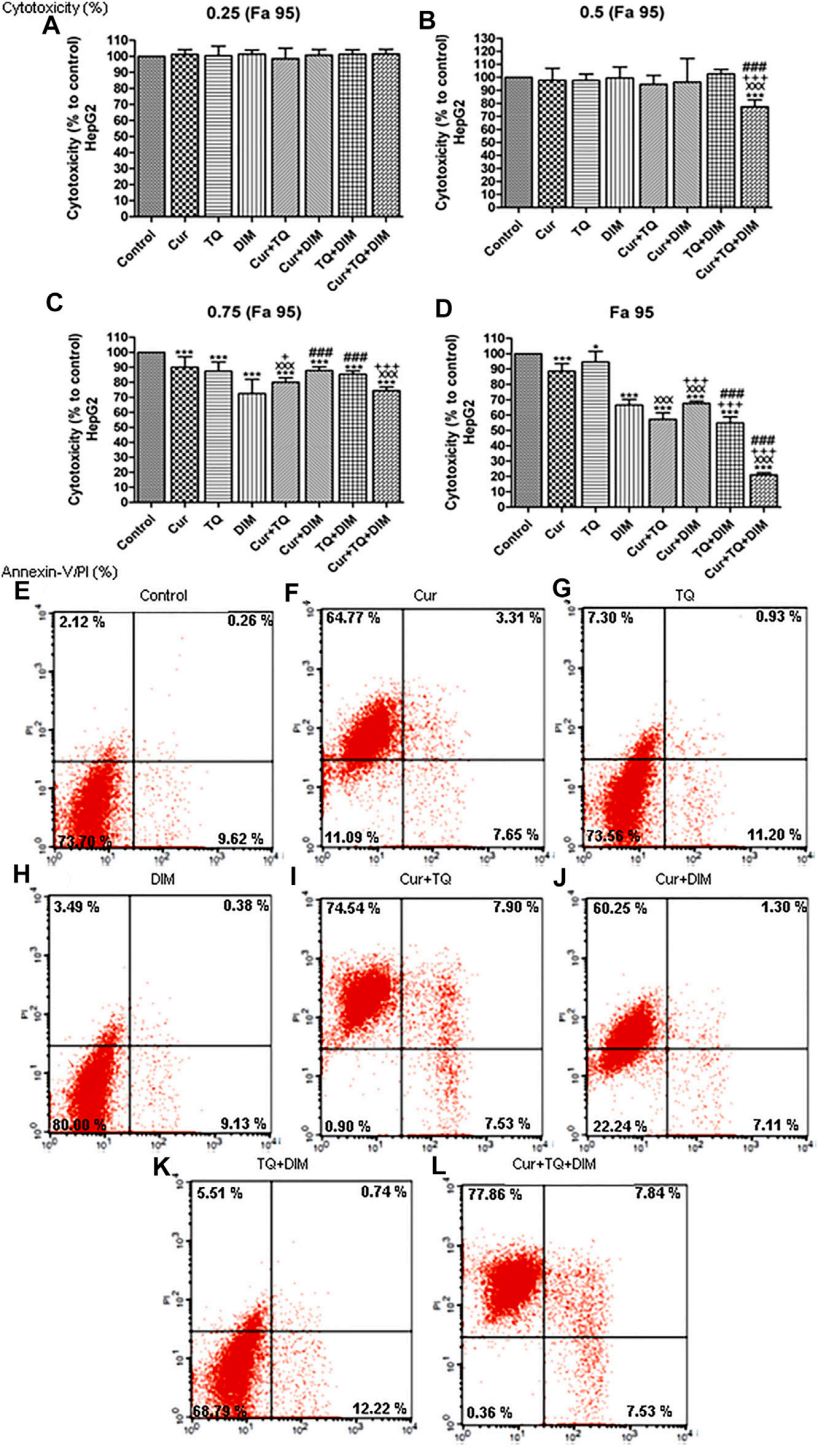
Cytotoxicity and annexin-V positive cell percentages of HepG2. **(A)** Cytotoxicity percentages of 0.25 (Fa 95) **(B)** 0.50 (Fa 95) **(C)** 0.75 (Fa 95), and **(D)** Fa 95 of curcumin (Cur), thymoquinone (TQ), and 3,3′-diindolylmethane (DIM) against HepG2. **(E)** Annexin-V positive cell percentages of control HepG2 cells. **(F)** Cur-treated cells with 0.75 (Fa 95) = 15.83 µM. **(G)** TQ-treated cells with 0.75 (Fa 95) = 18.71 µM. **(H)** DIM-treated cells with 0.75 (Fa 95) = 42.27 µM. **(I)** Cur + TQ treated cells with 0.75 (Fa 95) of Cur and TQ. **(J)** Cur + DIM treated cells with 0.75 (Fa 95) of Cur and DIM. **(K)** TQ + DIM treated cells with 0.75 (Fa 95) of TQ and DIM. **(L)** Cur + TQ + DIM treated cells with 0.75 (Fa 95) of Cur, TQ, and DIM. The data were analyzed with one-way ANOVA followed by Tukey’s multiple comparison test. Error bars represent mean ± SD. ^*^ *p* < 0.05 and ^***^ *p* < 0.001 vs. control. ^xxx^ *P* < 0.001 vs. Cur. ^+^ *p* < 0.05 and ^+++^ *p* < 0.001 vs. TQ. ^###^ *p* < 0.001 vs. DIM.

Cytotoxicity percentages of Fa 95, 0.75 (Fa 95), 0.50 (Fa 95), and 0.25 (Fa 95) of Cur, TQ, DIM, and their double and triple combinations against HepG2 cells are shown in Figures 6A–D. Compared with control, treated HepG2 cells with these drugs and combinations exhibited significant increases in cytotoxicity percentages.

### Annexin-V positive cell percentages of HepG2 cells treated with Cur, TQ, DIM, and their combinations

Results shown in Figures 6E–L treated HepG2 cells with Cur, TQ, DIM, Cur + TQ, Cur + DIM, TQ + DIM, and Cur + TQ + DIM exhibited significant increases in annexin-V positive cells by percentages of 75.73, 19.43, 13.00, 89.97, 68.66, 18.47, and 93.23, respectively compared with 12.00 of control.

### Cell cycle analyses and proliferation assay of HepG2 cells treated with Cur, TQ, DIM, and their combinations

**FIGURE 7:**
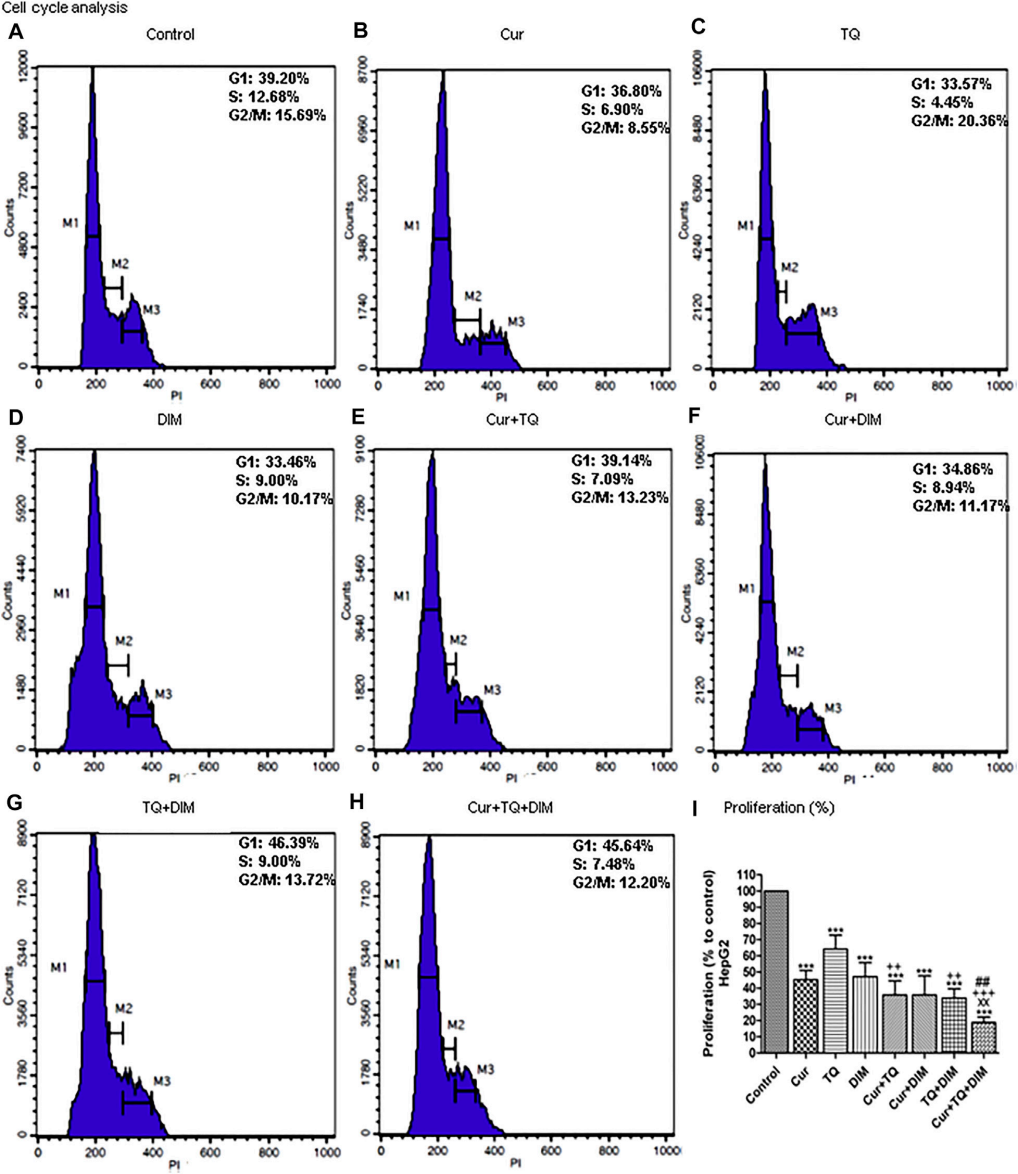
Cell cycle analysis and proliferation percentage of HepG2. **(A)** Cell cycle analysis of control HepG2. **(B)** Cell cycle analysis of Cur-treated cells with 0.75 (Fa 95) = 15.83 µM. **(C)** Cell cycle analysis of TQ-treated cells with 0.75 (Fa 95) = 18.71 µM. **(D)** Cell cycle analysis of DIM-treated cells with 0.75 (Fa 95) = 42.27 µM. **(E)** Cell cycle analysis of Cur + TQ treated cells with 0.75 (Fa 95) of Cur and TQ. **(F)** Cell cycle analysis of Cur + DIM treated cells with 0.75 (Fa 95) of Cur and DIM. **(G)** Cell cycle analysis of TQ + DIM treated cells with 0.75 (Fa 95) of TQ and DIM. **(H)** Cell cycle analysis of Cur + TQ + DIM treated cells with 0.75 (Fa 95) of Cur, TQ, and DIM. **(I)** Proliferation percentages of control and HepG2-treated cells with 0.75 (Fa 95) of Cur, TQ, DIM, Cur + TQ, Cur + DIM, TQ + DIM, and Cur + TQ + DIM. The data were analyzed with one-way ANOVA followed by Tukey’s multiple comparison test. Error bars represent mean ± SD. ^***^ *p* < 0.001 vs. control. ^xx^ *P* < 0.01 vs. Cur. ^++^ *p* < 0.01 and ^++^ *p* < 0.01 vs. TQ. ^##^ *p* < 0.01 vs. DIM.

Cell cycle analysis of HepG2 cells treated with Cur, TQ, DIM, Cur + TQ, Cur + DIM, TQ + DIM, and Cur + TQ + DIM by 0.75 (Fa 95) doses showed S phase inhibition (Figures 7A–H). Concentrations of these drugs significantly decreased proliferation percentages of HepG2 cells (Figure 7I).

### Migration and colony formation assays of HepG2 cells treated with Cur, TQ, DIM, and their combinations

**FIGURE 8:**
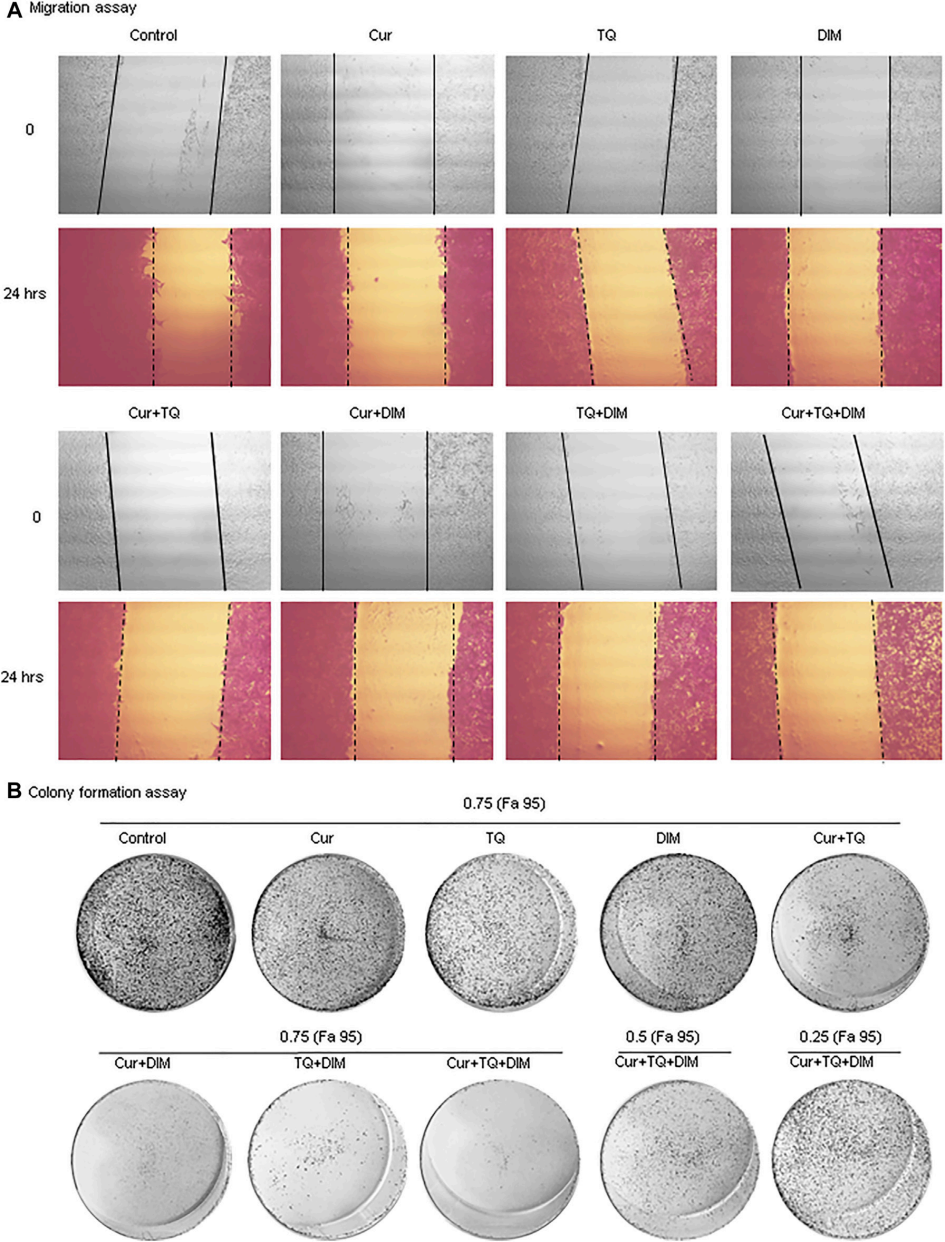
Migration and colony formation assays of HepG2. **(A)** Migration assay of control and HepG2-treated with 0.75 (Fa 95) of Cur, TQ, DIM, Cur + TQ, Cur + DIM, TQ + DIM, and Cur + TQ + DIM at 0 and 24 h **(B)** colony formation assay of control, HepG2-treated with 0.75 (Fa 95) of Cur, TQ, DIM, Cur + TQ, Cur + DIM, TQ + DIM, and Cur + TQ + DIM, HepG2-treated with 0.50 (Fa 95) of Cur + TQ + DIM, and HepG2-treated with 0.25 (Fa 95) of Cur + TQ + DIM.

Cur-, TQ-, DIM-, Cur + TQ-, Cur + DIM-, TQ + DIM-, and Cur + TQ + DIM-treated HepG2 cells with concentrations of 0.75 (Fa 95) exhibited significant decreases in migration activity as presented in Figure 8A. By the same concentrations, colony formation activities of the Cur-, TQ-, DIM-, Cur + TQ-, Cur + DIM-, TQ + DIM-, and Cur + TQ + DIM-treated HepG2 cells with concentrations of 0.75 (Fa 95), Cur + TQ + DIM at concentrations of 0.50 (Fa 95), and 0.25 (Fa 95) were significantly decreased compared with control (Figure 8B).

### Caspase-3, PI3K, and AKT protein levels in A549 and HepG2 cells treated with Cur, TQ, DIM, and their combinations

**FIGURE 9:**
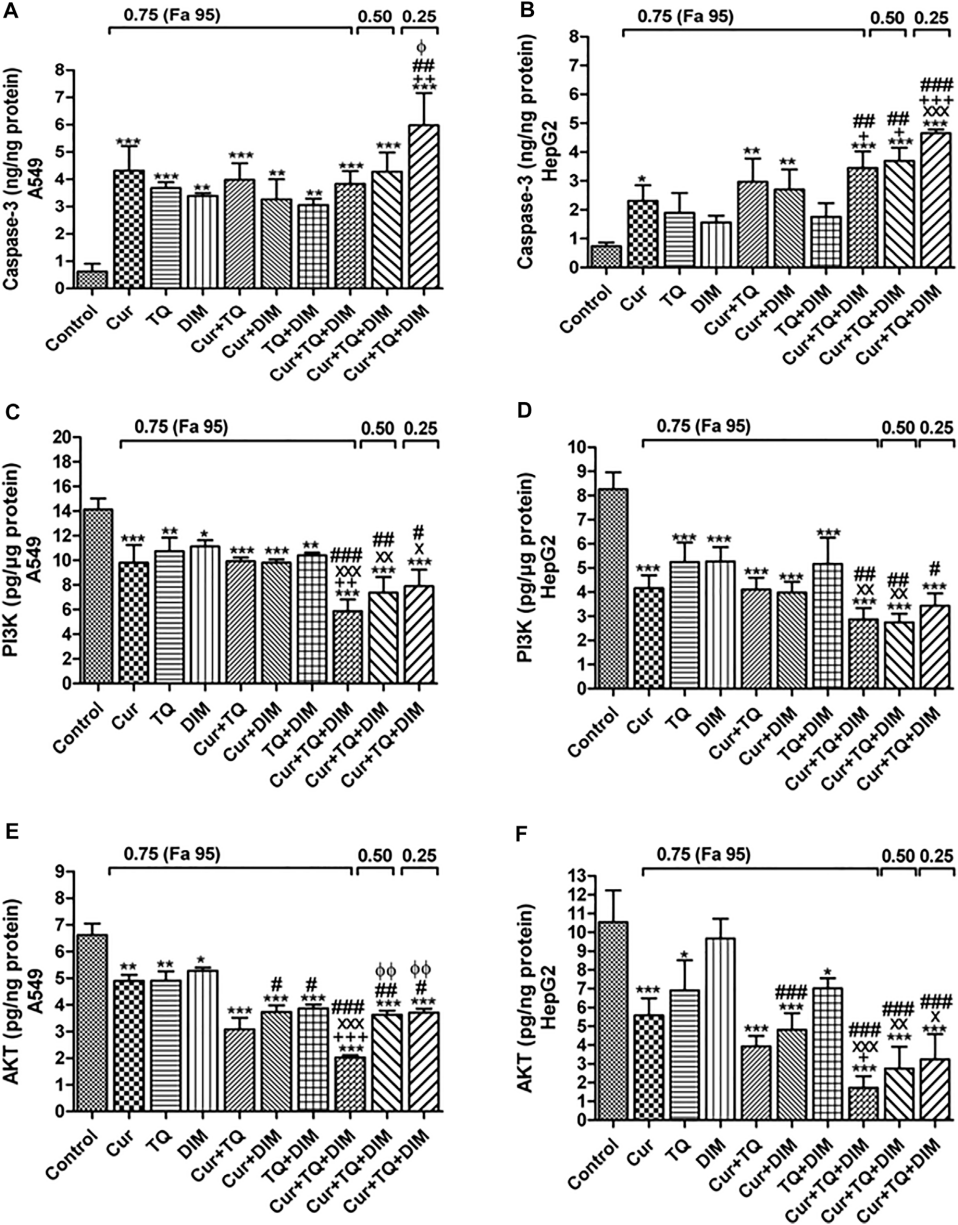
Enzyme-linked immunosorbent (ELISA) assay. **(A)** Caspase-3 (ng/ng protein) **(C)** phosphatidylinositol 3-kinase (PI3K, pg/µg protein), and **(E)** Protein kinase B (AKT, pg/ng protein) levels in control, A549-treated with 0.75 (Fa 95) of Cur, TQ, DIM, Cur + TQ, Cur + DIM, TQ + DIM, and Cur + TQ + DIM, A549-treated with 0.50 (Fa 95) of Cur + TQ + DIM, and A549-treated with 0.25 (Fa 95) of Cur + TQ + DIM. **(B)** Caspase-3 (ng/ng protein) **(D)** PI3K (pg/µg protein), and **(F)** AKT (pg/ng protein) levels in control, HepG2-treated with 0.75 (Fa 95) of Cur, TQ, DIM, Cur + TQ, Cur + DIM, TQ + DIM, and Cur + TQ + DIM, HepG2-treated with 0.50 (Fa 95) of Cur + TQ + DIM, and HepG2-treated with 0.25 (Fa 95) of Cur + TQ + DIM. The data were analyzed with one-way ANOVA followed by Tukey’s multiple comparison test. Error bars represent mean ± SD. ^*^ *p* < 0.05, ^**^ *p* < 0.01, and ^***^ *p* < 0.001 vs. control. ^x^ *P* < 0.05, ^xx^ *P* < 0.01, and ^xxx^ *P* < 0.001 vs. Cur. ^+^ *p* < 0.05, ^++^ *p* < 0.01, and ^+++^ *p* < 0.001 vs. TQ. ^#^ *p* < 0.05, ^##^ *p* < 0.01, and ^###^ *p* < 0.001 vs. DIM.

It can be seen from the data in Figure 9A that caspase-3 protein levels were significantly increased in Cur-, TQ-, DIM-, Cur + TQ-, Cur + DIM-, TQ + DIM-, and Cur + TQ + DIM-treated A549 cells with concentrations of 0.75 (Fa 95), Cur + TQ + DIM at concentrations of 0.50 (Fa 95), and 0.25 (Fa 95). A549 cells treated with the same concentrations exhibited significant decreases in the PI3K (Figure 9C) and AKT (Figure 9E) protein levels compared with control.

Caspase-3 protein levels were significantly increased in the Cur-, TQ-, DIM-, Cur + TQ-, Cur + DIM-, TQ + DIM-, and Cur + TQ + DIM-treated HepG2 cells with concentrations of 0.75 (Fa 95), Cur + TQ + DIM at a concentration of 0.50 (Fa 95), and Cur + TQ + DIM at a concentration of 0.25 (Fa 95) (Figure 9B). On the contrary, HepG2 cells treated with the same concentrations exhibited significantly decreased PI3K (Figure 9D) and AKT (Figure 9F) protein levels compared with control.

### Tumor weight and hemoglobin concentrations of A549 and HepG2 implant in CAM

**FIGURE 10:**
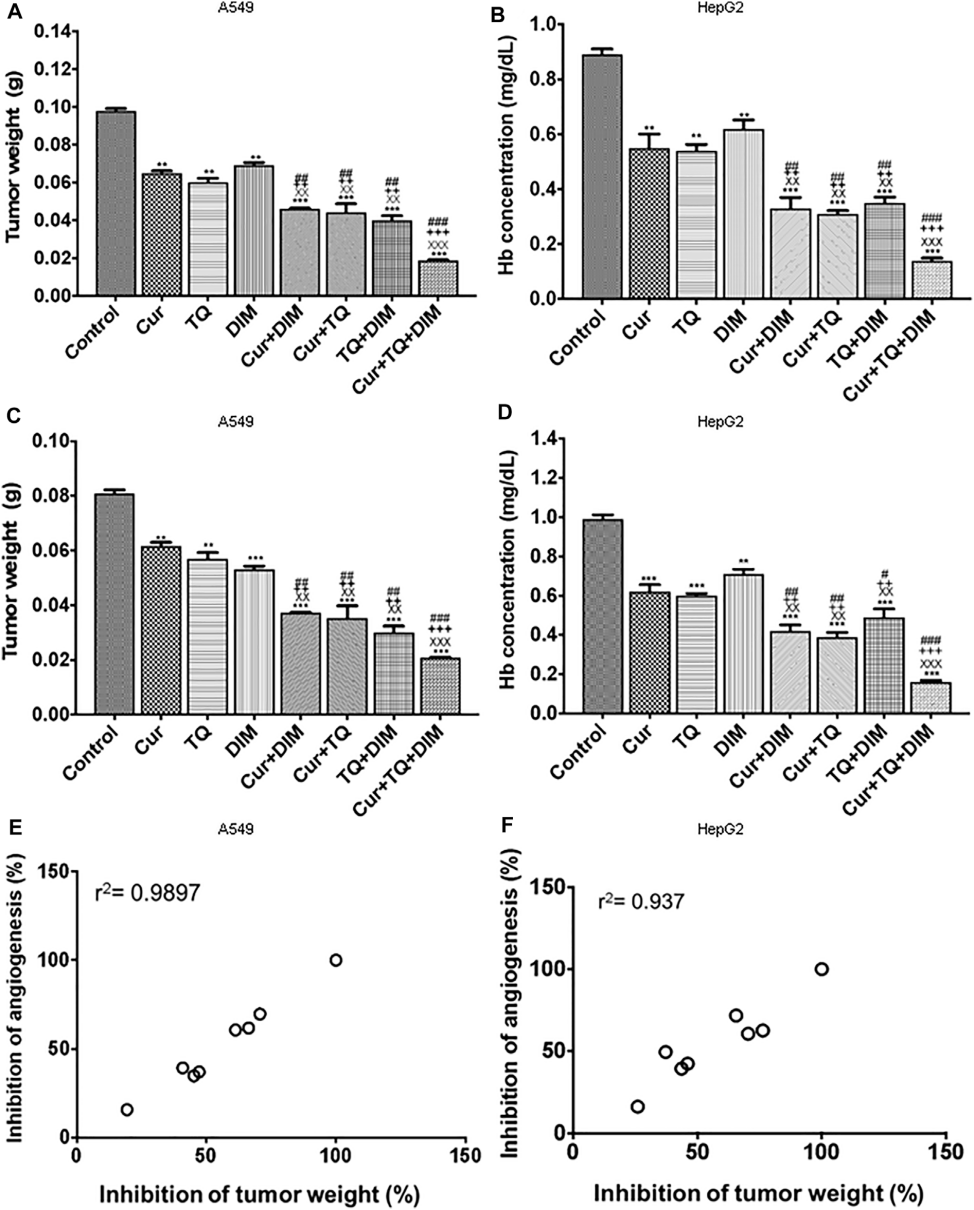
Chorioallantoic membrane (CAM) model. **(A)** Tumor weight per g for A549 implant **(B)** Hemoglobin concentration (mg/ml) for A549 implant **(C)** Tumor weight per g for HepG2 implant, and **(D)** Hemoglobin concentration (mg/ml) for HepG2 implant. **(E)** Correlation coefficient (*r* ^2^) of percentages of inhibition of tumor weight and hemoglobin concentrations for A549 implant. **(F)** Correlation coefficient (*r* ^2^) of percentages of inhibition of tumor weight and hemoglobin concentrations for HepG2 implant. The treatment groups were Matrigel with A549 or HepG2 cells (control), Matrigel/A549 or HepG2 with Cur (3.5 μg/CAM and 5.8 μg/CAM), TQ (6 μg/CAM and 3 μg/CAM), DIM (11.7 μg/CAM and 10.4 μg/CAM), and their combinations of Cur + DIM, Cur + TQ, TQ + DIM, and Cur + TQ + DIM. The data were analyzed with one-way ANOVA followed by Tukey’s multiple comparison test. Error bars represent mean ± SD. ^**^ *p* < 0.01 and ^***^ *p* < 0.001 vs. control. ^xx^ *P* < 0.01 and ^xxx^ *P* < 0.001 vs. Cur. ^++^ *p* < 0.01 and ^+++^ *p* < 0.001 vs. TQ. ^#^ *p* < 0.05, ^##^ *p* < 0.01, and ^###^ *p* < 0.001 vs. DIM.

Tumor weights and Hb concentrations of A549 (Figures 10A,B, respectively) and HepG2 (Figures 10C,D, respectively) cells’ implants treated with Cur, TQ, DIM, and their combinations significantly decreased compared with the control group. The tumor weights of implanted A549 cells were significantly decreased Cur (0.0618 ± 0.0012 g), TQ (0.0570 ± 0.0023 g), DIM (0.0531 ± 0.0012 g), Cur + TQ (0.0353 ± 0.0045 g) Cur + DIM (0.0373 ± 0.0001 g), TQ + DIM (0.0301 ± 0.0023 g), and Cur + TQ + DIM (0.0210 ± 0.0001 g) than control (0.0810 ± 0.0012 g). Similary, HepG2 implants exhibited significant decreases in the Cur (0.0650 ± 0.0012 g), TQ (0.0600 ± 0.0023 g), DIM (0.0693 ± 0.0012 g), Cur + TQ (0.0443 ± 0.0045 g) Cur + DIM (0.0463 ± 0.0001 g), TQ + DIM (0.0401 ± 0.0023 g), and Cur + TQ + DIM (0.0190 ± 0.0001 g) tumor weight than control (0.0980 ± 0.0012 g).

The Cur + TQ + DIM, Cur + DIM, Cur + TQ, and TQ + DIM treated implants exhibited more significant reductions in tumor weights and Hb concentrations of A549 and HepG2 cells’ implants compared with single drugs, Cur, TQ, and DIM. Excellent correlation (*r* ^2^ = 0.99) between tumor growth inhibition and tumor angiogenesis inhibition was demonstrated for the various natural bioactive compounds and their combinations, which suggest a key role for their anti-angiogenesis activities in suppressing tumor progression (Figures 10E,F).

## Discussion

Nutraceuticals are derived from various natural sources such as medicinal plants, marine organisms, vegetables, and fruits (Kuppusamy et al., 2014). Many food components have been associated with chemopreventive properties, including Cur, TQ, DIM, lycopene, resveratrol, ellagic acid, and costunolide (El-Far AH. et al., 2018, 2020a, 2020b, El-Far et al., 2021 AH., 2021b; Mousa et al., 2020; Sudha et al., 2020).

By searching in the comparative toxicogenomics database (http://ctdbase.org/), we stated Cur, TQ, and DIM’s anticancer effect against numerous cancer types targeting numerous genes represented in Supplementary Tables S4–S6, respectively. To explain the combinatory effect of Cur, TQ, and DIM, we summarized the effect of them and their targets in Supplementary Table S7. Cur and TQ, Cur and DIM, and the three drugs have common targets that explain their combinatory anticancer, which increased in double and highly increased in the triple combination. On the contrary, no common targets were recognized for TQ and DIM, suggesting they did their effect by different mechanisms if used together.

In the present study, Cur, TQ, and DIM combinations significantly induced apoptosis and increased caspase-3 protein levels while significantly decreasing cell proliferation, migration, colony formation activities, and PI3K and AKT protein levels in A549 and HepG2 with S phase reduction in both cells. Also, caspase-3 levels were expressed at low levels in the patient’s samples, while PI3K and AKT were expressed at high levels (Supplementary Figure S1).

Lung cancer is one of the leading causes of cancer-related deaths in males and females worldwide. Approximately 85% of lung cancer cases are diagnosed as non-small-cell lung cancer (Molina et al., 2008). Numerous studies have investigated the anticancer effect of Cur against A549 cells through downregulation of urothelial cancer-associated 1 (Wang WH. et al., 2018). Also, Cur induced Bad dephosphorylation in an AKT-dependent fashion in A549 cells (Endo et al., 2020). In addition, Cur induced phosphorylation and activation of c-Jun N-terminal kinase, p38, and extracellular signal-regulated kinase (ERK) (Yao et al., 2015) and activation of autophagy (Liu et al., 2017) in A549. Fluorescent liposomes containing a combination of TQ and Cur decreased A549 lung cancer cell viability (Fahmy, 2019). Another nanoformulation of Cur-chrysin-loaded alginate-chitosan hydrogels significantly induced apoptosis and G2/M cell cycle arrest in both A549 and T47D cell lines (Abbasalizadeh et al., 2021). Also, the cell cycle has been arrested by the Cur and Cur/paclitaxel combination in A549 cells (Lee et al., 2020). In another study, Cur increased the sensitivity of A549 cells to gemcitabine therapy. It significantly decreased the migration and invasion of A549/gemcitabine cells in response to reduced expression of matrix metalloproteinase-9, vimentin, and N-cadherin (Dong et al., 2021). By another mechanism, Cur inhibited A549 cells migration and invasion through suppression of PI3K/AKT/mammalian target of rapamycin (mTOR) signaling pathway (Wang et al., 2020).

TQ promoted apoptosis in A549 cells by the activation of p53 and caspase cascade-dependent pathways (Samarghandian et al., 2019), and reactive oxygen species (ROS) generation (Alhakamy et al., 2020), and extracellular signal-regulated kinase 1/2 pathway (Yang et al., 2015). Furthermore, TQ significantly reduced the expression of B-cell lymphoma 2 (Bcl2), an anti-apoptotic protein, and induced Bcl2-associated X (Bax) protein, a proapoptotic, leading to apoptosis of A549 cells (Alam et al., 2022). In addition, the p-AKT, p-mTOR, caspase-3, p-53, and NF-κB expression levels were significantly reduced in TQ and TQ/indirubin-3-monoxime-treated A549 cells (Dera et al., 2020). Also, TQ induced the antimitotic mechanism of A549 cells by its direct binding to the tubulin-MT network (Acharya et al., 2014). Khan et al. (2022) treated A549 with polyethylene glycol (PEG)-coated DSPC/cholesterol comprising TQ liposomes (PEG-Lip-TQ) that induced several-fold decreases in the PEG-Lip-TQ’s IC_50_ and showed changes in cell cycle analysis in comparison with free TQ. In the same context, TQ nanoformulations induced cell cycle arrest of A549 (Alhakamy et al., 2020; Asfour et al., 2021). Also, transferrin-decorated thymoquinone-loaded PEG-PLGA nanoparticles restricted the A549 cells migration (Upadhyay et al., 2019). Furthermore, as confirmed by Western blot analysis, Yang et al. (2015) stated that TQ inhibited A549 cell proliferation, migration, and invasion through the ERK1/2 pathway.

Cur induced HepG2 cells’ death by increasing apoptosis and ROS generation process (Liang et al., 2021). This effect could be attributed to the activation of caspase-3 (Dai et al., 2013) and the downregulation of the PI3K/AKT signaling pathway (Chang et al., 2017; Han et al., 2020). In addition, Cur induced mitochondrial and mitochondrial DNA damage (Cao et al., 2007) besides fatty acid synthase inhibition (Fan et al., 2014) in HepG2. In addition, Cur significantly inhibited HepG2 cell migration, as stated by Duan et al. (2014), Zhao et al. (2016), and Wang L. et al (2018).

TQ induced G2/M phase cell cycle arrest and apoptosis in HepG2 cells with a significant increase in Bax/Bcl2 ratio (ElKhoely et al., 2015). HepG2 treated with TQ exhibited significant apoptosis explained in many studies. It could be due to increased miR-16 and miR-375 levels and caspase-3 and Bcl2 expressions (Bashir et al., 2020). Also, HepG2 treated with TQ exhibited significant increases in caspase-3 (Aslan et al., 2021) and decreased AKT (Attoub et al., 2013) levels. In addition, TQ enhanced the TRAIL-induced death of HepG2 cells and inhibited NF-κB (Ashour et al., 2014).

DIM inhibited HepG2 cell proliferation in a concentration- and time-dependent (Jiang et al., 2019). Also, DIM induced S-phase retardation and mitotic delay in HepG2 cells through topoisomerase II-*α* catalytic inhibition (Gong et al., 2006).

Angiogenesis is a multistep process triggered by various biological signals and has a physiological and pathological role (Park et al., 2020). Angiogenesis is regulated by balancing angiogenic growth and inhibitory factors in healthy tissues (Al-Ostoot et al., 2021). Neovascularization in tumor masses is caused by vascular endothelial growth factor (VEGF), angiopoietin families, and cytokines (Saaristo et al., 2000).

In the current study, the CAM system has been used to investigate Cur, TQ, and DIM’s anti-angiogenic effect and their combinations. Triple and double combinations significantly reduced tumor weight and Hb concentrations in tumor masses. The CAM system has been widely used to study human tumor growth (Klingenberg et al., 2014; Herrmann et al., 2016; Mousa et al., 2020). Also, the anti-angiogenic effect of Cur (Deng et al., 2016; Buzzá et al., 2019), TQ (Shanmugam et al., 2018; Ndreshkjana et al., 2019), and DIM (Mousa et al., 2020) were applied in CAM. Furthermore, many studies reported the anti-angiogenic effect of Cur and TQ against the A549 and HepG2 mouse xenograft model leading to reductions in tumor growth and vascularization targeting VEGF (Yoysungnoen et al., 2006, 2008; ElKhoely et al., 2015; Wang et al., 2015; Jiao et al., 2016; Li et al., 2018; Tian et al., 2021). Our data supported the key role of Cur, TQ, and DIM and their combinations on angiogenesis suppression and its highly correlated effects on tumor growth inhibition.

The current study stated that Cur, TQ, and DIM increased A549 and HepG2 apoptosis. Interestingly, double and triple combinations of Cur, TQ, and DIM induced more apoptosis of A549 and HepG2 with significant decreases in proliferation, migration, and colony formation activities. This action could be attributed to the enhanced effects of Cur, TQ, and DIM combinations.

## Conclusion and prospective

The present study was designed to determine the combinatory effect of Cur, TQ, and DIM on A549 and HepG2 cells. The most obvious finding from this study is that triple (Cur + TQ + DIM) and double (Cur + TQ, Cur + DIM, and TQ + DIM) combinations increased apoptosis and decreased proliferation and migration colony formation activities with S phase reduction. In addition, triple and double combinations significantly reduced tumor weight and Hb concentrations in A549 and HepG2 cells’ implants in the CAM model. Therefore, Cur, TQ, and DIM combinations are promising agents for suppressing tumor growth and angiogenesis. Further preclinical and clinical studies are warranted trying to use their approved doses as stated in Supplementary Tables S1–S3.

Cur, TQ, and DIM have common targets as stated in Supplementary Table S7, except TQ and DIM have not shared targets. For future consideration, we determined the binding free energy, binding affinity (p*Ki*), and the ligand efficiency of common targets with Cur, TQ, and DIM have been determined using InstaDock software (Hassan et al., 2017) and represented in Table 3. Generally, Cur has the highest binding affinities with target proteins, followed by DIM and then TQ. Furthermore, because Cur, TQ, and DIM have different and common targets, we suggest treating different cancer cells with these drugs with time intervals even for 1 h in between and determine the most effective combination for each cancer types, especially mitogen-activated protein kinase (MAPK), matrix metalloproteinases (MMPs), and AKT/PI3K/mTOR pathways, as stated in Table 3. In the same context, we encourage researchers to investigate Cur, TQ, and DIM combination with chemotherapeutic agents investigating the effect of natural products combined with commonly used chemotherapeutics for cancer therapy.

**TABLE 3:** Docking score of curcumin (Cur), thymoquinone (TQ), and 3, 3′-diindolylmethane (DIM) against some of their common targets.

	Cur			TQ			DIM		
	Binding Free Energy (kcal/mol)	Binding affinity (p*Ki*)	Ligand Efficiency (kcal/mol/non-H atom)	Binding Free Energy (kcal/mol)	Binding affinity (p*Ki*)	Ligand Efficiency (kcal/mol/non-H atom)	Binding Free Energy (kcal/mol)	Binding affinity (p*Ki*)	Ligand Efficiency (kcal/mol/non-H atom)
AKT1 (4ekk)	−8.40	6.16	0.31	−6.30	4.62	0.53	−8.10	5.94	0.43
CASP8 (1i4e)	−6.60	4.84	0.24	−6.10	4.47	0.51	−7.60	5.57	0.40
CDK4 (2w96)	−6.50	4.77	0.24	−5.60	4.11	0.47	−7.30	5.35	0.38
CYP1A1 (6o5y)	−9.70	7.11	0.36	−6.50	4.77	0.54	−9.00	6.60	0.47
CYP1A2 (2hi4)	−9.10	6.67	0.34	−5.90	4.33	0.49	−10.60	7.77	0.56
CYP1B1 (6iq5)	−9.10	6.67	0.34	−7.70	5.65	0.64	−10.20	7.48	0.54
HMOX1 (6eha)	−7.50	5.50	0.28	−6.60	4.84	0.55	−8.80	6.45	0.46
JAK2 (7f7w)	−8.50	6.23	0.31	−5.70	4.18	0.48	−7.50	5.50	0.39
KDR (7juy)	−7.50	5.50	0.28	−5.30	3.89	0.44	−8.20	6.01	0.43
MAPK1 (1tvo)	−7.20	5.28	0.27	−5.50	4.03	0.46	−7.90	5.79	0.42
MAPK3 (6ges)	−7.50	5.50	0.28	−5.40	3.96	0.45	−8.00	5.87	0.42
MMP2 (1ck7)	−7.00	5.13	0.26	−5.90	4.33	0.49	−9.10	6.67	0.48
MMP9 (1gkc)	−8.30	6.09	0.31	−6.40	4.69	0.53	−8.60	6.31	0.45
MPO (5wdj)	−7.30	5.35	0.27	−6.10	4.47	0.51	−9.50	6.97	0.50
mTOR (4jsv)	−7.30	5.35	0.27	−5.80	4.25	0.48	−8.90	6.53	0.47
NME1 (5ui4)	−8.00	5.87	0.30	−6.00	4.40	0.50	−8.00	5.87	0.42
NQO1 (2f1o)	−6.60	4.84	0.24	−6.80	4.99	0.57	−7.70	5.65	0.41
PARP1 (1uk0)	−9.50	6.97	0.35	−6.20	4.55	0.52	−8.80	6.45	0.46
PIK3CA (7r9y)	−8.90	6.53	0.33	−6.30	4.62	0.53	−8.50	6.23	0.45
TALDO1 (1f05)	−6.60	4.84	0.24	−5.10	3.74	0.43	−7.10	5.21	0.37
TOP2A (1zxm)	−7.40	5.43	0.27	−5.70	4.18	0.48	−8.20	6.01	0.43
TYMS (1hzw)	−7.10	5.21	0.26	−5.50	4.03	0.46	−8.30	6.09	0.44

## Data Availability

The original contributions presented in the study are included in the article/Supplementary Material, further inquiries can be directed to the corresponding author.

## References

[B1] Abbasalizadeh F. Alizadeh E. Bagher Fazljou S. M. Torbati M. Akbarzadeh A. (2022). Anticancer effect of alginate-chitosan hydrogel loaded with Curcumin and Chrysin on lung and breast cancer cell lines. Cdd 19, 600–613. 10.2174/1567201818666210813142007 34391378

[B2] Acharya B. R. Chatterjee A. Ganguli A. Bhattacharya S. Chakrabarti G. (2014). Thymoquinone inhibits microtubule polymerization by tubulin binding and causes mitotic arrest following apoptosis in A549 cells. Biochimie 97, 78–91. 10.1016/j.biochi.2013.09.025 24113316

[B3] Al-Ostoot F. H. Salah S. Khamees H. A. Khanum S. A. (2021). Tumor angiogenesis: Current challenges and therapeutic opportunities. Cancer Treat. Res. Commun. 28, 100422. 10.1016/j.ctarc.2021.100422 34147821

[B4] Alam S. Mohammad T. Padder R. A. Hassan M. I. Husain M. (2022). Thymoquinone and quercetin induce enhanced apoptosis in non-small cell lung cancer in combination through the Bax/Bcl2 cascade. J. Cell. Biochem. 123, 259–274. 10.1002/jcb.30162 34636440

[B5] Alhakamy N. A. Badr-Eldin S. M. A Fahmy U. U. Alruwaili N. K. Awan Z. A. Caruso G. (2020). Thymoquinone-loaded soy-phospholipid-based phytosomes exhibit anticancer potential against human lung cancer cells. Pharmaceutics 12, 761. 10.3390/pharmaceutics12080761 PMC7463966 32806507

[B6] Alhmied F. Alammar A. Alsultan B. Alshehri M. Pottoo F. H. (2021). Molecular mechanisms of thymoquinone as anticancer agent. Comb. Chem. High. Throughput Screen. 24, 1644–1653. 10.2174/1386207323999201027225305 33115388

[B7] Asfour H. Z. Fahmy U. A. Alharbi W. S. Almehmady A. M. Alamoudi A. J. Tima S. (2021). Phyto-phospholipid conjugated scorpion venom nanovesicles as promising carrier that improves efficacy of thymoquinone against adenocarcinoma human alveolar basal epithelial cells. Pharmaceutics 13, 2144. 10.3390/pharmaceutics13122144 34959424 PMC8709205

[B8] Ashour A. E. Abd-Allah A. R. Korashy H. M. Attia S. M. Alzahrani A. Z. Saquib Q. (2014). Thymoquinone suppression of the human hepatocellular carcinoma cell growth involves inhibition of IL-8 expression, elevated levels of TRAIL receptors, oxidative stress and apoptosis. Mol. Cell. Biochem. 389, 85–98. 10.1007/s11010-013-1930-1 24399465

[B9] Aslan M. Afşar E. Kırımlıoglu E. Çeker T. Yılmaz Ç. (2021). Antiproliferative effects of thymoquinone in MCF-7 breast and HepG2 liver cancer cells: Possible role of ceramide and ER stress. Nutr. Cancer 73, 460–472. 10.1080/01635581.2020.1751216 32286088

[B10] Attoub S. Sperandio O. Raza H. Arafat K. Al-Salam S. Al Sultan M. A. (2013). Thymoquinone as an anticancer agent: Evidence from inhibition of cancer cells viability and invasion *in vitro* and tumor growth *in vivo* . Fundam. Clin. Pharmacol. 27, 557–569. 10.1111/j.1472-8206.2012.01056.x 22788741

[B11] Bashir A. O. El-Mesery M. E. Anwer R. Eissa L. A. (2020). Thymoquinone potentiates miR-16 and miR-375 expressions in hepatocellular carcinoma. Life Sci. 254, 117794. 10.1016/j.lfs.2020.117794 32422307

[B12] Bradford M. M. (1976). A rapid and sensitive method for the quantitation of microgram quantities of protein utilizing the principle of protein-dye binding. Anal. Biochem. 72, 248–254. 10.1006/abio.1976.9999 942051

[B13] Bray F. Laversanne M. Weiderpass E. Soerjomataram I. (2021). The ever-increasing importance of cancer as a leading cause of premature death worldwide. Cancer 127, 3029–3030. 10.1002/cncr.33587 34086348

[B14] Buzzá H. H. Fialho de Freitas L. C. Moriyama L. T. Teixeira Rosa R. G. Bagnato V. S. Kurachi C. (2019). Vascular effects of photodynamic therapy with curcumin in a chorioallantoic membrane model. Int. J. Mol. Sci. 20, 1084. 10.3390/ijms20051084 PMC6429090 30832361

[B15] Cao J. Liu Y. Jia L. Zhou H. M. Kong Y. Yang G. (2007). Curcumin induces apoptosis through mitochondrial hyperpolarization and mtDNA damage in human hepatoma G2 cells. Free Radic. Biol. Med. 43, 968–975. 10.1016/j.freeradbiomed.2007.06.006 17697941

[B16] Chang M. Wu M. Li H. (2017). Curcumin combined with glycyrrhetinic acid inhibits the development of hepatocellular carcinoma cells by down-regulating the PTEN/PI3K/AKT signalling pathway. Am. J. Transl. Res. 9, 5567–5575. 29312508 PMC5752906

[B17] Chou T. C. (2011). The mass-action law based algorithm for cost-effective approach for cancer drug discovery and development. Am. J. Cancer Res. 1, 925–954. 22016837 PMC3196289

[B18] Dai X. Z. Yin H. T. Sun L. F. Hu X. Zhou C. Zhou Y. (2013). Potential therapeutic efficacy of curcumin in liver cancer. Asian pac. J. Cancer Prev. 14, 3855–3859. 10.7314/apjcp.2013.14.6.3855 23886196

[B19] den Hollander P. Savage M. I. Brown P. H. (2013). Targeted therapy for breast cancer prevention. Front. Oncol. 3, 250. 10.3389/fonc.2013.00250 24069582 PMC3780469

[B20] Deng Y. I. Verron E. Rohanizadeh R. (2016). Molecular mechanisms of anti-metastatic activity of curcumin. Anticancer Res. 36, 5639–5647. 10.21873/anticanres.11147 27793885

[B21] Dera A. A. Rajagopalan P. Al Fayi M. Ahmed I. Chandramoorthy H. C. (2020). Indirubin-3-monoxime and thymoquinone exhibit synergistic efficacy as therapeutic combination in *in-vitro* and *in-vivo* models of lung cancer. Arch. Pharm. Res. 43, 655–665. 10.1007/s12272-020-01241-2 32588331

[B22] Dong Z. Feng Q. Zhang H. Liu Q. Gong J. (2021). Curcumin enhances drug sensitivity of gemcitabine-resistant lung cancer cells and inhibits metastasis. Pharmazie 76, 538–543. 10.1691/PH.2021.0927 34782038

[B23] Duan W. Chang Y. Li R. Xu Q. Lei J. Yin C. (2014). Curcumin inhibits hypoxia inducible factor‑1α‑induced epithelial‑mesenchymal transition in HepG2 hepatocellular carcinoma cells. Mol. Med. Rep. 10, 2505–2510. 10.3892/mmr.2014.2551 25216080

[B24] El-Far A. H. Al Jaouni S. K. Li W. Mousa S. A. (2018b). Protective roles of thymoquinone nanoformulations: Potential nanonutraceuticals in human diseases. Nutrients 10, 1369. 10.3390/nu10101369 PMC6213571 30257423

[B25] El-Far A. H. Darwish N. H. E. Mousa S. A. (2020a). Senescent colon and breast cancer cells induced by doxorubicin exhibit enhanced sensitivity to curcumin, caffeine, and thymoquinone. Integr. Cancer Ther. 19, 1534735419901160. 10.1177/1534735419901160 32054357 PMC7025418

[B26] El-Far A. H. Godugu K. Noreldin A. E. Saddiq A. A. Almaghrabi O. A. Al Jaouni S. K. (2021a). Thymoquinone and costunolide induce apoptosis of both proliferative and doxorubicin-induced-senescent colon and breast cancer cells. Integr. Cancer Ther. 20, 15347354211035450. 10.1177/15347354211035450 34490824 PMC8427913

[B27] El-Far A. H. Tantawy M. A. Al Jaouni S. K. Mousa S. A. (2020b). Thymoquinone-chemotherapeutic combinations: new regimen to combat cancer and cancer stem cells. Naunyn. Schmiedeb. Arch. Pharmacol. 393, 1581–1598. 10.1007/s00210-020-01898-y 32458010

[B28] El-Far A. H. (2015). Thymoquinone anticancer discovery: possible mechanisms. Curr. Drug Discov. Technol. 12, 80–89. 10.2174/1570163812666150716111821 26264075

[B29] El-Far A. H. A. M. Munesue S. Harashima A. Sato A. Shindo M. Nakajima S. (2018a). *In vitro* anticancer effects of a RAGE inhibitor discovered using a structure-based drug design system. Oncol. Lett. 15, 4627–4634. 10.3892/ol.2018.7902 29541234 PMC5835888

[B30] El-Far A. H. Salaheldin T. A. Godugu K. Darwish N. H. Mousa S. A. (2021b). Thymoquinone and its nanoformulation attenuate colorectal and breast cancers and alleviate doxorubicin-induced cardiotoxicity. Nanomedicine 16, 1457–1469. 10.2217/nnm-2021-0103 34132104

[B31] ElKhoely A. Hafez H. F. Ashmawy A. M. Badary O. Abdelaziz A. Mostafa A. (2015). Chemopreventive and therapeutic potentials of thymoquinone in HepG2 cells: mechanistic perspectives. J. Nat. Med. 69, 313–323. 10.1007/s11418-015-0895-7 25796541

[B32] Endo H. Inoue I. Masunaka K. Tanaka M. Yano M. (2020). Curcumin induces apoptosis in lung cancer cells by 14-3-3 protein-mediated activation of bad. Biosci. Biotechnol. Biochem. 84, 2440–2447. 10.1080/09168451.2020.1808443 32841581

[B33] Fahmy H. M. (2019). *In vitro* study of the cytotoxicity of thymoquinone/curcumin fluorescent liposomes. Naunyn. Schmiedeb. Arch. Pharmacol. 392, 1465–1476. 10.1007/s00210-019-01688-1 31377882

[B34] Fan H. Tian W. Ma X. (2014). Curcumin induces apoptosis of HepG2 cells via inhibiting fatty acid synthase. Target. Oncol. 9, 279–286. 10.1007/s11523-013-0286-5 23821378

[B35] Glade M. J. (1999). Food, nutrition, and the prevention of cancer: a global perspective. American institute for cancer research/world cancer research fund, american institute for cancer research, 1997. Nutrition 15, 523–526. 10.1016/s0899-9007(99)00021-0 10378216

[B36] Gong Y. Firestone G. L. Bjeldanes L. F. (2006). 3, 3'-diindolylmethane is a novel topoisomerase IIalpha catalytic inhibitor that induces S-phase retardation and mitotic delay in human hepatoma HepG2 cells. Mol. Pharmacol. 69, 1320–1327. 10.1124/mol.105.018978 16385077

[B37] Guzmán C. Bagga M. Kaur A. Westermarck J. Abankwa D. (2014). Colonyarea: an Imagej plugin to automatically quantify colony formation in clonogenic assays. PLoS One 9, e92444. 10.1371/JOURNAL.PONE.0092444 24647355 PMC3960247

[B38] Han L. Wang Y. Sun S. (2020). Curcumin inhibits proliferation of hepatocellular carcinoma cells through down regulation of DJ-1. Cancer Biomark. 29, 1–8. 10.3233/CBM-190427 32417759 PMC12662505

[B39] Hassan N. M. Alhossary A. A. Mu Y. Kwoh C. K. (2017). Protein-ligand blind docking using quickvina-w with inter-process spatio-temporal integration. Sci. Rep. 7, 15451. 10.1038/s41598-017-15571-7 29133831 PMC5684369

[B40] Herrmann A. Moss D. Sée V. (2016). The chorioallantoic membrane of the chick embryo to assess tumor formation and metastasis. Methods Mol. Biol. 1464, 97–105. 10.1007/978-1-4939-3999-2_9 27858359

[B41] Jiang Y. Fang Y. Ye Y. Xu X. Wang B. Gu J. (2019). Anti-cancer effects of 3, 3'-diindolylmethane on human hepatocellular carcinoma cells is enhanced by calcium ionophore: the role of cytosolic ca^2+^ and p38 mapK. Front. Pharmacol. 10, 1167. 10.3389/fphar.2019.01167 31649538 PMC6795059

[B42] Jiao D. Wang J. Lu W. Tang X. Chen J. Mou H. (2016). Curcumin inhibited HGF-induced EMT and angiogenesis through regulating c-Met dependent PI3K/Akt/mTOR signaling pathways in lung cancer. Mol. Ther. oncolytics 3, 16018. 10.1038/mto.2016.18 27525306 PMC4972091

[B43] Joshi P. Joshi S. Semwal D. Bisht A. Paliwal S. Dwivedi J. (2021). Curcumin: an insight into molecular pathways involved in anticancer activity. Mini Rev. Med. Chem. 21, 2420–2457. 10.2174/1389557521666210122153823 33480345

[B44] Khan A. Alsahli M. A. Aljasir M. A. Maswadeh H. Mobark M. A. Azam F. (2022). Safety, stability, and therapeutic efficacy of long-circulating tq-incorporated liposomes: implication in the treatment of lung cancer. Pharmaceutics 14, 153. 10.3390/pharmaceutics14010153 35057049 PMC8778344

[B45] Klingenberg M. Becker J. Eberth S. Kube D. Wilting J. (2014). The chick chorioallantoic membrane as an *in vivo* xenograft model for Burkitt lymphoma. BMC Cancer 14, 339. 10.1186/1471-2407-14-339 24884418 PMC4036709

[B46] Koehn F. E. Carter G. T. (2005). The evolving role of natural products in drug discovery. Nat. Rev. Drug Discov. 4, 206–220. 10.1038/nrd1657 15729362

[B47] Kuppusamy P. Yusoff M. M. Maniam G. P. Ichwan S. J. Soundharrajan I. Govindan N. (2014). Nutraceuticals as potential therapeutic agents for colon cancer: a review. Acta Pharm. Sin. B 4, 173–181. 10.1016/j.apsb.2014.04.002 26579381 PMC4629076

[B48] Lee W.-H. Loo C.-Y. Traini D. Young P. M. (2020). Development and evaluation of paclitaxel and curcumin dry powder for inhalation lung cancer treatment. Pharmaceutics 13, 9. 10.3390/pharmaceutics13010009 PMC7822152 33375181

[B49] Li X. Ma S. Yang P. Sun B. Zhang Y. Sun Y. (2018). Anticancer effects of curcumin on nude mice bearing lung cancer A549 cell subsets SP and NSP cells. Oncol. Lett. 16, 6756–6762. 10.3892/ol.2018.9488 30405819 PMC6202465

[B50] Li Y. Li S. Meng X. Gan R. Y. Zhang J. J. Li H. B. (2017). Dietary natural products for prevention and treatment of breast cancer. Nutrients 9, 728. 10.3390/nu9070728 PMC5537842 28698459

[B51] Liang W. F. Gong Y. X. Li H. F. Sun F. L. Li W. L. Chen D. Q. (2021). Curcumin activates ros signaling to promote pyroptosis in hepatocellular carcinoma HepG2 cells. Vivo 35, 249–257. 10.21873/invivo.12253 PMC7880758 33402471

[B52] Lin L. Yan L. Liu Y. Qu C. Ni J. Li H. (2020). The burden and trends of primary liver cancer caused by specific etiologies from 1990 to 2017 at the global, regional, national, age, and sex level results from the global burden of disease study 2017. Liver Cancer 9, 563–582. 10.1159/000508568 33083281 PMC7548973

[B53] Liu F. Gao S. Yang Y. Zhao X. Fan Y. Ma W. (2017). Curcumin induced autophagy anticancer effects on human lung adenocarcinoma cell line A549. Oncol. Lett. 14, 2775–2782. 10.3892/ol.2017.6565 28928819 PMC5588543

[B54] Molina J. R. Yang P. Cassivi S. D. Schild S. E. Adjei A. A. (2008). Non-small cell lung cancer: epidemiology, risk factors, treatment, and survivorship. Mayo Clin. Proc. 83, 584–594. 10.4065/83.5.584 18452692 PMC2718421

[B55] Mousa D. S. El-Far A. H. Saddiq A. A. Sudha T. Mousa S. A. (2020). Nanoformulated bioactive compounds derived from different natural products combat pancreatic cancer cell proliferation. Int. J. Nanomedicine 15, 2259–2268. 10.2147/IJN.S238256 32280218 PMC7127850

[B56] Munakarmi S. Shrestha J. Shin H. B. Lee G. H. Jeong Y. J. (2021). 3,3'-diindolylmethane suppresses the growth of hepatocellular carcinoma by regulating its invasion, migration, and er stress-mediated mitochondrial apoptosis. Cells 10, 1178. 10.3390/cells10051178 34066056 PMC8151225

[B57] Ndreshkjana B. Çapci A. Klein V. Chanvorachote P. Muenzner J. K. Huebner K. (2019). Combination of 5-fluorouracil and thymoquinone targets stem cell gene signature in colorectal cancer cells. Cell Death Dis. 10, 379. 10.1038/s41419-019-1611-4 31097715 PMC6522523

[B58] Park S. Y. Matte A. Jung Y. Ryu J. Anand W. B. Han E. Y. (2020). Pathologic angiogenesis in the bone marrow of humanized sickle cell mice is reversed by blood transfusion. Blood 135, 2071–2084. 10.1182/blood.2019002227 31990287 PMC7273832

[B59] Saaristo A. Karpanen T. Alitalo K. (2000). Mechanisms of angiogenesis and their use in the inhibition of tumor growth and metastasis. Oncogene 19, 6122–6129. 10.1038/sj.onc.1203969 11156525

[B60] Samarghandian S. Azimi-Nezhad M. Farkhondeh T. (2019). Thymoquinone-induced antitumor and apoptosis in human lung adenocarcinoma cells. J. Cell. Physiol. 234, 10421–10431. 10.1002/jcp.27710 30387147

[B61] Shah D. Gandhi M. Kumar A. Cruz-Martins N. Sharma R. Nair S. (2021). Current insights into epigenetics, noncoding RNA interactome and clinical pharmacokinetics of dietary polyphenols in cancer chemoprevention. Crit. Rev. Food Sci. Nutr. 26, 1–37. 10.1080/10408398.2021.1968786 34433338

[B62] Shanmugam M. K. Ahn K. S. Hsu A. Woo C. C. Yuan Y. Tan K. H. B. (2018). Thymoquinone inhibits bone metastasis of breast cancer cells through abrogation of the CXCR4 signaling axis. Front. Pharmacol. 9, 1294. 10.3389/fphar.2018.01294 30564115 PMC6288203

[B63] Sharma R. Martins N. (2020). Telomeres, DNA damage and ageing: potential leads from ayurvedic rasayana (anti-ageing) drugs. J. Clin. Med. 9, 2544. 10.3390/jcm9082544 PMC7465058 32781627

[B64] Sudha T. El-Far A. H. Mousa D. S. Mousa S. A. (2020). Resveratrol and its nanoformulation attenuate growth and the angiogenesis of xenograft and orthotopic colon cancer models. Molecules 25, 1412. 10.3390/molecules25061412 PMC7144556 32244860

[B65] Sung H. Ferlay J. Siegel R. L. Laversanne M. Soerjomataram I. Jemal A. (2021). Global cancer statistics 2020: GLOBOCAN estimates of incidence and mortality worldwide for 36 cancers in 185 countries. Ca. Cancer J. Clin. 71, 209–249. 10.3322/caac.21660 33538338

[B66] Thai A. A. Solomon B. J. Sequist L. V. Gainor J. F. Heist R. S. (2021). Lung cancer. Lancet 398, 535–554. 10.1016/S0140-6736(21)00312-3 34273294

[B67] Tian S. Liao L. Zhou Q. Huang X. Zheng P. Guo Y. (2021). Curcumin inhibits the growth of liver cancer by impairing myeloid-derived suppressor cells in murine tumor tissues. Oncol. Lett. 21, 286. 10.3892/ol.2021.12547 33732362 PMC7905673

[B68] Upadhyay P. Sarker S. Ghosh A. Gupta P. Das S. Ahir M. (2019). Transferrin-decorated thymoquinone-loaded PEG-PLGA nanoparticles exhibit anticarcinogenic effect in non-small cell lung carcinoma via the modulation of miR-34a and miR-16. Biomater. Sci. 7, 4325–4344. 10.1039/c9bm00912d 31411213

[B69] USDA Plants Database (2022a). Curcuma longa L. common turmeric. Available at: https://plants.sc.egov.usda.gov/home/plantProfile?symbol=CULO (Accessed June 8, 2022).

[B70] USDA Plants Database (2022b). Nigella sativa L. black cumin. Available at: https://plants.sc.egov.usda.gov/home/plantProfile?symbol=NISA2 (Accessed June 8, 2022).

[B71] Wang F. He Z. Dai W. Li Q. Liu X. Zhang Z. (2015). The role of the vascular endothelial growth factor/vascular endothelial growth factor receptors axis mediated angiogenesis in curcumin-loaded nanostructured lipid carriers induced human HepG2 cells apoptosis. J. Cancer Res. Ther. 11, 597–605. 10.4103/0973-1482.159086 26458588

[B72] Wang L. Han L. Tao Z. Zhu Z. Han L. Yang Z. (2018a). The curcumin derivative WZ35 activates ROS-dependent JNK to suppress hepatocellular carcinoma metastasis. Food Funct. 9, 2970–2978. 10.1039/C8FO00314A 29766185

[B73] Wang N. Feng T. Liu X. Liu Q. (2020). Curcumin inhibits migration and invasion of non-small cell lung cancer cells through up-regulation of miR-206 and suppression of PI3K/AKT/mTOR signaling pathway. Acta Pharm. 70, 399–409. 10.2478/acph-2020-0029 32074070

[B74] Wang W. H. Chen J. Zhang B. R. Lu S. J. Wang F. Peng L. (2018b). Curcumin inhibits proliferation and enhances apoptosis in A549 cells by downregulating lncRNA UCA1. Pharmazie 73, 402–407. 10.1691/PH.2018.8402 30001775

[B75] Wong S. C. Kamarudin M. N. A. Naidu R. (2021). Anticancer mechanism of curcumin on human glioblastoma. Nutrients 13, 950. 10.3390/nu13030950 33809462 PMC7998496

[B76] Yang J. Kuang X. R. Lv P. T. Yan X. X. (2015). Thymoquinone inhibits proliferation and invasion of human nonsmall-cell lung cancer cells via ERK pathway. Tumour Biol. 36, 259–269. 10.1007/s13277-014-2628-z 25238880

[B77] Yao Q. Lin M. Wang Y. Lai Y. Hu J. Fu T. (2015). Curcumin induces the apoptosis of A549 cells via oxidative stress and MAPK signaling pathways. Int. J. Mol. Med. 36, 1118–1126. 10.3892/ijmm.2015.2327 26310655

[B78] Yoysungnoen P. Wirachwong P. Bhattarakosol P. Niimi H. Patumraj S. (2006). Effects of curcumin on tumor angiogenesis and biomarkers, COX-2 and VEGF, in hepatocellular carcinoma cell-implanted nude mice. Clin. Hemorheol. Microcirc. 34, 109–115. 16543625

[B79] Yoysungnoen P. Wirachwong P. Changtam C. Suksamrarn A. Patumraj S. (2008). Anti-cancer and anti-angiogenic effects of curcumin and tetrahydrocurcumin on implanted hepatocellular carcinoma in nude mice. World J. Gastroenterol. 14, 2003–2009. 10.3748/WJG.14.2003 18395899 PMC2701520

[B80] Zhang W. W. Feng Z. Narod S. A. (2014). Multiple therapeutic and preventive effects of 3, 3'-diindolylmethane on cancers including prostate cancer and high grade prostatic intraepithelial neoplasia. J. Biomed. Res. 28, 339–348. 10.7555/JBR.28.20140008 25332705 PMC4197384

[B81] Zhao R. Tin L. Zhang Y. Wu Y. Jin Y. Jin X. (2016). EF24 suppresses invasion and migration of hepatocellular carcinoma cells *in vitro* via inhibiting the phosphorylation of src. Biomed. Res. Int. 2016, 8569684. 10.1155/2016/8569684 27999817 PMC5141541

