# Supplementary material for: Curcumin, thymoquinone, and 3, 3′-diindolylmethane combinations attenuate lung and liver cancers progression
Source: Front Pharmacol. 2022 Jun 29;13:936996. doi: 10.3389/fphar.2022.936996 (PMC9277483; doi:10.3389/fphar.2022.936996)
Supplement: Supplementary file 1 [file DataSheet2.PDF]

ClinicalTrials.gov Search Results 05/03/2022

	NCT Number	Title	Other Names	Status	Conditions	Interventions	Characteristics	Population	Sponsor/ Collaborators	Funder Type	Dates	Locations
1	NCT03208790	Clinical and Immunohisochemical Evaluation of Chemopreventive Effect of Thymoquinone on Oral Potentially Malignant Lesions.<br>Study Documents:	Title Acronym:<br>Other Ids: TQ-OPML	Completed	• Premalignant Lesion	• Drug: Nigella sativa buccal tablets 10mg<br>• Drug: Nigella sativa buccal tablets 5mg<br>• Drug: Placebo buccal tablets	Study Type: Interventional<br>Phase: Phase 2<br>Study Design:<br>• Allocation: Randomized<br>• Intervention Model: Parallel Assignment<br>• Masking: Triple (Participant, Investigator, Outcomes Assessor)<br>• Primary Purpose: Treatment<br>Outcome Measures:<br>• clinical response<br>• Molecular evidence of malignant transformation	Enrollment: 48<br>Age: 18 Years to 75 Years (Adult, Older Adult)<br>Sex: All	• Cairo University	• Other	Study Start: September 30, 2017<br>Primary Completion: March 12, 2020<br>Study Completion: March 12, 2020<br>First Posted: July 6, 2017<br>Results First Posted: No Results Posted<br>Last Update Posted: April 20, 2021	• Faculty of Dentistry, Cairo, Egypt

	NCT Number	Title	Other Names	Status	Conditions	Interventions	Characteristics	Population	Sponsor/ Collaborators	Funder Type	Dates	Locations
2	NCT04852510	Amelioration of Polycystic Ovary Syndrome Related Disorders by Supplementation of Thymoquinone and Metformin<br><br>Study Documents:	Title Acronym:<br><br>Other Ids: SGHM-JAN2019-OBGYN	Completed	•Polycystic Ovary Syndrome (PCOS)	•Drug: Metformin Versus a combination of Metformin and Thymoquinone (TQ)	Study Type: Interventional<br><br>Phase:<br>•Phase 2<br>•Phase 3<br><br>Study Design:<br>•Allocation: Randomized<br>•Intervention Model: Parallel Assignment<br>•Masking: None (Open Label)<br>•Primary Purpose: Treatment<br><br>Outcome Measures:<br>•The change of menstrual cycle pattern.<br>•The change of body mass index (BMI).<br>•Change of body fat distribution as proved by change of Waist/Hip ratio.<br>•Change of oral glucose tolerance test (OGTT) results.<br>•Change of Glycosylated Hemoglobin A1C levels.<br>•Change of serum Malondialdehyde (MDA) concentrations.<br>•Change of serum superoxide dismutase (SOD) activity.	Enrollment: 253<br><br>Age: 18 Years to 35 Years (Adult)<br><br>Sex: Female	•Saudi German Hospital - Madinah	•Other	Study Start: February 1, 2019<br><br>Primary Completion: August 31, 2020<br><br>Study Completion: August 31, 2020<br><br>First Posted: April 21, 2021<br><br>Results First Posted: No Results Posted<br><br>Last Update Posted: April 21, 2021	•Saudi German Hospital, Al Mad#nah, Madinah, Saudi Arabia
