# Supplementary material for: Curcumin, thymoquinone, and 3, 3′-diindolylmethane combinations attenuate lung and liver cancers progression
Source: Front Pharmacol. 2022 Jun 29;13:936996. doi: 10.3389/fphar.2022.936996 (PMC9277483; doi:10.3389/fphar.2022.936996)
Supplement: Supplementary file 3 [file DataSheet7.docx]

**Data Sheet 7.** Interference network of curcumin (Cur), thymoquinone (TQ), and 3, 3'-diindolylmethane (DIM) against lung and liver cancers extracted from the previous studies in comparative toxicogenomics database (<http://ctdbase.org/>)

|  | **Liver cancer** | | | **Lung cancer** | | |
| --- | --- | --- | --- | --- | --- | --- |
| **Inference Network** | **Cur** | **TQ** | **DIM** | **Cur** | **TQ** | **DIM** |
| ABCB1B |  |  |  |  |  |  |
| ACACA |  |  |  |  |  |  |
| ACE |  |  |  |  |  |  |
| ACLY |  |  |  |  |  |  |
| ACO2 |  |  |  |  |  |  |
| ALB |  |  |  |  |  |  |
| ALDOA |  |  |  |  |  |  |
| ALOX5 |  |  |  |  |  |  |
| APC |  |  |  |  |  |  |
| APOE |  |  |  |  |  |  |
| ATM |  |  |  |  |  |  |
| AURKA |  |  |  |  |  |  |
| AURKB |  |  |  |  |  |  |
| AXIN1 |  |  |  |  |  |  |
| BAK1 |  |  |  |  |  |  |
| CAPG |  |  |  |  |  |  |
| CBR1 |  |  |  |  |  |  |
| CCL3 |  |  |  |  |  |  |
| CCN2 |  |  |  |  |  |  |
| CDC20 |  |  |  |  |  |  |
| CDK1 |  |  |  |  |  |  |
| CDKN2A |  |  |  |  |  |  |
| CEBPA |  |  |  |  |  |  |
| CEBPB |  |  |  |  |  |  |
| CES1 |  |  |  |  |  |  |
| CHEK2 |  |  |  |  |  |  |
| CKB |  |  |  |  |  |  |
| COL4A2 |  |  |  |  |  |  |
| CREBBP |  |  |  |  |  |  |
| CRP |  |  |  |  |  |  |
| CTNNB1 |  |  |  |  |  |  |
| CXCL1 |  |  |  |  |  |  |
| CXCL12 |  |  |  |  |  |  |
| CXCL14 |  |  |  |  |  |  |
| CXCL8 |  |  |  |  |  |  |
| CYP2E1 |  |  |  |  |  |  |
| CYP24A1 |  |  |  |  |  |  |
| CYP2E1 |  |  |  |  |  |  |
| DNMT3A |  |  |  |  |  |  |
| DCN |  |  |  |  |  |  |
| E2F1 |  |  |  |  |  |  |
| E2F5 |  |  |  |  |  |  |
| EGF |  |  |  |  |  |  |
| EGFR |  |  |  |  |  |  |
| EGR1 |  |  |  |  |  |  |
| ENO1 |  |  |  |  |  |  |
| EP300 |  |  |  |  |  |  |
| ERBB3 |  |  |  |  |  |  |
| ERCC1 |  |  |  |  |  |  |
| F2 |  |  |  |  |  |  |
| FANCD2 |  |  |  |  |  |  |
| FANCI |  |  |  |  |  |  |
| FAS |  |  |  |  |  |  |
| FASLG |  |  |  |  |  |  |
| FASN |  |  |  |  |  |  |
| FDFT1 |  |  |  |  |  |  |
| FGFR2 |  |  |  |  |  |  |
| FOSL2 |  |  |  |  |  |  |
| FOXA1 |  |  |  |  |  |  |
| GCLC |  |  |  |  |  |  |
| GJA1 |  |  |  |  |  |  |
| GLI1 |  |  |  |  |  |  |
| GPER1 |  |  |  |  |  |  |
| GSTP1 |  |  |  |  |  |  |
| H19 |  |  |  |  |  |  |
| HAMP |  |  |  |  |  |  |
| HEBP2 |  |  |  |  |  |  |
| HES1 |  |  |  |  |  |  |
| HGF |  |  |  |  |  |  |
| HMGCR |  |  |  |  |  |  |
| HNRNPA1 |  |  |  |  |  |  |
| ID2 |  |  |  |  |  |  |
| ID3 |  |  |  |  |  |  |
| IFNA1 |  |  |  |  |  |  |
| IGF1 |  |  |  |  |  |  |
| IGF1R |  |  |  |  |  |  |
| IGF2 |  |  |  |  |  |  |
| IKBKG |  |  |  |  |  |  |
| IL12A |  |  |  |  |  |  |
| IL12B |  |  |  |  |  |  |
| IRS1 |  |  |  |  |  |  |
| IRS2 |  |  |  |  |  |  |
| ITGB1 |  |  |  |  |  |  |
| JUN |  |  |  |  |  |  |
| JUNB |  |  |  |  |  |  |
| JUND |  |  |  |  |  |  |
| KEAP1 |  |  |  |  |  |  |
| KIT |  |  |  |  |  |  |
| KRAS |  |  |  |  |  |  |
| MAP2K1 |  |  |  |  |  |  |
| MAPK14 |  |  |  |  |  |  |
| MAPT |  |  |  |  |  |  |
| MCL1 |  |  |  |  |  |  |
| MCM2 |  |  |  |  |  |  |
| MECP2 |  |  |  |  |  |  |
| MIR146A |  |  |  |  |  |  |
| MIR21 |  |  |  |  |  |  |
| MIR222 |  |  |  |  |  |  |
| MIR30A |  |  |  |  |  |  |
| MIR34A |  |  |  |  |  |  |
| MMP1 |  |  |  |  |  |  |
| MMP10 |  |  |  |  |  |  |
| MMP14 |  |  |  |  |  |  |
| MS4A1 |  |  |  |  |  |  |
| MT1A |  |  |  |  |  |  |
| MT2A |  |  |  |  |  |  |
| MVK |  |  |  |  |  |  |
| NFKBIA |  |  |  |  |  |  |
| NFYA |  |  |  |  |  |  |
| NPPA |  |  |  |  |  |  |
| OAT |  |  |  |  |  |  |
| OGG1 |  |  |  |  |  |  |
| OSMR |  |  |  |  |  |  |
| PCK1 |  |  |  |  |  |  |
| PDGFB |  |  |  |  |  |  |
| PDIA3 |  |  |  |  |  |  |
| PHB1 |  |  |  |  |  |  |
| PIK3CA |  |  |  |  |  |  |
| PLK1 |  |  |  |  |  |  |
| PRDX6 |  |  |  |  |  |  |
| PRKCE |  |  |  |  |  |  |
| PRKN |  |  |  |  |  |  |
| PTEN |  |  |  |  |  |  |
| PTHLH |  |  |  |  |  |  |
| PTK2 |  |  |  |  |  |  |
| PTPRO |  |  |  |  |  |  |
| RAC1 |  |  |  |  |  |  |
| RAF1 |  |  |  |  |  |  |
| RARA |  |  |  |  |  |  |
| RB1 |  |  |  |  |  |  |
| RIMS2 |  |  |  |  |  |  |
| SCD |  |  |  |  |  |  |
| SERPINE1 |  |  |  |  |  |  |
| SFXN1 |  |  |  |  |  |  |
| SHH |  |  |  |  |  |  |
| SLC11A2 |  |  |  |  |  |  |
| SLC2A1 |  |  |  |  |  |  |
| SLC2A2 |  |  |  |  |  |  |
| SLC3A2 |  |  |  |  |  |  |
| SOCS3 |  |  |  |  |  |  |
| SOX2 |  |  |  |  |  |  |
| SOX9 |  |  |  |  |  |  |
| SPP1 |  |  |  |  |  |  |
| SQLE |  |  |  |  |  |  |
| SREBF1 |  |  |  |  |  |  |
| SREBF2 |  |  |  |  |  |  |
| SRM |  |  |  |  |  |  |
| STAT1 |  |  |  |  |  |  |
| STAT4 |  |  |  |  |  |  |
| STAT5A |  |  |  |  |  |  |
| TERT |  |  |  |  |  |  |
| TFRC |  |  |  |  |  |  |
| TGFBR2 |  |  |  |  |  |  |
| TH |  |  |  |  |  |  |
| THY1 |  |  |  |  |  |  |
| TNFSF10 |  |  |  |  |  |  |
| TRP53 |  |  |  |  |  |  |
| TRP53 |  |  |  |  |  |  |
| TSC2 |  |  |  |  |  |  |
| UBE2C |  |  |  |  |  |  |
| VHL |  |  |  |  |  |  |
| VCAM1 |  |  |  |  |  |  |
| WT1 |  |  |  |  |  |  |
| XRCC5 |  |  |  |  |  |  |
| ABCB1 |  |  |  |  |  |  |
| AHR |  |  |  |  |  |  |
| AR |  |  |  |  |  |  |
| ATF3 |  |  |  |  |  |  |
| BBC3 |  |  |  |  |  |  |
| CDC25C |  |  |  |  |  |  |
| CDKN1B |  |  |  |  |  |  |
| CTSD |  |  |  |  |  |  |
| CYP1A1 |  |  |  |  |  |  |
| CYP1A2 |  |  |  |  |  |  |
| CYP1B1 |  |  |  |  |  |  |
| ERBB2 |  |  |  |  |  |  |
| ESR1 |  |  |  |  |  |  |
| HSPB1 |  |  |  |  |  |  |
| IL2 |  |  |  |  |  |  |
| KDR |  |  |  |  |  |  |
| NME1 |  |  |  |  |  |  |
| NOS2 |  |  |  |  |  |  |
| NQO1 |  |  |  |  |  |  |
| PGD |  |  |  |  |  |  |
| PIK3CA |  |  |  |  |  |  |
| PTGS2 |  |  |  |  |  |  |
| RPS6 |  |  |  |  |  |  |
| TALDO1 |  |  |  |  |  |  |
| TF |  |  |  |  |  |  |
| TNFSF10 |  |  |  |  |  |  |
| TYMS |  |  |  |  |  |  |
| XRCC6 |  |  |  |  |  |  |
| AKT1 |  |  |  |  |  |  |
| BID |  |  |  |  |  |  |
| CASP8 |  |  |  |  |  |  |
| CAT |  |  |  |  |  |  |
| CCNE1 |  |  |  |  |  |  |
| CDKN1A |  |  |  |  |  |  |
| CTNNB1 |  |  |  |  |  |  |
| FOS |  |  |  |  |  |  |
| GPX1 |  |  |  |  |  |  |
| JAK2 |  |  |  |  |  |  |
| HIF1A |  |  |  |  |  |  |
| IL10 |  |  |  |  |  |  |
| IL1B |  |  |  |  |  |  |
| MIR125A |  |  |  |  |  |  |
| MKI67 |  |  |  |  |  |  |
| MPO |  |  |  |  |  |  |
| MTOR |  |  |  |  |  |  |
| PCNA |  |  |  |  |  |  |
| PPARG |  |  |  |  |  |  |
| SOD1 |  |  |  |  |  |  |
| SOD2 |  |  |  |  |  |  |
| STAT3 |  |  |  |  |  |  |
| STK11 |  |  |  |  |  |  |
| TGFB1 |  |  |  |  |  |  |
| TLR4 |  |  |  |  |  |  |
| TNF |  |  |  |  |  |  |
| TNFRSF10B |  |  |  |  |  |  |
| TOP2A |  |  |  |  |  |  |
| VEGFA |  |  |  |  |  |  |
| BCL2 |  |  |  |  |  |  |
| BCL2L1 |  |  |  |  |  |  |
| BIRC5 |  |  |  |  |  |  |
| CCNB1 |  |  |  |  |  |  |
| CCND1 |  |  |  |  |  |  |
| CDH1 |  |  |  |  |  |  |
| CDK4 |  |  |  |  |  |  |
| CDKN1A |  |  |  |  |  |  |
| CDKN1B |  |  |  |  |  |  |
| HMOX1 |  |  |  |  |  |  |
| HSPA5 |  |  |  |  |  |  |
| IFNG |  |  |  |  |  |  |
| IL6 |  |  |  |  |  |  |
| MAPK1 |  |  |  |  |  |  |
| MAPK3 |  |  |  |  |  |  |
| MDM2 |  |  |  |  |  |  |
| MMP2  1CK7 |  |  |  |  |  |  |
| MMP9  1GKC |  |  |  |  |  |  |
| MYC |  |  |  |  |  |  |
| NFE2L2 |  |  |  |  |  |  |
| PARP1 |  |  |  |  |  |  |
| TNF |  |  |  |  |  |  |
| TP53 |  |  |  |  |  |  |
| CCN1 |  |  |  |  |  |  |
| CCR4 |  |  |  |  |  |  |
| CYP17A1 |  |  |  |  |  |  |
| GDF15 |  |  |  |  |  |  |
| GSTM1 |  |  |  |  |  |  |
| KRT18 |  |  |  |  |  |  |
| TFPI2 |  |  |  |  |  |  |
| TYMS |  |  |  |  |  |  |
| AFP |  |  |  |  |  |  |
| ATG7 |  |  |  |  |  |  |
| EPHX1 |  |  |  |  |  |  |
| IL6R |  |  |  |  |  |  |
| MET |  |  |  |  |  |  |
| STK11 |  |  |  |  |  |  |
