# Supplementary material for: Curcumin, thymoquinone, and 3, 3′-diindolylmethane combinations attenuate lung and liver cancers progression
Source: Front Pharmacol. 2022 Jun 29;13:936996. doi: 10.3389/fphar.2022.936996 (PMC9277483; doi:10.3389/fphar.2022.936996)
Supplement: Supplementary file 5 [file DataSheet3.PDF]

ClinicalTrials.gov Search Results 05/03/2022

	NCT Number	Title	Other Names	Status	Conditions	Interventions	Characteristics	Population	Sponsor/ Collaborators	Funder Type	Dates	Locations
1	NCT00888654	Diindolylmethane in Treating Patients With Stage I or Stage II Prostate Cancer Undergoing Radical Prostatectomy<br>Study Documents:	Title Acronym:<br>Other Ids:<br>• CDR0000641168<br>• P30CA022453<br>• WSU-2007-128	Completed	• Prostate Cancer	• Drug: B-Dim<br>• Procedure: Radical Prosatectomy	Study Type: Interventional<br>Phase: Phase 2<br>Study Design:<br>• Allocation: N/A<br>• Intervention Model: Single Group Assignment<br>• Masking: None (Open Label)<br>• Primary Purpose: Treatment<br>Outcome Measures:<br>• Mean Level of Diindolylmethane in Prostate Tissue After Treatment<br>• Serum Levels of PSA, Testosterone, and Diindolylmethane<br>• Levels of Androgen Receptor in Prostate Tissue	Enrollment: 41<br>Age: 18 Years to 120 Years (Adult, Older Adult)<br>Sex: Male	• Barbara Ann Karmanos Cancer Institute<br>• National Cancer Institute (NCI)	• Other<br>• NIH	Study Start: August 2009<br>Primary Completion: April 2014<br>Study Completion: April 2014<br>First Posted: April 27, 2009<br>Results First Posted: March 30, 2015<br>Last Update Posted: March 19, 2018	• Barbara Ann Karmanos Cancer Institute, Detroit, Michigan, United States<br>• Josephine Ford Cancer Center at Henry Ford Hospital, Detroit, Michigan, United States
2	NCT01612910	Oral Microencapsulated Diindolylmethane in Treating Patients With Stage II-III Triple Negative, Androgen Receptor Positive Breast Cancer Who Have Undergone Chemotherapy and Surgery<br>Study Documents:	Title Acronym:<br>Other Ids:<br>2011-111	Withdrawn	• Breast Cancer Female	• Drug: oral microencapsulated diindolylmethane<br>• Other: laboratory biomarker analysis	Study Type: Interventional<br>Phase: Phase 2<br>Study Design:<br>• Allocation: N/A<br>• Intervention Model: Single Group Assignment<br>• Masking: None (Open Label)<br>• Primary Purpose: Treatment<br>Outcome Measures:<br>• Progression Free Survival (PFS), defined as clear development of new sites of disease, measurable or non-measurable or death<br>• Association of serum BR-DIM levels and changes in correlative biomarkers with time to progression	Enrollment: 0<br>Age: 18 Years and older (Adult, Older Adult)<br>Sex: Female	• Barbara Ann Karmanos Cancer Institute<br>• National Cancer Institute (NCI)	• Other<br>• NIH	Study Start: June 2012<br>Primary Completion: February 2014<br>Study Completion: February 2014<br>First Posted: June 6, 2012<br>Results First Posted: No Results Posted<br>Last Update Posted: February 12, 2014	

	NCT Number	Title	Other Names	Status	Conditions	Interventions	Characteristics	Population	Sponsor/ Collaborators	Funder Type	Dates	Locations
3	NCT01391689	Diindolylmethane in Treating Patients With Breast Cancer<br>Study Documents:	Title Acronym:<br>Other Ids:<br>• 10-0366-04<br>• NCI-2011-00710<br>• R01CA149417-01A	Completed	• Stage IA Breast Cancer<br>• Stage IB Breast Cancer<br>• Stage II Breast Cancer<br>• Stage IIIA Breast Cancer	• Dietary Supplement: diindolylmethane<br>• Dietary Supplement: placebo	Study Type: Interventional<br>Phase: Not Applicable<br>Study Design:<br>• Allocation: Randomized<br>• Intervention Model: Parallel Assignment<br>• Masking: Double (Participant, Investigator)<br>• Primary Purpose: Treatment<br>Outcome Measures:<br>• Urinary 2OHE1:16alpha OHE1 ratio<br>• Plasma TAM metabolites (ng/mL)<br>• Serum Estrogen (estradiol) (pg/mL)<br>• Self reported vaginal bleeding<br>• mammographic density	Enrollment: 144<br>Age: 19 Years and older (Adult, Older Adult)<br>Sex: Female	• University of Arizona<br>• National Cancer Institute (NCI)	• Other<br>• NIH	Study Start: February 2011<br>Primary Completion: September 2014<br>Study Completion: July 31, 2016<br>First Posted: July 12, 2011<br>Results First Posted: No Results Posted<br>Last Update Posted: November 6, 2017	• Arizona Cancer Center, Tucson, Arizona, United States

	NCT Number	Title	Other Names	Status	Conditions	Interventions	Characteristics	Population	Sponsor/ Collaborators	Funder Type	Dates	Locations
4	NCT00305747	Diindolylmethane in Treating Patients With Nonmetastatic Prostate Cancer That Has Not Responded To Previous Hormone Therapy<br><br>Study Documents:	Title Acronym:<br><br>Other Ids:<br>• CDR0000462637<br>• P30CA022453<br>• WSU-D-2979<br>• WSU-0507002581	Completed	• Prostate Cancer	• Drug: BR-DIM	Study Type:<br>Interventional<br><br>Phase:<br>Phase 1<br><br>Study Design:<br>• Allocation: N/A<br>• Intervention Model: Single Group Assignment<br>• Masking: None (Open Label)<br>• Primary Purpose: Treatment<br><br>Outcome Measures:<br>• Maximum tolerated dose (MTD), Dose limiting toxicity (DLT) & toxicities during study and for 30 days after<br>• Plasma pharmacokinetics as measured by occurrences of toxicity<br>• Serum prostate specific antigen as measured by complete plasma concentration-time profile<br>• Correlate changes in expression levels of NF-kB lymphocytes in with serum prostate specific antigen levels by serum prostate specific antigen level<br>• Quality of life (QOL) by Life Orient. Test-Rev., Duke-UNC Func. Social Support Questionnaire, EORTC QOL questionnaire, QLQ-PR25 questionnaire, and the Hosp. Anxiety & Depression Scale	Enrollment:<br>12<br><br>Age:<br>18 Years and older (Adult, Older Adult)<br><br>Sex:<br>Male	• Barbara Ann Karmanos Cancer Institute<br>• National Cancer Institute (NCI)	• Other<br>• NIH	Study Start:<br>August 2005<br><br>Primary Completion:<br>September 2010<br><br>Study Completion:<br>September 2010<br><br>First Posted:<br>March 22, 2006<br><br>Results First Posted:<br>No Results Posted<br><br>Last Update Posted:<br>January 15, 2014	• Barbara Ann Karmanos Cancer Institute, Detroit, Michigan, United States<br>• Weisberg Cancer Treatment Center, Detroit, Michigan, United States

	NCT Number	Title	Other Names	Status	Conditions	Interventions	Characteristics	Population	Sponsor/ Collaborators	Funder Type	Dates	Locations
5	NCT00450229	Diindolylmethane in Treating Patients Undergoing Surgery for Stage I or Stage II Prostate Cancer<br><br>Study Documents:	Title Acronym:<br><br>Other Ids:<br>• NCI-2009-00905<br>• CO05816<br>• CDR0000656281<br>• H2006-0255<br>• N01CN35153	Completed	• Adenocarcinoma of the Prostate<br>• Prostate Cancer<br>• Stage I Prostate Cancer<br>• Stage II Prostate Cancer	• Drug: diindolylmethane<br>• Drug: placebo<br>• Procedure: therapeutic conventional surgery<br>• Other: laboratory biomarker analysis<br>• Other: pharmacological study	Study Type: Interventional<br><br>Phase: Phase 1<br><br>Study Design:<br>• Allocation: Randomized<br>• Intervention Model: Parallel Assignment<br>• Masking: Double (Participant, Investigator)<br>• Primary Purpose: Treatment<br><br>Outcome Measures:<br>• Tissue levels of DIM<br>• Urinary 2-hydroxyestrone/16-hydroxyestrone ratio<br>• Total PSA<br>• Serum testosterone<br>• IGF1:IGFBP-3 ratio<br>• Tissue measures of messenger RNA of CYPs (CYP1A2, CYP1A1, CYP2B1, CYP3A)<br>• DIM blood steady-state concentrations<br>• Measures of androgen receptor, PSA, Ki-67, caspase 3, and TUNEL	Enrollment: 45<br><br>Age: 18 Years and older (Adult, Older Adult)<br><br>Sex: Male	• National Cancer Institute (NCI)	• NIH	Study Start: February 2007<br><br>Primary Completion: February 2010<br><br>Study Completion: February 2010<br><br>First Posted: March 22, 2007<br><br>Results First Posted: No Results Posted<br><br>Last Update Posted: December 4, 2015	• University of Wisconsin Hospital and Clinics, Madison, Wisconsin, United States

	NCT Number	Title	Other Names	Status	Conditions	Interventions	Characteristics	Population	Sponsor/ Collaborators	Funder Type	Dates	Locations
6	NCT01022333	The Potential for Oral Diindolylmethane (DIM) Supplementation to Increase the Production of the BRCA1 Protein in BRCA1 Mutation Carriers<br><br>Study Documents:	Title Acronym:<br><br>Other Ids:<br>2009-015-B	Unknown status	• Breast Cancer	• Dietary Supplement: Diindolylmethane (DIM)	Study Type:<br>Interventional<br><br>Phase:<br>Phase 1<br><br>Study Design:<br>• Allocation: Non-Randomized<br>• Intervention Model: Parallel Assignment<br>• Masking: None (Open Label)<br>• Primary Purpose: Basic Science<br><br>Outcome Measures:<br>• Oral DIM supplementation increases BRCA1 mRNA expression and hence BRCA1 protein in women with a BRCA1 mutation<br>• Oral DIM supplementation causes favorable estrogen metabolism in women with a BRCA1 mutation	Enrollment:<br>300<br><br>Age:<br>25 Years to 45 Years (Adult)<br><br>Sex:<br>Female	• Women's College Hospital<br>• BioResponse	• Other<br>• Industry	Study Start:<br>July 2009<br><br>Primary Completion:<br>July 2010<br><br>Study Completion:<br>December 2010<br><br>First Posted:<br>December 1, 2009<br><br>Results First Posted:<br>No Results Posted<br><br>Last Update Posted:<br>December 1, 2009	• Familial Breast Cancer Research Unit, Women's College Research Institute, Toronto, Ontario, Canada

	NCT Number	Title	Other Names	Status	Conditions	Interventions	Characteristics	Population	Sponsor/ Collaborators	Funder Type	Dates	Locations
7	NCT00462813	Diindolylmethane in Treating Patients With Abnormal Cervical Cells<br><br>Study Documents:	Title Acronym:<br><br>Other Ids:<br>• CRUK-CRISP-1<br>• CDR0000539352<br>• ISRCTN47437431<br>• EU-20717	Completed	• Cervical Cancer<br>• Precancerous Condition	• Drug: oral microencapsulated diindolylmethane<br>• Genetic: polymerase chain reaction<br>• Other: cervical Papanicolaou test<br>• Other: cytology specimen collection procedure<br>• Procedure: colposcopic biopsy	Study Type: Interventional<br><br>Phase: Phase 3<br><br>Study Design:<br>• Allocation: Randomized<br>• Masking: Double<br>• Primary Purpose: Prevention<br><br>Outcome Measures:<br>• Biopsy confirmed high-grade cervical intraepithelial neoplasia (CIN) at 6 months<br>• Change in lesion size at 6 months<br>• Human papillomavirus (HPV) status and characteristics (type, viral load, and integration) at baseline and 6 months<br>• Change in cervical cytology at 6 months<br>• CIN # grade 3 on histology at 6 months<br>• Long term follow-up (i.e., 7 years)<br>• Migraine, premenstrual syndrome (PMS), menstruation, and body weight	Enrollment: 3000<br><br>Age: Child, Adult, Older Adult<br><br>Sex: Female	• Barts and the London School of Medicine and Dentistry<br>• National Cancer Institute (NCI)	• Other	Study Start: October 2004<br><br>Primary Completion: June 2008<br><br>Study Completion: January 2010<br><br>First Posted: April 19, 2007<br><br>Results First Posted: No Results Posted<br><br>Last Update Posted: August 7, 2013	• Barts and the London School of Medicine, London, England, United Kingdom<br>• University Hospital of Wales, Cardiff, Wales, United Kingdom

	NCT Number	Title	Other Names	Status	Conditions	Interventions	Characteristics	Population	Sponsor/ Collaborators	Funder Type	Dates	Locations
8	NCT02525159	Effectiveness of DIM Supplements to Increase 2-OHE1/16 Ratio<br>Study Documents:	Title Acronym: EDIMI216OHE1<br>Other Ids: SDEI.PTID.05.3(CM	Completed	•Breast Cancer	•Dietary Supplement: DIM pills<br>•Other: Placebo Pill	Study Type: Interventional<br>Phase: Phase 3<br>Study Design:<br>•Allocation: Randomized<br>•Intervention Model: Parallel Assignment<br>•Masking: Double (Participant, Investigator)<br>•Primary Purpose: Prevention<br>Outcome Measures:<br>•Change in ratio of estrogen metabolites 2OHE1:16#OHE1 in urine (REMU)<br>•Adherence<br>•Presence of Side Effects	Enrollment: 60<br>Age: 35 Years to 52 Years (Adult)<br>Sex: Female	•Instituto Nacional de Perinatologia Isidro Espinosa de los Reyes	•Other	Study Start: August 2006<br>Primary Completion: August 2009<br>Study Completion: February 2010<br>First Posted: August 17, 2015<br>Results First Posted: No Results Posted<br>Last Update Posted: August 17, 2015	
9	NCT00212381	Oral Diindolylmethane (DIM) for the Treatment of Cervical Dysplasia<br>Study Documents:	Title Acronym:<br>Other Ids: 9218	Completed	•Cervical Dysplasia	•Drug: di indolylmethane (DIM)<br>•Dietary Supplement: Red rice bran	Study Type: Interventional<br>Phase: Phase 3<br>Study Design:<br>•Allocation: Randomized<br>•Intervention Model: Parallel Assignment<br>•Masking: Quadruple (Participant, Care Provider, Investigator, Outcomes Assessor)<br>•Primary Purpose: Treatment<br>Outcome Measures:<br>•Measure the regression of CIN in women by cytology, colposcopy, and biopsy,<br>•HPV colonization by commercial ELIZA test<br>•Adverse events reported by subjects and lab abnormalities i.e. CBC and SMA20	Enrollment: 62<br>Age: 18 Years to 80 Years (Adult, Older Adult)<br>Sex: Female	•New York Presbyterian Hospital	•Other	Study Start: September 2000<br>Primary Completion: January 2006<br>Study Completion: July 2010<br>First Posted: September 21, 2005<br>Results First Posted: No Results Posted<br>Last Update Posted: March 29, 2017	•NYU School of Medicine, New York, New York, United States

	NCT Number	Title	Other Names	Status	Conditions	Interventions	Characteristics	Population	Sponsor/ Collaborators	Funder Type	Dates	Locations
10	NCT00784394	Diindolylmethane in Preventing Cancer in Healthy Volunteers<br><br>Study Documents:	Title Acronym:<br><br>Other Ids:<br>• NCI-2014-00523<br>• NO1-CN-35008-38<br>• CDR0000617334<br>• KUMC-HSC-9139<br>• N01-CN-35008-1<br>• N01CN35008	Completed	• Healthy, no Evidence of Disease	• Drug: diindolylmethane<br>• Other: placebo<br>• Other: pharmacological study<br>• Other: laboratory biomarker analysis<br>• Other: quality-of-life assessment	Study Type: Interventional<br><br>Phase: Phase 1<br><br>Study Design:<br>• Allocation: Randomized<br>• Intervention Model: Parallel Assignment<br>• Masking: Double (Participant, Investigator)<br>• Primary Purpose: Prevention<br><br>Outcome Measures:<br>• Maximum tolerated dose (MTD), defined as the highest dose at which no grade 2 or higher toxicities are seen<br>• Pharmacokinetics of diindolylmethane	Enrollment: 20<br><br>Age: 18 Years to 70 Years (Adult, Older Adult)<br><br>Sex: All	• National Cancer Institute (NCI)	• NIH	Study Start: April 2004<br><br>Primary Completion: October 2009<br><br>Study Completion: October 2009<br><br>First Posted: November 4, 2008<br><br>Results First Posted: No Results Posted<br><br>Last Update Posted: December 29, 2016	• University of Kansas Medical Center, Kansas City, Kansas, United States
11	NCT02197000	A Nutritional Intervention to Decrease Breast Density Among Female BRCA (BReast CAncer Susceptibility Gene) Carriers<br><br>Study Documents:	Title Acronym:<br><br>Other Ids:<br>0117-14-RMC	Completed	• BRCA1 Gene Mutation<br>• BRCA2 Gene Mutation	• Dietary Supplement: DIM-Avail 100mg	Study Type: Interventional<br><br>Phase: Not Applicable<br><br>Study Design:<br>• Allocation: N/A<br>• Intervention Model: Single Group Assignment<br>• Masking: None (Open Label)<br>• Primary Purpose: Prevention<br><br>Outcome Measures:<br>• Changes in breast density compared to baseline<br>• Estrogen profile<br>• The Estronex Profile<br>• change in Quality of life	Enrollment: 23<br><br>Age: 18 Years to 70 Years (Adult, Older Adult)<br><br>Sex: Female	• Rabin Medical Center	• Other	Study Start: November 11, 2014<br><br>Primary Completion: October 10, 2018<br><br>Study Completion: October 10, 2018<br><br>First Posted: July 22, 2014<br><br>Results First Posted: No Results Posted<br><br>Last Update Posted: November 12, 2020	• Rabin Medical Center, Beilinson Hospital, Petah-Tikva, Israel

	NCT Number	Title	Other Names	Status	Conditions	Interventions	Characteristics	Population	Sponsor/ Collaborators	Funder Type	Dates	Locations
12	NCT00591305	New Therapy of Laryngeal Papilloma In Children<br><br>Study Documents:	Title Acronym:<br><br>Other Ids:<br>• RDC-008287A<br>• 5R01DC008287	Terminated	• Papilloma	• Dietary Supplement: diindolylmethane (DIM)<br>• Device: 585 nm pulsed dye laser	Study Type:<br>Interventional<br><br>Phase:<br>Not Applicable<br><br>Study Design:<br>• Allocation: Randomized<br>• Intervention Model: Parallel Assignment<br>• Masking: Single (Participant)<br>• Primary Purpose: Prevention<br><br>Outcome Measures:<br>• Number of Cases With Recurrence of Laryngeal Papilloma in 5 Months<br>• Estradiol Level in Blood Pre Treatment<br>• Estradiol Level in Blood Post Treatment	Enrollment:<br>1<br><br>Age:<br>10 Years to 21 Years (Child, Adult)<br><br>Sex:<br>All	• Boston University<br>• National Institute on Deafness and Other Communication Disorders (NIDCD)	• Other<br>• NIH	Study Start:<br>September 2007<br><br>Primary Completion:<br>August 2011<br><br>Study Completion:<br>October 2011<br><br>First Posted:<br>January 11, 2008<br><br>Results First Posted:<br>May 18, 2017<br><br>Last Update Posted:<br>May 18, 2017	• Boston Medical Center, Boston, Massachusetts, United States
13	NCT02999399	Glucobrassicin-Brussel Sprout Effect on D10 Phe Metabolism<br><br>Study Documents:	Title Acronym:<br><br>Other Ids:<br>1610M96281	Completed	• Smoking	• Other: Brussel Sprouts<br>• Drug: Deuterated Phenanthrene	Study Type:<br>Interventional<br><br>Phase:<br>Phase 1<br><br>Study Design:<br>• Allocation: N/A<br>• Intervention Model: Single Group Assignment<br>• Masking: None (Open Label)<br>• Primary Purpose: Basic Science<br><br>Outcome Measures:<br>• Change in [D10]phenanthrene tetraol<br>• Change in [D10]phenanthrols<br>• Change in [D10]phenanthrene tetraol:[D10]phenanthrol ratio	Enrollment:<br>39<br><br>Age:<br>18 Years to 99 Years (Adult, Older Adult)<br><br>Sex:<br>All	• Masonic Cancer Center, University of Minnesota	• Other	Study Start:<br>December 1, 2016<br><br>Primary Completion:<br>March 22, 2021<br><br>Study Completion:<br>March 22, 2021<br><br>First Posted:<br>December 21, 2016<br><br>Results First Posted:<br>No Results Posted<br><br>Last Update Posted:<br>August 5, 2021	• University of Minnesota, Minneapolis, Minnesota, United States
