# Supplementary material for: Curcumin, thymoquinone, and 3, 3′-diindolylmethane combinations attenuate lung and liver cancers progression
Source: Front Pharmacol. 2022 Jun 29;13:936996. doi: 10.3389/fphar.2022.936996 (PMC9277483; doi:10.3389/fphar.2022.936996)
Supplement: Supplementary file 6 [file DataSheet1.PDF]

ClinicalTrials.gov Search Results 05/03/2022

	NCT Number	Title	Other Names	Status	Conditions	Interventions	Characteristics	Population	Sponsor/ Collaborators	Funder Type	Dates	Locations
1	NCT03769766	Trial of Curcumin to Prevent Progression of Low-risk Prostate Cancer Under Active Surveillance<br>Study Documents:	Title Acronym:<br>Other Ids: STU 012018-071	Recruiting	• Prostate Cancer	• Drug: Curcumin<br>• Drug: Placebo	Study Type: Interventional<br>Phase: Phase 3<br>Study Design:<br>• Allocation: Randomized<br>• Intervention Model: Parallel Assignment<br>• Masking: Double (Participant, Investigator)<br>• Primary Purpose: Treatment<br>Outcome Measures: The primary end point is rate of disease progression.	Enrollment: 291<br>Age: 40 Years to 89 Years (Adult, Older Adult)<br>Sex: Male	• University of Texas Southwestern Medical Center	• Other	Study Start: March 11, 2019<br>Primary Completion: November 2022<br>Study Completion: November 2026<br>First Posted: December 10, 2018<br>Results First Posted: No Results Posted<br>Last Update Posted: January 26, 2022	• UT Southwestern Medical Center, Dallas, Texas, United States
2	NCT00973869	Curcumin in Preventing Colorectal Cancer in Patients Undergoing Colorectal Endoscopy or Colorectal Surgery<br>Study Documents:	Title Acronym:<br>Other Ids:<br>• CDR0000648267<br>• LRI-UL-CURCUMIN<br>• EUDRACT-2007-0[illegible]<br>• EU-20961	Unknown status	• Colorectal Cancer	• Dietary Supplement: curcumin<br>• Other: high performance liquid chromatography<br>• Other: laboratory biomarker analysis<br>• Other: pharmacological study<br>• Procedure: diagnostic endoscopic procedure<br>• Procedure: therapeutic conventional surgery	Study Type: Interventional<br>Phase: Phase 1<br>Study Design: Primary Purpose: Prevention<br>Outcome Measures:<br>• Concentration of curcumin in colorectal tissue after treatment<br>• Tolerability and compliance<br>• Presence of curcumin and its metabolites in peripheral blood and urine	Enrollment: 30<br>Age: 18 Years and older (Adult, Older Adult)<br>Sex: All	• University of Leicester<br>• National Cancer Institute (NCI)	• Other	Study Start: July 2009<br>Primary Completion: January 2010<br>Study Completion:<br>First Posted: September 9, 2009<br>Results First Posted: No Results Posted<br>Last Update Posted: June 24, 2014	• St. Mark's Hospital, Harrow, England, United Kingdom<br>• Leicester Royal Infirmary, Leicester, England, United Kingdom

	NCT Number	Title	Other Names	Status	Conditions	Interventions	Characteristics	Population	Sponsor/ Collaborators	Funder Type	Dates	Locations
3	NCT03980509	A "Window Trial" on Curcumin for Invasive Breast Cancer Primary Tumors<br>Study Documents:	Title Acronym:<br>Other Ids: 103089	Recruiting	•Breast Cancer	•Drug: Curcumin	Study Type: Interventional<br>Phase: Phase 1<br>Study Design: •Allocation: N/A •Intervention Model: Single Group Assignment •Masking: None (Open Label) •Primary Purpose: Treatment<br>Outcome Measures: •Change in tumor proliferation rate •Number of adverse events reported	Enrollment: 20<br>Age: 18 Years and older (Adult, Older Adult)<br>Sex: All	•Medical University of South Carolina	•Other	Study Start: January 29, 2020<br>Primary Completion: June 30, 2022<br>Study Completion: December 30, 2022<br>First Posted: June 10, 2019<br>Results First Posted: No Results Posted<br>Last Update Posted: March 10, 2022	•Medical University of South Carolina, Charleston, South Carolina, United States
4	NCT01042938	Curcumin for the Prevention of Radiation-induced Dermatitis in Breast Cancer Patients<br>Study Documents:	Title Acronym:<br>Other Ids: •URCC1106 •5KL2RR024136-03 •05-238-80	Completed	•Breast Cancer	•Drug: Curcumin C3 Complex •Drug: Placebo	Study Type: Interventional<br>Phase: Phase 2<br>Study Design: •Allocation: Randomized •Intervention Model: Parallel Assignment •Masking: Quadruple (Participant, Care Provider, Investigator, Outcomes Assessor) •Primary Purpose: Treatment<br>Outcome Measures: •Severity of Dermatitis in Radiation Treatment Site in Breast Cancer Patients •Moist Desquamation at Radiation Treatment Site •Redness at Radiation Treatment Site •Pain at Radiation Treatment Site	Enrollment: 35<br>Age: 21 Years and older (Adult, Older Adult)<br>Sex: Female	•University of Rochester	•Other	Study Start: January 2008<br>Primary Completion: September 2010<br>Study Completion: April 2011<br>First Posted: January 6, 2010<br>Results First Posted: June 20, 2012<br>Last Update Posted: June 25, 2012	•University of Rochester Medical Center & Wilmot Cancer Center, Rochester, New York, United States

	NCT Number	Title	Other Names	Status	Conditions	Interventions	Characteristics	Population	Sponsor/ Collaborators	Funder Type	Dates	Locations
5	NCT02439385	Avastin/FOLFIRI in Combination With Curcumin in Colorectal Cancer Patients With Unresectable Metastasis<br><br>Study Documents:	Title Acronym:<br><br>Other Ids:<br>GAIRB2015-87	Completed	• Colorectal Cancer	• Drug: Avastin/ FOLFIRI<br>• Dietary Supplement: Curcumin	Study Type:<br>Interventional<br><br>Phase:<br>Phase 2<br><br>Study Design:<br>• Allocation: N/A<br>• Intervention Model: Single Group Assignment<br>• Masking: None (Open Label)<br>• Primary Purpose: Treatment<br><br>Outcome Measures:<br>• Progression-free survival<br>• Overall survival rate<br>• Overall response rate<br>• Safety (assessed by toxicity grades defined by NCI-CTCAE (version 4.0)<br>• Quality of life (QoL)<br>• Fatigue (FACIT-Fatigue scale (version 4, Korean version)	Enrollment:<br>44<br><br>Age:<br>20 Years and older (Adult, Older Adult)<br><br>Sex:<br>All	• Gachon University Gil Medical Center<br>• Aju Pharm	• Other	Study Start:<br>August 24, 2015<br><br>Primary Completion:<br>August 1, 2019<br><br>Study Completion:<br>August 1, 2019<br><br>First Posted:<br>May 8, 2015<br><br>Results First Posted:<br>No Results Posted<br><br>Last Update Posted:<br>March 31, 2022	

	NCT Number	Title	Other Names	Status	Conditions	Interventions	Characteristics	Population	Sponsor/ Collaborators	Funder Type	Dates	Locations
6	NCT03211104	Comparison of Duration of Treatment Interruption With or Without Curcumin During the Off Treatment Periods in Patients With Prostate Cancer Undergoing Intermittent Androgen Deprivation Therapy<br><br>Study Documents:	Title Acronym:<br><br>Other Ids: 2007-06-068	Completed	• Prostate Cancer	• Dietary Supplement: curcumin<br>• Dietary Supplement: Placebo	Study Type: Interventional<br><br>Phase: Not Applicable<br><br>Study Design:<br>• Allocation: Randomized<br>• Intervention Model: Parallel Assignment<br>• Masking: Double (Participant, Investigator)<br>• Primary Purpose: Treatment<br><br>Outcome Measures:<br>• Duration of treatment interruption with or without curcumin<br>• Mean change in PSA(ng/ml) from baseline between curcumin group versus placebo<br>• Mean change in testosterone(ng/ml) from baseline between curcumin group versus<br>• Adverse events	Enrollment: 107<br><br>Age: Child, Adult, Older Adult<br><br>Sex: Male	• Samsung Medical Center	• Other	Study Start: August 30, 2007<br><br>Primary Completion: August 5, 2015<br><br>Study Completion: August 5, 2015<br><br>First Posted: July 7, 2017<br><br>Results First Posted: No Results Posted<br><br>Last Update Posted: July 7, 2017	

	NCT Number	Title	Other Names	Status	Conditions	Interventions	Characteristics	Population	Sponsor/ Collaborators	Funder Type	Dates	Locations
7	NCT02724202	Curcumin in Combination With 5FU for Colon Cancer<br><br>Study Documents:	Title Acronym:<br><br>Other Ids:<br>014-143	Unknown status	• Metastatic Colon Cancer	• Drug: Curcumin<br>• Drug: 5-flurorouracil	Study Type:<br>Interventional<br><br>Phase:<br>Early Phase 1<br><br>Study Design:<br>• Allocation: N/A<br>• Intervention Model: Single Group Assignment<br>• Masking: None (Open Label)<br>• Primary Purpose: Treatment<br><br>Outcome Measures:<br>• Determine the safety using curcumin in patients with metastatic colon cancer; where toxicities will be graded according to the NCI Common Terminology Criteria for Adverse Events (CTCAE) Version 4.0.<br>• Overall Response<br>• Evidence of altered biomarker status (circulating DNA methylation status, miRNA profile) at 8 weeks post-treatment according to RECIST version 1.1 and survival criteria.<br>• Duration of response<br>• Duration of progression free survival<br>• Duration of overall survival<br>• Duration of Quality of Life	Enrollment:<br>13<br><br>Age:<br>18 Years and older (Adult, Older Adult)<br><br>Sex:<br>All	• Baylor Research Institute	• Other	Study Start:<br>March 2016<br><br>Primary Completion:<br>March 13, 2020<br><br>Study Completion:<br>June 13, 2020<br><br>First Posted:<br>March 31, 2016<br><br>Results First Posted:<br>No Results Posted<br><br>Last Update Posted:<br>January 18, 2020	• Baylor Charles A. Sammons Cancer Center, Dallas, Texas, United States

	NCT Number	Title	Other Names	Status	Conditions	Interventions	Characteristics	Population	Sponsor/ Collaborators	Funder Type	Dates	Locations
8	NCT01160302	Curcumin Biomarker Trial in Head and Neck Cancer<br><br>Study Documents:	Title Acronym:<br><br>Other Ids:<br>•H08-081<br>•1R21CA137545-01<br>•FWCC	Completed	•Head and Neck Cancer	•Drug: Microgranular Curcumin C3 Complex®	Study Type:<br>Interventional<br><br>Phase:<br>Early Phase 1<br><br>Study Design:<br>•Allocation: N/A<br>•Intervention Model: Single Group Assignment<br>•Masking: None (Open Label)<br>•Primary Purpose: Basic Science<br><br>Outcome Measures:<br>•Change in tissue biomarkers<br>•Pharmacokinetics of microgranular curcumin<br>•Ease of ingestion	Enrollment:<br>33<br><br>Age:<br>18 Years to 90 Years (Adult, Older Adult)<br><br>Sex:<br>All	•Louisiana State University Health Sciences Center Shreveport<br>•Feist-Weiller Cancer Center at Louisiana State University Health Sciences<br>•National Cancer Institute (NCI)	•Other<br>•NIH	Study Start:<br>June 2010<br><br>Primary Completion:<br>January 2016<br><br>Study Completion:<br>January 2016<br><br>First Posted:<br>July 12, 2010<br><br>Results First Posted:<br>No Results Posted<br><br>Last Update Posted:<br>March 2, 2016	•LSUHSC-Shreveport and Feist-Weiller Cancer Center, Shreveport, Louisiana, United States
9	NCT03847623	Effect of Preoperative Curcumin in Breast Cancer Patients<br><br>Study Documents:	Title Acronym:<br>EPC<br><br>Other Ids:<br>20149-582	Unknown status	•Breast Cancer	•Dietary Supplement: Curcumin<br>•Other: Placebo	Study Type:<br>Interventional<br><br>Phase:<br>Not Applicable<br><br>Study Design:<br>•Allocation: Randomized<br>•Intervention Model: Parallel Assignment<br>•Masking: Quadruple (Participant, Care Provider, Investigator, Outcomes Assessor)<br>•Primary Purpose: Other<br><br>Outcome Measures:<br>•Tumour infiltrating lymphocytes (TILs)<br>•FOXP3<br>•CD68	Enrollment:<br>30<br><br>Age:<br>Child, Adult, Older Adult<br><br>Sex:<br>Female	•University of Malaya	•Other	Study Start:<br>June 18, 2017<br><br>Primary Completion:<br>December 31, 2020<br><br>Study Completion:<br>December 31, 2020<br><br>First Posted:<br>February 20, 2019<br><br>Results First Posted:<br>No Results Posted<br><br>Last Update Posted:<br>March 25, 2020	•University of Malaya Medical Center, Kuala Lumpur, Malaysia

	NCT Number	Title	Other Names	Status	Conditions	Interventions	Characteristics	Population	Sponsor/ Collaborators	Funder Type	Dates	Locations
10	NCT03072992	"Curcumin" in Combination With Chemotherapy in Advanced Breast Cancer<br><br>Study Documents:	Title Acronym:<br><br>Other Ids:<br>5592-17-02-23	Completed	• Advanced Breast Cancer<br>• Metastatic Breast Cancer	• Drug: Curcumin<br>• Drug: Paclitaxel<br>• Drug: Placebo	Study Type:<br>Interventional<br><br>Phase:<br>Phase 2<br><br>Study Design:<br>• Allocation: Randomized<br>• Intervention Model: Parallel Assignment<br>• Masking: Triple (Participant, Care Provider, Investigator)<br>• Primary Purpose: Treatment<br><br>Outcome Measures:<br>• Objective response rate<br>• Adverse events<br>• Global Health Status/QoL scale<br>• Progression free survival<br>• Time to Treatment Failure<br>• Time to Tumour Progression	Enrollment:<br>150<br><br>Age:<br>18 Years to 75 Years (Adult, Older Adult)<br><br>Sex:<br>Female	• National Center of Oncology, Armenia<br>• BRIU GmbH	• Other	Study Start:<br>March 20, 2017<br><br>Primary Completion:<br>November 20, 2018<br><br>Study Completion:<br>June 30, 2019<br><br>First Posted:<br>March 8, 2017<br><br>Results First Posted:<br>No Results Posted<br><br>Last Update Posted:<br>November 27, 2019	• National Center of Oncology, Yerevan, Armenia
11	NCT01859858	Effect of Curcumin on Dose Limiting Toxicity and Pharmacokinetics of Irinotecan in Patients With Solid Tumors<br><br>Study Documents:	Title Acronym:<br><br>Other Ids:<br>LCCC 1227	Completed	• Advanced Colorectal Cancer	• Dietary Supplement: curcumin<br>• Drug: Irinotecan	Study Type:<br>Interventional<br><br>Phase:<br>Phase 1<br><br>Study Design:<br>• Allocation: Non-Randomized<br>• Intervention Model: Single Group Assignment<br>• Masking: None (Open Label)<br>• Primary Purpose: Basic Science<br><br>Outcome Measures:<br>• Maximum tolerated dose (MTD)<br>• Pharmacokinetics of irinotecan<br>• Pharmacokinetics of SN-38	Enrollment:<br>23<br><br>Age:<br>21 Years to 99 Years (Adult, Older Adult)<br><br>Sex:<br>All	• UNC Lineberger Comprehensive Cancer Center	• Other	Study Start:<br>June 2013<br><br>Primary Completion:<br>October 5, 2016<br><br>Study Completion:<br>October 5, 2016<br><br>First Posted:<br>May 22, 2013<br><br>Results First Posted:<br>No Results Posted<br><br>Last Update Posted:<br>June 22, 2020	• IU Simon Cancer Center, Indianapolis, Indiana, United States<br>• University of North Carolina at Chapel Hill Lineberger Comprehensive cancer Center, Chapel Hill, North Carolina, United States

	NCT Number	Title	Other Names	Status	Conditions	Interventions	Characteristics	Population	Sponsor/ Collaborators	Funder Type	Dates	Locations
12	NCT01490996	Combining Curcumin With FOLFOX Chemotherapy in Patients With Inoperable Colorectal Cancer<br><br>Study Documents:	Title Acronym: CUFOX<br><br>Other Ids: 0225	Completed	•Colonic Cancer<br>•Metastasis	•Drug: Oral complex C3 curcumin + chemotherapy<br>•Drug: Chemotherapy only	Study Type: Interventional<br><br>Phase:<br>•Phase 1<br>•Phase 2<br><br>Study Design:<br>•Allocation: Randomized<br>•Intervention Model: Parallel Assignment<br>•Masking: None (Open Label)<br>•Primary Purpose: Treatment<br><br>Outcome Measures:<br>•Completion of dose escalation over 2 cycles of therapy<br>•Completion of (or withdrawal from) chemotherapy<br>•Efficacy in terms of disease response and survival	Enrollment: 41<br><br>Age: 18 Years and older (Adult, Older Adult)<br><br>Sex: All	•University of Leicester	•Other	Study Start: February 2012<br><br>Primary Completion: May 31, 2017<br><br>Study Completion: May 31, 2017<br><br>First Posted: December 13, 2011<br><br>Results First Posted: No Results Posted<br><br>Last Update Posted: January 31, 2020	•Dept Oncology, Leicester Royal Infirmary, Leicester, United Kingdom

	NCT Number	Title	Other Names	Status	Conditions	Interventions	Characteristics	Population	Sponsor/ Collaborators	Funder Type	Dates	Locations
13	NCT01917890	Radiosensitizing and Radioprotectve Effects of Curcumin in Prostate Cancer<br><br>Study Documents:	Title Acronym:<br><br>Other Ids:<br>Radiation Therapy And Curcumin	Completed	• Prostate Cancer<br>• Radiation Therapy	• Dietary Supplement: Curcumin<br>• Dietary Supplement: Placebo	Study Type:<br>Interventional<br><br>Phase:<br>Not Applicable<br><br>Study Design:<br>• Allocation: Randomized<br>• Intervention Model: Parallel Assignment<br>• Masking: Double (Participant, Investigator)<br>• Primary Purpose: Supportive Care<br><br>Outcome Measures:<br>• Biochemical or clinical progression-free survival<br>• Quality of life<br>• Sexual dysfunction score<br>• C-reactive protein (hs-CRP)<br>• Inflammatory factors (tumor necrosis factor alpha (TNF-alpha), Interleukin 1 beta (IL1-beta)and Interleukin 6 (ILI-6))<br>• Antioxidant enzymes (Catalase, super oxide dismutase (SOD), glutathione-S- transferase (GST), glutathione peroxidase (GPX))<br>• cycloxygenase 2 (COX2)<br>• Nuclear factor KB (NF-#B)	Enrollment:<br>40<br><br>Age:<br>50 Years to 80 Years (Adult, Older Adult)<br><br>Sex:<br>Male	• Shahid Beheshti University of Medical Sciences	• Other	Study Start:<br>March 2011<br><br>Primary Completion:<br>October 2013<br><br>Study Completion:<br>October 2013<br><br>First Posted:<br>August 7, 2013<br><br>Results First Posted:<br>No Results Posted<br><br>Last Update Posted:<br>November 18, 2015	• Oncology and radiotherapy department, Besat Hospital, Tehran, Iran, Islamic Republic of

	NCT Number	Title	Other Names	Status	Conditions	Interventions	Characteristics	Population	Sponsor/ Collaborators	Funder Type	Dates	Locations
14	NCT00192842	Gemcitabine With Curcumin for Pancreatic Cancer<br><br>Study Documents:	Title Acronym:<br><br>Other Ids:<br>RonCurcuminPancre	Completed	• Pancreatic Cancer	• Drug: curcumin (+ gemcitabine)	Study Type:<br>Interventional<br><br>Phase:<br>Phase 2<br><br>Study Design:<br>• Allocation: Non-Randomized<br>• Intervention Model: Single Group Assignment<br>• Masking: None (Open Label)<br>• Primary Purpose: Treatment<br><br>Outcome Measures:<br>• time to tumor progression<br>• response rate<br>• survival<br>• clinical benefit<br>• toxicity	Enrollment:<br>17<br><br>Age:<br>18 Years and older (Adult, Older Adult)<br><br>Sex:<br>All	• Rambam Health Care Campus	• Other	Study Start:<br>July 2004<br><br>Primary Completion:<br>November 2007<br><br>Study Completion:<br>September 2010<br><br>First Posted:<br>September 19, 2005<br><br>Results First Posted:<br>No Results Posted<br><br>Last Update Posted:<br>September 22, 2010	
15	NCT03865992	Curcumin in Reducing Joint Pain in Breast Cancer Survivors With Aromatase Inhibitor-Induced Joint Disease<br><br>Study Documents:	Title Acronym:<br><br>Other Ids:<br>• 18432<br>• NCI-2018-03787<br>• UG1CA189823	Active, not recruiting	• Breast Cancer<br>• Joint Pain	• Dietary Supplement: Curcumin<br>• Other: Placebo<br>• Other: Nanoemulsion<br>• Other: Quality-of-Life Assessment<br>• Behavioral: Questionnaire	Study Type:<br>Interventional<br><br>Phase:<br>Not Applicable<br><br>Study Design:<br>• Allocation: Randomized<br>• Intervention Model: Parallel Assignment<br>• Masking: Double (Participant, Investigator)<br>• Primary Purpose: Supportive Care<br><br>Outcome Measures:<br>• Changes in aromatase inhibitor-induced symptoms and overall wellbeing in postmenopausal women on aromatase inhibitor therapy<br>• Change in Brief Pain Inventory (BPI) pain score<br>• Incidence of adverse events<br>• Change in FACT-ES score<br>• Change in DASH score	Enrollment:<br>42<br><br>Age:<br>Child, Adult, Older Adult<br><br>Sex:<br>Female	• City of Hope Medical Center<br>• National Cancer Institute (NCI)	• Other<br>• NIH	Study Start:<br>March 4, 2019<br><br>Primary Completion:<br>July 31, 2022<br><br>Study Completion:<br>July 31, 2022<br><br>First Posted:<br>March 7, 2019<br><br>Results First Posted:<br>No Results Posted<br><br>Last Update Posted:<br>September 16, 2021	• City of Hope Medical Center, Duarte, California, United States<br>• City of Hope Rancho Cucamonga, Rancho Cucamonga, California, United States<br>• City of Hope South Pasadena, South Pasadena, California, United States<br>• Ohio State University Comprehensive Cancer Center, Columbus, Ohio, United States

	NCT Number	Title	Other Names	Status	Conditions	Interventions	Characteristics	Population	Sponsor/ Collaborators	Funder Type	Dates	Locations
16	NCT01740323	Phase II Study of Curcumin vs Placebo for Chemotherapy-Treated Breast Cancer Patients Undergoing Radiotherapy<br><br>Study Documents:<br>• Study Protocol and Statistical Analysis Plan	Title Acronym:<br><br>Other Ids:<br>• IRB00055328<br>• Winship2139-11<br>• R21CA178603	Completed	• Breast Cancer	• Drug: Placebo<br>• Drug: Curcumin	Study Type:<br>Interventional<br><br>Phase:<br>Phase 2<br><br>Study Design:<br>• Allocation: Randomized<br>• Intervention Model: Parallel Assignment<br>• Masking: Double (Participant, Investigator)<br>• Primary Purpose: Treatment<br><br>Outcome Measures:<br>• PBMC NF-kB DNA Binding Measured in ng/Well<br>• Plasma TNF-alpha<br>• Plasma sTNFR2 Measured in pg/ml<br>• Plasma IL-1ra Measured in pg/ml<br>• Plasma IL-6 Measured in pg/ml<br>• Plasma C-reactive Protein (CRP) Measured in mg/L<br>• Fatigue	Enrollment:<br>30<br><br>Age:<br>18 Years and older (Adult, Older Adult)<br><br>Sex:<br>Female	• Andrew H Miller<br>• National Cancer Institute (NCI)<br>• Emory University	• Other<br>• NIH	Study Start:<br>May 2015<br><br>Primary Completion:<br>July 27, 2018<br><br>Study Completion:<br>July 27, 2018<br><br>First Posted:<br>December 4, 2012<br><br>Results First Posted:<br>September 18, 2019<br><br>Last Update Posted:<br>September 18, 2019	• Emory University, Atlanta, Georgia, United States

	NCT Number	Title	Other Names	Status	Conditions	Interventions	Characteristics	Population	Sponsor/ Collaborators	Funder Type	Dates	Locations
17	NCT02321293	A Open-label Prospective Cohort Trial of Curcumin Plus Tyrosine Kinase Inhibitors (TKI) for EGFR -Mutant Advanced NSCLC<br><br>Study Documents:	Title Acronym: CURCUMIN<br><br>Other Ids: JGH-14-149	Unknown status	• Lung Cancer	• Dietary Supplement: CurcuVIVA™<br>• Drug: Tyrosine Kinase Inhibitor gefitinib (Iressa)<br>• Drug: Tyrosine Kinase Inhibitor erlotinib (Tarceva)	Study Type: Interventional<br><br>Phase: Phase 1<br><br>Study Design:<br>• Allocation: N/A<br>• Intervention Model: Single Group Assignment<br>• Masking: None (Open Label)<br><br>Outcome Measures:<br>• feasibility assessed by: Willingness of patients to participate= Number of enrolled/Number of approached patients,<br>• feasibility assessed by follow-up rate= number of actual study visits/ total number of study visits<br>• feasibility Adherence/ Compliance rate= Number of taken capsules/Total number of capsules<br>• feasibility Questionnaires completion rate= Number of completed questionnaires/Total number of questionnaires<br>• safety number of side effects<br>• To evaluate and compare the changes in health-related quality of life before and after<br>• To evaluate anti-inflammatory properties of Curcumin (assessed by measuring C-reactive protein)	Enrollment: 20<br><br>Age: 18 Years and older (Adult, Older Adult)<br><br>Sex: All	• Lady Davis Institute<br>• Jewish General Hospital	• Other	Study Start: August 2015<br><br>Primary Completion: August 2016<br><br>Study Completion: December 2016<br><br>First Posted: December 22, 2014<br><br>Results First Posted: No Results Posted<br><br>Last Update Posted: September 2, 2015	• Peter Brojge Lung Cancer Center, Jewish General Hospital, Montreal, Quebec, Canada

	NCT Number	Title	Other Names	Status	Conditions	Interventions	Characteristics	Population	Sponsor/ Collaborators	Funder Type	Dates	Locations
18	NCT04208334	The Effect of Curcumin for Treatment of Cancer Anorexia-Cachexia Syndrome in Patients With Stage III-IV of Head and Neck Cancer<br><br>Study Documents:	Title Acronym: CurChexia<br><br>Other Ids: R119h/61	Completed	•Cancer Cachexia<br>•Head and Neck Cancer<br>•Head and Neck Neoplasms	•Dietary Supplement: Curcumin<br>•Other: Placebo	Study Type: Interventional<br><br>Phase: Phase 2<br><br>Study Design:<br>•Allocation: Randomized<br>•Intervention Model: Parallel Assignment<br>•Masking: Quadruple (Participant, Care Provider, Investigator, Outcomes Assessor)<br>•Primary Purpose: Prevention<br><br>Outcome Measures:<br>•Muscle mass<br>•BMI<br>•Hand grips muscle strength<br>•serum NF-kB level	Enrollment: 20<br><br>Age: 18 Years to 75 Years (Adult, Older Adult)<br><br>Sex: All	•Phramongkutklao College of Medicine and Hospital	•Other	Study Start: February 13, 2020<br><br>Primary Completion: February 24, 2021<br><br>Study Completion: March 31, 2021<br><br>First Posted: December 23, 2019<br><br>Results First Posted: No Results Posted<br><br>Last Update Posted: May 28, 2021	•Phramongkutklao Hospital, Ratchathewi, Bangkok, Thailand
19	NCT04294836	Curcumin in Advanced Cervical Cancer<br><br>Study Documents:	Title Acronym:<br><br>Other Ids: IX-023435	Not yet recruiting	•Cervical Cancer, Stage IIB	•Drug: Curcumin<br>•Drug: Placebo oral tablet	Study Type: Interventional<br><br>Phase: Phase 2<br><br>Study Design:<br>•Allocation: Randomized<br>•Intervention Model: Parallel Assignment<br>•Masking: Double (Participant, Investigator)<br>•Primary Purpose: Treatment<br><br>Outcome Measures:<br>•Compare overall survival and progression free survival<br>•Compare the objective response rate using RECIST radiological criteria<br>•Compare the disease control rate<br>•Describe the safety of research therapy by classifying Common Terminology Criteria for Adverse Events (CTCAE)	Enrollment: 240<br><br>Age: 18 Years to 70 Years (Adult, Older Adult)<br><br>Sex: Female	•Instituto Nacional de Cancerologia, Columbia	•Other	Study Start: December 1, 2021<br><br>Primary Completion: January 31, 2023<br><br>Study Completion: December 31, 2023<br><br>First Posted: March 4, 2020<br><br>Results First Posted: No Results Posted<br><br>Last Update Posted: June 25, 2021	

	NCT Number	Title	Other Names	Status	Conditions	Interventions	Characteristics	Population	Sponsor/ Collaborators	Funder Type	Dates	Locations
20	NCT00094445	Trial of Curcumin in Advanced Pancreatic Cancer<br><br>Study Documents:	Title Acronym:<br><br>Other Ids:<br>• ID03-0009<br>• 1R21CA104337<br>• NCI-2012-01309	Completed	• Pancreatic Neoplasms<br>• Adenocarcinoma	• Drug: Curcumin	Study Type: Interventional<br><br>Phase: Phase 2<br><br>Study Design:<br>• Allocation: N/A<br>• Intervention Model: Single Group Assignment<br>• Masking: None (Open Label)<br>• Primary Purpose: Treatment<br><br>Outcome Measures: Six-Month Participant Survival	Enrollment: 50<br><br>Age: 18 Years and older (Adult, Older Adult)<br><br>Sex: All	• M.D. Anderson Cancer Center<br>• Sabinsa Corporation	• Other<br>• Industry	Study Start: November 2004<br><br>Primary Completion: April 2014<br><br>Study Completion: April 2014<br><br>First Posted: October 19, 2004<br><br>Results First Posted: August 28, 2020<br><br>Last Update Posted: August 28, 2020	• UT MD Anderson Cancer Center, Houston, Texas, United States
21	NCT01333917	Curcumin Biomarkers<br><br>Study Documents:	Title Acronym:<br><br>Other Ids: 10-1524	Completed	• Colorectal Cancer	• Drug: Curcumin C3 tablet	Study Type: Interventional<br><br>Phase: Phase 1<br><br>Study Design:<br>• Allocation: N/A<br>• Intervention Model: Single Group Assignment<br>• Masking: None (Open Label)<br><br>Outcome Measures:<br>• Gene expression<br>• Ribonucleic acid (RNA) level<br>• Apoptosis<br>• Number of Participants with Adverse Events	Enrollment: 40<br><br>Age: 40 Years to 80 Years (Adult, Older Adult)<br><br>Sex: All	• University of North Carolina, Chapel Hill	• Other	Study Start: November 2010<br><br>Primary Completion: October 2011<br><br>Study Completion: January 2013<br><br>First Posted: April 12, 2011<br><br>Results First Posted: No Results Posted<br><br>Last Update Posted: February 7, 2013	• UNC Department of Family Medicine, Chapel Hill, North Carolina, United States

	NCT Number	Title	Other Names	Status	Conditions	Interventions	Characteristics	Population	Sponsor/ Collaborators	Funder Type	Dates	Locations
22	NCT01294072	Study Investigating the Ability of Plant Exosomes to Deliver Curcumin to Normal and Colon Cancer Tissue<br><br>Study Documents:	Title Acronym:<br><br>Other Ids:<br>BCC-GI-10<br>Curcumin	Recruiting	• Colon Cancer	• Dietary Supplement: curcumin<br>• Dietary Supplement: Curcumin conjugated with plant exosomes<br>• Other: No intervention	Study Type:<br>Interventional<br><br>Phase:<br>Phase 1<br><br>Study Design:<br>• Allocation: Randomized<br>• Intervention Model: Parallel Assignment<br>• Masking: None (Open Label)<br>• Primary Purpose: Basic Science<br><br>Outcome Measures:<br>• Concentration of curcumin in normal and cancerous tissue<br>• safety and tolerability of curcumin alone as determined by adverse events<br>• effects of curcumin on normal and cancerous colon cells by measuring the biomarkers using histochemical staining<br>• the immune system response to curcumin, measured by serum cytokine levels<br>• immune response in ex vivo cell cultures of colon cancer cells treated with curcumin and Exo-cur, to be evaluated by using histochemical staining<br>• measurement of curcumin alone on metabolic characteristics of normal colon mucosa and colon tumors<br>• safety and tolerability of curcumin with plant exosomes as determined by adverse events<br>• measurement of curcumin mixed with plant exosomes on metabolic characteristics of normal colon mucosa and colon tumors	Enrollment:<br>35<br><br>Age:<br>20 Years and older (Adult, Older Adult)<br><br>Sex:<br>All	• University of Louisville	• Other	Study Start:<br>January 2011<br><br>Primary Completion:<br>January 2022<br><br>Study Completion:<br>December 2022<br><br>First Posted:<br>February 11, 2011<br><br>Results First Posted:<br>No Results Posted<br><br>Last Update Posted:<br>June 18, 2021	• University of Louisville Hospital, Louisville, Kentucky, United States

	NCT Number	Title	Other Names	Status	Conditions	Interventions	Characteristics	Population	Sponsor/ Collaborators	Funder Type	Dates	Locations
23	NCT00027495	Curcumin for the Prevention of Colon Cancer<br>Study Documents:	Title Acronym:<br>Other Ids:<br>• CDR0000067916<br>• P30CA046592<br>• CCUM-9941<br>• NCI-P00-0144	Completed	• Colorectal Cancer	• Dietary Supplement: curcumin	Study Type: Interventional<br>Phase: Phase 1<br>Study Design: Primary Purpose: Prevention<br>Outcome Measures:	Enrollment:<br>Age: 18 Years and older (Adult, Older Adult)<br>Sex: All	• University of Michigan Rogel Cancer Center<br>• National Cancer Institute (NCI)	• Other<br>• NIH	Study Start: December 2001<br>Primary Completion: February 2004<br>Study Completion: September 2007<br>First Posted: January 27, 2003<br>Results First Posted: No Results Posted<br>Last Update Posted: December 21, 2012	• University of Michigan Comprehensive Cancer Center, Ann Arbor, Michigan, United States
24	NCT02554344	Effect of Curcumin in Treatment of Squamous Cervical Intraepithelial Neoplasias (CINs)<br>Study Documents:	Title Acronym:<br>Other Ids: 015-074	Unknown status	• Cervical Intraepithelial Neoplasia	• Drug: Curcumin	Study Type: Interventional<br>Phase: Early Phase 1<br>Study Design:<br>• Allocation: N/A<br>• Intervention Model: Single Group Assignment<br>• Masking: None (Open Label)<br>• Primary Purpose: Treatment<br>Outcome Measures:<br>• Determine the safety and feasibility using curcumin in patients with CIN3 where toxicities will be graded according to the NCI Common Terminology Criteria for Adverse Events (CTCAE) Version 4.0.<br>• Regression Rate<br>• Overall Response<br>• Pathologic Response<br>• Rate of patients in which p65, phosphorylated p65, and acetylated p65 play a role in the persistence of CIN.<br>• Evaluation of patients with CIN3 for the presence of high-risk HPV.	Enrollment: 14<br>Age: 21 Years and older (Adult, Older Adult)<br>Sex: Female	• Baylor Research Institute	• Other	Study Start: March 2016<br>Primary Completion: January 2017<br>Study Completion: January 2017<br>First Posted: September 18, 2015<br>Results First Posted: No Results Posted<br>Last Update Posted: March 17, 2016	• Baylor Charles A. Sammons Cancer Center, Dallas, Texas, United States

	NCT Number	Title	Other Names	Status	Conditions	Interventions	Characteristics	Population	Sponsor/ Collaborators	Funder Type	Dates	Locations
25	NCT00295035	Phase III Trial of Gemcitabine, Curcumin and Celebrex in Patients With Metastatic Colon Cancer<br><br>Study Documents:	Title Acronym:<br><br>Other Ids:<br>tasmc-05-na-05160-ctil	Unknown status	•Colon Neoplasm	•Drug: CELECOXIB<br>•Drug: CURCUMIN	Study Type:<br>Interventional<br><br>Phase:<br>Phase 3<br><br>Study Design:<br>•Allocation: Randomized<br>•Intervention Model: Single Group Assignment<br>•Masking: Double<br>•Primary Purpose: Treatment<br><br>Outcome Measures:	Enrollment:<br>100<br><br>Age:<br>18 Years and older (Adult, Older Adult)<br><br>Sex:<br>All	•Tel-Aviv Sourasky Medical Center	•Other	Study Start:<br>March 2006<br><br>Primary Completion:<br><br>Study Completion:<br>March 2007<br><br>First Posted:<br>February 22, 2006<br><br>Results First Posted:<br>No Results Posted<br><br>Last Update Posted:<br>February 22, 2006	
26	NCT02724618	Nanocurcumin for Prostate Cancer Patients Undergoing Radiotherapy (RT)<br><br>Study Documents:	Title Acronym:<br><br>Other Ids:<br>406	Unknown status	•Prostate Cancer	•Drug: Curcumin<br>•Radiation: RT<br>•Drug: Placebo	Study Type:<br>Interventional<br><br>Phase:<br>Phase 2<br><br>Study Design:<br>•Allocation: Randomized<br>•Intervention Model: Parallel Assignment<br>•Masking: Quadruple (Participant, Care Provider, Investigator, Outcomes Assessor)<br>•Primary Purpose: Supportive Care<br><br>Outcome Measures:<br>•Proctitis [assessed using Common terminology criteria for adverse events (CTCAE)]<br>•Cystitis [assessed using CTCAE Grading Criteria]<br>•Hematologic Toxicity<br>•Biochemical progression-free survival (b-PFS)<br>•Treatment Response	Enrollment:<br>64<br><br>Age:<br>Child, Adult, Older Adult<br><br>Sex:<br>Male	•Shahid Beheshti University of Medical Sciences<br>•Behnam Daheshpour Charity Organization, Tehran, Iran	•Other	Study Start:<br>March 2016<br><br>Primary Completion:<br>April 15, 2017<br><br>Study Completion:<br>April 2022<br><br>First Posted:<br>March 31, 2016<br><br>Results First Posted:<br>No Results Posted<br><br>Last Update Posted:<br>October 12, 2017	•Shohada-e-Tajrish Medical Center, Tehran, Iran, Islamic Republic of

	NCT Number	Title	Other Names	Status	Conditions	Interventions	Characteristics	Population	Sponsor/ Collaborators	Funder Type	Dates	Locations
27	NCT02064673	Adjuvant Curcumin to Assess Recurrence Free Survival in Patients Who Have Had a Radical Prostatectomy<br>Study Documents:	Title Acronym:<br>Other Ids: STU 042013-080	Recruiting	• Prostate Cancer	• Drug: Curcumin<br>• Drug: placebo	Study Type: Interventional<br>Phase: Phase 3<br>Study Design:<br>• Allocation: Randomized<br>• Intervention Model: Single Group Assignment<br>• Masking: Double (Participant, Investigator)<br>• Primary Purpose: Treatment<br>Outcome Measures: Serum prostate specific antigen	Enrollment: 620<br>Age: 30 Years to 80 Years (Adult, Older Adult)<br>Sex: Male	• Yair Lotan<br>• University of Texas Southwestern Medical Center	• Other	Study Start: May 2014<br>Primary Completion: June 2022<br>Study Completion: June 2023<br>First Posted: February 17, 2014<br>Results First Posted: No Results Posted<br>Last Update Posted: February 28, 2022	• UT Southwestern Medical Center, Dallas, Texas, United States
28	NCT03290417	Correlative Analysis of the Genomics of Vitamin D and Omega-3 Fatty Acid Intake in Prostate Cancer<br>Study Documents:	Title Acronym:<br>Other Ids: CASE3816	Completed	• Prostate Cancer	• Dietary Supplement: Vitamin D<br>• Dietary Supplement: Omega-3<br>• Dietary Supplement: Turmeric	Study Type: Interventional<br>Phase: Not Applicable<br>Study Design:<br>• Allocation: Randomized<br>• Intervention Model: Parallel Assignment<br>• Masking: None (Open Label)<br>• Primary Purpose: Treatment<br>Outcome Measures:<br>• gene expression of very low and low risk prostate cancer patients on Active Surveillance<br>• Active Surveillance Failure<br>• Time to Active Surveillance Failure	Enrollment: 37<br>Age: Child, Adult, Older Adult<br>Sex: Male	• Case Comprehensive Cancer Center	• Other	Study Start: September 7, 2017<br>Primary Completion: June 20, 2019<br>Study Completion: December 20, 2019<br>First Posted: September 21, 2017<br>Results First Posted: No Results Posted<br>Last Update Posted: February 18, 2020	• Cleveland Clinic, Case Comprehensive Cancer Center, Cleveland, Ohio, United States

	NCT Number	Title	Other Names	Status	Conditions	Interventions	Characteristics	Population	Sponsor/ Collaborators	Funder Type	Dates	Locations
29	NCT04731844	Curcumin and Piperine in Patients on Surveillance for Monoclonal Gammopathy, Smoldering Myeloma or Prostate Cancer<br><br>Study Documents:	Title Acronym:<br><br>Other Ids:<br>UMLT20123	Recruiting	• Prostate Cancer<br>• Multiple Myeloma<br>• Smoldering Multiple Myeloma (SMM)<br>• Monoclonal Gammopathy of Undetermined Significance	• Drug: Curcumin plus Piperine	Study Type:<br>Interventional<br><br>Phase:<br>Phase 2<br><br>Study Design:<br>• Allocation: Non-Randomized<br>• Intervention Model: Parallel Assignment<br>• Masking: None (Open Label)<br>• Primary Purpose: Treatment<br><br>Outcome Measures:<br>• Response rate of Curcumin & Piperine supplementation in patients on AS for either early stage prostate cancer or MGUS.<br>• Progression Free Survival	Enrollment:<br>40<br><br>Age:<br>18 Years and older (Adult, Older Adult)<br><br>Sex:<br>All	• University of Rochester	• Other	Study Start:<br>December 14, 2021<br><br>Primary Completion:<br>May 31, 2023<br><br>Study Completion:<br>May 31, 2023<br><br>First Posted:<br>February 1, 2021<br><br>Results First Posted:<br>No Results Posted<br><br>Last Update Posted:<br>February 23, 2022	• University of Rochester, Rochester, New York, United States

	NCT Number	Title	Other Names	Status	Conditions	Interventions	Characteristics	Population	Sponsor/ Collaborators	Funder Type	Dates	Locations
30	NCT02138955	A Phase IB Dose Escalation Study of Lipocurc in Patients With Cancer<br><br>Study Documents:	Title Acronym:<br><br>Other Ids:<br>• Lipocurc1002/ P-1-010<br>• 0011594-24<br>• SPP1002<br>• 2013-001594-24	Unknown status	• Patients With Advanced Cancer Who Have Failed Standard of Care Therapy	• Drug: Liposomeal curcumin	Study Type:<br>Interventional<br><br>Phase:<br>• Phase 1<br>• Phase 2<br><br>Study Design:<br>• Allocation: N/A<br>• Intervention Model: Single Group Assignment<br>• Masking: None (Open Label)<br>• Primary Purpose: Treatment<br><br>Outcome Measures:<br>• Safety<br>• Determine the maximum tolerated dose of lipocurc<br>• Maximum tolerated dose<br>• Maximum tolerated dose defined by < Grade 1 hematologic toxicity,<br>• Response by Recist criteria<br>• Tumor response by resist criteria<br>• Tumor response<br>• Benefit<br>• Objective change in measurable tumor size by Resist criteria	Enrollment:<br>33<br><br>Age:<br>18 Years to 85 Years (Adult, Older Adult)<br><br>Sex:<br>All	• SignPath Pharma, Inc.	• Industry	Study Start:<br>March 2014<br><br>Primary Completion:<br>June 2017<br><br>Study Completion:<br>June 30, 2017<br><br>First Posted:<br>May 15, 2014<br><br>Results First Posted:<br>No Results Posted<br><br>Last Update Posted:<br>May 9, 2017	• 3 Medicizinische Universitatsklinik Hematologie,Internistische Onkologie, Salzburg, Austria<br>• 3. Medizinische Universitatesclinik, Haematologie, Internistische Onkologie, Salzburg, Austria

	NCT Number	Title	Other Names	Status	Conditions	Interventions	Characteristics	Population	Sponsor/ Collaborators	Funder Type	Dates	Locations
31	NCT02598726	Curcumin and Piperine in Reducing Inflammation for Ureteral Stent-Induced Symptoms in Patients With Cancer<br><br>Study Documents:	Title Acronym:<br><br>Other Ids:<br>•MC1511<br>•NCI-2015-01716<br>•P30CA015083	Active, not recruiting	•Bladder Spasm<br>•Malignant Neoplasm<br>•Pain<br>•Urinary Urgency	•Drug: Curcumin<br>•Other: Laboratory Biomarker Analysis<br>•Dietary Supplement: Piperine Extract (Standardized)<br>•Other: Quality-of-Life Assessment	Study Type: Interventional<br><br>Phase: Phase 1<br><br>Study Design:<br>•Allocation: N/A<br>•Intervention Model: Single Group Assignment<br>•Masking: None (Open Label)<br>•Primary Purpose: Supportive Care<br><br>Outcome Measures:<br>•Incidence of adverse events, using the National Cancer Institute Common Terminology Criteria for Adverse Events version 4.0<br>•Maximum tolerated dose (MTD) of curcumin in combination with piperine<br>•Optimal biologically active dose for curcumin in combination with piperine extract (standardized)<br>•Change in quality of life (QOL) by means of the USSQ	Enrollment: 9<br><br>Age: 18 Years and older (Adult, Older Adult)<br><br>Sex: All	•Mayo Clinic<br>•National Cancer Institute (NCI)	•Other<br>•NIH	Study Start: March 1, 2016<br><br>Primary Completion: November 6, 2019<br><br>Study Completion: July 15, 2022<br><br>First Posted: November 6, 2015<br><br>Results First Posted: No Results Posted<br><br>Last Update Posted: April 12, 2022	•Mayo Clinic, Rochester, Minnesota, United States
32	NCT04266275	Topical Curcumin for HPV Related Cervical Disease<br><br>Study Documents:	Title Acronym:<br><br>Other Ids:<br>•STUDY00000758<br>•IRB00117251	Not yet recruiting	•Neoplasm Cervix	•Drug: Curcumin C3 Complex<br>•Drug: Placebo	Study Type: Interventional<br><br>Phase: Phase 2<br><br>Study Design:<br>•Allocation: Randomized<br>•Intervention Model: Parallel Assignment<br>•Masking: Triple (Participant, Care Provider, Investigator)<br>•Primary Purpose: Treatment<br><br>Outcome Measures:<br>•HPV Clearance<br>•Disease Recurrence	Enrollment: 200<br><br>Age: 21 Years and older (Adult, Older Adult)<br><br>Sex: Female	•Lisa Flowers<br>•Emory University	•Other	Study Start: September 2022<br><br>Primary Completion: December 2023<br><br>Study Completion: December 2024<br><br>First Posted: February 12, 2020<br><br>Results First Posted: No Results Posted<br><br>Last Update Posted: May 2, 2022	•Emory University, Atlanta, Georgia, United States<br>•Grady Memorial Hospital, Atlanta, Georgia, United States<br>•Grady Hospital - Ponce De Leon Clinic, Atlanta, Georgia, United States

	NCT Number	Title	Other Names	Status	Conditions	Interventions	Characteristics	Population	Sponsor/ Collaborators	Funder Type	Dates	Locations
33	NCT02095717	Multicenter Study Comparing Taxotere Plus Curcumin Versus Taxotere Plus Placebo Combination in First-line Treatment of Prostate Cancer Metastatic Castration Resistant (CURTAXEL)<br><br>Study Documents:	Title Acronym: CURTAXEL<br><br>Other Ids: 2013-002138-20	Terminated	• Prostate Cancer Metastatic Castration Resistant	• Drug: Curcumin<br>• Drug: Placebo<br>• Drug: Taxotere	Study Type: Interventional<br><br>Phase: Phase 2<br><br>Study Design:<br>• Allocation: Randomized<br>• Intervention Model: Parallel Assignment<br>• Masking: Triple (Participant, Care Provider, Investigator)<br>• Primary Purpose: Treatment<br><br>Outcome Measures:<br>• Time to progression<br>• PSA response<br>• objective tumor response rate<br>• safety and tolerability<br>• Pain<br>• neuroendocrine markers<br>• Overall survival<br>• anti-angiogenic activity<br>• compliance	Enrollment: 50<br><br>Age: 18 Years and older (Adult, Older Adult)<br><br>Sex: Male	• Centre Jean Perrin	• Other	Study Start: March 2014<br><br>Primary Completion: April 2018<br><br>Study Completion: April 2018<br><br>First Posted: March 26, 2014<br><br>Results First Posted: No Results Posted<br><br>Last Update Posted: July 24, 2018	• Clinique de la Chataigneraie, Beaumont, France<br>• Centre Jean Perrin, Clermont-Ferrand, France<br>• Centre Hospitalier Emile Roux, Le Puy en velay, France<br>• Institut Jean Godinot, Reims, France

	NCT Number	Title	Other Names	Status	Conditions	Interventions	Characteristics	Population	Sponsor/ Collaborators	Funder Type	Dates	Locations
34	NCT01975363	Pilot Study of Curcumin for Women With Obesity and High Risk for Breast Cancer<br><br>Study Documents:	Title Acronym:<br><br>Other Ids:<br>• OSU-13034<br>• NCI-2013-01199	Completed	• Atypical Ductal Breast Hyperplasia<br>• BRCA1 Gene Mutation<br>• BRCA2 Gene Mutation<br>• Ductal Breast Carcinoma in Situ<br>• Lobular Breast Carcinoma in Situ	• Dietary Supplement: curcumin<br>• Other: Biomarker analysis<br>• Other: Assessment of Dietary Intake<br>• Other: Daily Log	Study Type: Interventional<br><br>Phase: Not Applicable<br><br>Study Design:<br>• Allocation: Randomized<br>• Intervention Model: Parallel Assignment<br>• Masking: None (Open Label)<br>• Primary Purpose: Prevention<br><br>Outcome Measures:<br>• Determine the adherence, tolerability and safety of two doses of nanoemulsion curcumin (NEC)in women at high risk for developing breast cancer.<br>• Evaluate possible correlations between physical factors such as body mass (BMI), dietary intake and pro-inflammatory effects in plasma and breast adipose tissue.	Enrollment: 29<br><br>Age: 19 Years and older (Adult, Older Adult)<br><br>Sex: Female	• Ohio State University Comprehensive Cancer Center	• Other	Study Start: June 2013<br><br>Primary Completion: September 19, 2016<br><br>Study Completion: September 19, 2016<br><br>First Posted: November 3, 2013<br><br>Results First Posted: No Results Posted<br><br>Last Update Posted: August 30, 2019	• Arthur G. James Cancer Hospital and Solove Research Institute at Ohio State University Medical Center, Columbus, Ohio, United States

	NCT Number	Title	Other Names	Status	Conditions	Interventions	Characteristics	Population	Sponsor/ Collaborators	Funder Type	Dates	Locations
35	NCT04403568	Testing the Synergism of Phytonutrients, Curcumin and Ursolic Acid, to Target Molecular Pathways in the Prostate<br><br>Study Documents:	Title Acronym:<br><br>Other Ids: HSC20190735H	Withdrawn	• Prostate Cancer	• Drug: Ursolic Acid<br>• Drug: Curcumin	Study Type: Interventional<br><br>Phase: Early Phase 1<br><br>Study Design:<br>• Allocation: Non-Randomized<br>• Intervention Model: Sequential Assignment<br>• Masking: None (Open Label)<br>• Primary Purpose: Basic Science<br><br>Outcome Measures:<br>• Number, frequency, duration, and relation of toxicity events<br>• Peak serum concentration<br>• Time to reach peak serum concentration<br>• Half-life<br>• Levels of UA, CURC, and metabolites in prostate tissue<br>• Immunohistochemistry measurement	Enrollment: 0<br><br>Age: 18 Years and older (Adult, Older Adult)<br><br>Sex: Male	• The University of Texas Health Science Center at San Antonio	• Other	Study Start: October 2021<br><br>Primary Completion: December 2022<br><br>Study Completion: December 2023<br><br>First Posted: May 27, 2020<br><br>Results First Posted: No Results Posted<br><br>Last Update Posted: September 16, 2021	• Mays Cancer Center, San Antonio, Texas, United States

	NCT Number	Title	Other Names	Status	Conditions	Interventions	Characteristics	Population	Sponsor/ Collaborators	Funder Type	Dates	Locations
36	NCT00852332	Docetaxel With or Without a Phytochemical in Treating Patients With Breast Cancer<br>Study Documents:	Title Acronym:<br>Other Ids:<br>• CDR0000635901<br>• JEANP-CURRYTAX<br>• INCA-RECF0908<br>• EUDRACT-2008-0[illegible]	Terminated	• Breast Cancer	• Dietary Supplement: Curcumin<br>• Drug: Taxotere	Study Type: Interventional<br>Phase: Phase 2<br>Study Design:<br>• Allocation: Randomized<br>• Intervention Model: Parallel Assignment<br>• Masking: None (Open Label)<br>• Primary Purpose: Treatment<br>Outcome Measures:<br>• Response rate as assessed by RECIST criteria<br>• Overall clinical benefit rate as assessed by RECIST criteria<br>• Time to progression as assessed by RECIST criteria<br>• Overall survival as assessed by RECIST criteria<br>• Safety as assessed by NCI CTCAE v3.0	Enrollment: 42<br>Age: 18 Years to 120 Years (Adult, Older Adult)<br>Sex: All	• Centre Jean Perrin	• Other	Study Start: August 2009<br>Primary Completion: November 2017<br>Study Completion: November 2017<br>First Posted: February 27, 2009<br>Results First Posted: No Results Posted<br>Last Update Posted: July 24, 2018	• Centre Jean Perrin, Clermont-Ferrand, France
37	NCT01201694	Phase I Study of Surface-Controlled Water Soluble Curcumin (THERACURMIN CR-011L)<br>Study Documents:	Title Acronym:<br>Other Ids: 2010-0276	Completed	• Advanced Cancers	• Drug: Surface-Controlled Water Soluble Curcumin<br>• Drug: Surface-Controlled Water Soluble Curcumin MTD	Study Type: Interventional<br>Phase: Phase 1<br>Study Design:<br>• Allocation: Non-Randomized<br>• Intervention Model: Single Group Assignment<br>• Masking: None (Open Label)<br>• Primary Purpose: Treatment<br>Outcome Measures:<br>Maximum Tolerated Dose (MTD) of Surface-Controlled Water Dispersible Curcumin	Enrollment: 28<br>Age: 13 Years and older (Child, Adult, Older Adult)<br>Sex: All	• M.D. Anderson Cancer Center<br>• Gateway for Cancer Research<br>• Theravalues, Inc.	• Other<br>• Industry	Study Start: October 2011<br>Primary Completion: January 2014<br>Study Completion: January 2014<br>First Posted: September 14, 2010<br>Results First Posted: No Results Posted<br>Last Update Posted: January 8, 2015	• UT MD Anderson Cancer Center, Houston, Texas, United States

	NCT Number	Title	Other Names	Status	Conditions	Interventions	Characteristics	Population	Sponsor/ Collaborators	Funder Type	Dates	Locations
38	NCT00486460	Phase III Trial of Gemcitabine, Curcumin and Celebrex in Patients With Advance or Inoperable Pancreatic Cancer<br>Study Documents:	Title Acronym:<br>Other Ids: TASMC-07-NA-132-CTIL	Unknown status	•Pancreatic Cancer	•Drug: Gemcitabine<br>•Drug: Curcumin<br>•Drug: Celebrex	Study Type: Interventional<br>Phase: Phase 3<br>Study Design:<br>•Allocation: Randomized<br>•Intervention Model: Parallel Assignment<br>•Masking: Double<br>Outcome Measures:	Enrollment:<br>Age: 18 Years and older (Adult, Older Adult)<br>Sex: All	•Tel-Aviv Sourasky Medical Center	•Other	Study Start: June 2005<br>Primary Completion:<br>Study Completion:<br>First Posted: June 14, 2007<br>Results First Posted: No Results Posted<br>Last Update Posted: June 14, 2007	•Sourasky Medical Center, Tel Aviv, Israel
39	NCT01608139	Study of Curcumin, Vorinostat, and Sorafenib<br>Study Documents:	Title Acronym:<br>Other Ids: 2009-0574	Withdrawn	•Advanced Cancers	•Drug: Curcumin<br>•Drug: Sorafenib<br>•Drug: Vorinostat	Study Type: Interventional<br>Phase: Phase 1<br>Study Design:<br>•Allocation: N/A<br>•Intervention Model: Single Group Assignment<br>•Masking: None (Open Label)<br>•Primary Purpose: Treatment<br>Outcome Measures: Maximum Tolerated Dose (MTD)	Enrollment: 0<br>Age: 18 Years and older (Adult, Older Adult)<br>Sex: All	•M.D. Anderson Cancer Center<br>•Sabinsa Corporation	•Other<br>•Industry	Study Start: November 2012<br>Primary Completion: November 2022<br>Study Completion:<br>First Posted: May 30, 2012<br>Results First Posted: No Results Posted<br>Last Update Posted: October 19, 2012	

	NCT Number	Title	Other Names	Status	Conditions	Interventions	Characteristics	Population	Sponsor/ Collaborators	Funder Type	Dates	Locations
40	NCT00745134	Radiation Therapy and Capecitabine With or Without Curcumin Before Surgery in Treating Patients With Rectal Cancer<br><br>Study Documents:	Title Acronym:<br><br>Other Ids:<br>• 2006-0644<br>• NCI-2012-01676	Active, not recruiting	• Rectal Mucinous Adenocarcinoma<br>• Rectal Signet Ring Cell Adenocarcinoma<br>• Recurrent Rectal Carcinoma<br>• Stage IIA Rectal Cancer AJCC v7<br>• Stage IIB Rectal Cancer AJCC v7<br>• Stage IIC Rectal Cancer AJCC v7<br>• Stage IIIA Rectal Cancer AJCC v7<br>• Stage IIIB Rectal Cancer AJCC v7<br>• Stage IIIC Rectal Cancer AJCC v7	• Drug: Capecitabine<br>• Dietary Supplement: Curcumin<br>• Other: Laboratory Biomarker Analysis<br>• Other: Pharmacological Study<br>• Other: Placebo<br>• Other: Quality-of-Life Assessment<br>• Radiation: Radiation Therapy	Study Type:<br>Interventional<br><br>Phase:<br>Phase 2<br><br>Study Design:<br>• Allocation: Randomized<br>• Intervention Model: Parallel Assignment<br>• Masking: Double (Participant, Investigator)<br>• Primary Purpose: Treatment<br><br>Outcome Measures:<br>• Pathologic complete response (pCR) rate<br>• Change in curcumin level in tumor tissue<br>• Change in curcumin level in serum<br>• Change in quality of life (QoL) as assessed by MD Anderson Symptom Inventory-Gastrointestinal (MDASI-GI)<br>• Change in quality of life (QoL) as assessed by Brief Fatigue Inventory (BFI)	Enrollment:<br>45<br><br>Age:<br>18 Years and older (Adult, Older Adult)<br><br>Sex:<br>All	• M.D. Anderson Cancer Center<br>• National Cancer Institute (NCI)	• Other<br>• NIH	Study Start:<br>August 11, 2008<br><br>Primary Completion:<br>March 31, 2023<br><br>Study Completion:<br>March 31, 2023<br><br>First Posted:<br>September 3, 2008<br><br>Results First Posted:<br>No Results Posted<br><br>Last Update Posted:<br>April 27, 2022	• M D Anderson Cancer Center, Houston, Texas, United States

	NCT Number	Title	Other Names	Status	Conditions	Interventions	Characteristics	Population	Sponsor/ Collaborators	Funder Type	Dates	Locations
41	NCT02944578	Topical Curcumin for Precancer Cervical Lesions<br><br>Study Documents:	Title Acronym:<br><br>Other Ids:<br>IRB00079183	Suspended	•Neoplasms	•Drug: Curcumin<br>•Drug: Placebo	Study Type:<br>Interventional<br><br>Phase:<br>Phase 2<br><br>Study Design:<br>•Allocation: Randomized<br>•Intervention Model: Parallel Assignment<br>•Masking: Single (Outcomes Assessor)<br>•Primary Purpose: Treatment<br><br>Outcome Measures:<br>•Change in human papillomavirus (HPV) related molecular target HPV E6/E7 messenger ribonucleic acid (mRNA) expression within HSIL lesions of the cervix<br>•Quantify curcumin levels in cervical tissue	Enrollment:<br>40<br><br>Age:<br>21 Years and older (Adult, Older Adult)<br><br>Sex:<br>Female	•Emory University	•Other	Study Start:<br>November 20, 2017<br><br>Primary Completion:<br>October 2022<br><br>Study Completion:<br>October 2022<br><br>First Posted:<br>October 26, 2016<br><br>Results First Posted:<br>No Results Posted<br><br>Last Update Posted:<br>December 14, 2021	•Grady Memorial Hospital, Atlanta, Georgia, United States<br>•Grady Hospital - Ponce De Leon Clinic, Atlanta, Georgia, United States
42	NCT01048983	Reducing Symptom Burden - Non Small Cell Lung Cancer (NSCLC)<br><br>Study Documents:	Title Acronym:<br><br>Other Ids:<br>•2008-0345<br>•R01 026582-26<br>•NCI-2012-01256	Withdrawn	•Non Small Cell Lung Cancer	•Drug: Armodafinil<br>•Drug: Bupropion<br>•Drug: Minocycline<br>•Drug: Curcumin<br>•Behavioral: Telephone Questionnaire	Study Type:<br>Interventional<br><br>Phase:<br>•Phase 1<br>•Phase 2<br><br>Study Design:<br>•Allocation: Randomized<br>•Intervention Model: Single Group Assignment<br>•Masking: Triple (Participant, Care Provider, Investigator)<br>•Primary Purpose: Supportive Care<br><br>Outcome Measures:<br>Combined AUC for Selected Symptoms	Enrollment:<br>0<br><br>Age:<br>18 Years to 65 Years (Adult, Older Adult)<br><br>Sex:<br>All	•M.D. Anderson Cancer Center<br>•National Cancer Institute (NCI)	•Other<br>•NIH	Study Start:<br><br>Primary Completion:<br><br>Study Completion:<br><br>First Posted:<br>January 14, 2010<br><br>Results First Posted:<br>No Results Posted<br><br>Last Update Posted:<br>March 5, 2015	

	NCT Number	Title	Other Names	Status	Conditions	Interventions	Characteristics	Population	Sponsor/ Collaborators	Funder Type	Dates	Locations
43	NCT02556632	Prophylactic Topical Agents in Reducing Radiation-Induced Dermatitis in Patients With Non-inflammatory Breast Cancer<br><br>Study Documents:	Title Acronym: Curcumin-II<br><br>Other Ids:<br>• URCC14079<br>• NCI-2015-00869<br>• URCC-14079<br>• R21CA178648<br>• UG1CA189961	Completed	• Breast Carcinoma<br>• Pain<br>• Radiation-Induced Dermatitis<br>• Stage 0 Breast Cancer	• Drug: Curcumin-based Gel<br>• Procedure: Dermatologic Complications Management<br>• Other: Laboratory Biomarker Analysis<br>• Other: Placebo<br>• Other: Questionnaire Administration	Study Type: Interventional<br><br>Phase: Phase 2<br><br>Study Design:<br>• Allocation: Randomized<br>• Intervention Model: Parallel Assignment<br>• Masking: Triple (Participant, Investigator, Outcomes Assessor)<br>• Primary Purpose: Supportive Care<br><br>Outcome Measures:<br>• Mean Radiation Dermatitis Severity (RDS) Score. Range: 0 (no Dermatitis) - 4 (Violaceous Erythema With Diffuse Desquamation Occurring in Sheets; Patchy Crusting; Superficial Ulceration)<br>• Incidence of Moist Desquamation (Present vs. Absent)<br>• Change in the Severity of Skin Reactions Using the Radiation Dermatitis Scale (RDS). Range: 0 (no Dermatitis) - 4 (Violaceous Erythema With Diffuse Desquamation Occurring in Sheets; Patchy Crusting; Superficial Ulceration)	Enrollment: 191<br><br>Age: 21 Years and older (Adult, Older Adult)<br><br>Sex: Female	• Gary Morrow<br>• National Cancer Institute (NCI)<br>• University of Rochester	• Other<br>• NIH	Study Start: October 13, 2015<br><br>Primary Completion: September 30, 2016<br><br>Study Completion: September 30, 2016<br><br>First Posted: September 22, 2015<br><br>Results First Posted: November 6, 2017<br><br>Last Update Posted: November 6, 2017	• Delaware/Christiana Care NCORP, Newark, Delaware, United States<br>• Heartland NCORP, Decatur, Illinois, United States<br>• Metro-Minnesota NCORP, Minneapolis, Minnesota, United States<br>• University of Rochester, Rochester, New York, United States<br>• Columbus NCORP, Columbus, Ohio, United States<br>• Dayton Oncology Research Program, Dayton, Ohio, United States

	NCT Number	Title	Other Names	Status	Conditions	Interventions	Characteristics	Population	Sponsor/ Collaborators	Funder Type	Dates	Locations
44	NCT03192059	Study of Pembrolizumab, Radiation and Immune Modulatory Cocktail in Cervical/ Uterine Cancer<br><br>Study Documents:	Title Acronym: PRIMMO<br><br>Other Ids: 2016-001569-97	Completed	•Cervical Cancer<br>•Endometrial Cancer<br>•Uterine Cancer	•Drug: Pembrolizumab<br>•Radiation: Radiation<br>•Drug: Vitamin D<br>•Drug: Aspirin<br>•Drug: Lansoprazole<br>•Drug: Cyclophosphamide<br>•Dietary Supplement: Curcumin	Study Type: Interventional<br><br>Phase: Phase 2<br><br>Study Design:<br>•Allocation: N/A<br>•Intervention Model: Single Group Assignment<br>•Masking: None (Open Label)<br>•Primary Purpose: Treatment<br><br>Outcome Measures:<br>•Objective response rate at week 26<br>•Incidence of treatment-emergent adverse events (Safety according to CTCAE4.0).<br>•Objective response rate<br>•Best OR<br>•PFS<br>•Median PFS<br>•OS<br>•Median OS<br>•Quality of life assessment	Enrollment: 43<br><br>Age: 18 Years and older (Adult, Older Adult)<br><br>Sex: Female	•University Hospital, Ghent<br>•Kom Op Tegen Kanker<br>•Anticancer Fund, Belgium	•Other	Study Start: July 1, 2017<br><br>Primary Completion: June 30, 2021<br><br>Study Completion: June 30, 2021<br><br>First Posted: June 19, 2017<br><br>Results First Posted: No Results Posted<br><br>Last Update Posted: September 8, 2021	•University Hospital Antwerp, Antwerp, Belgium<br>•Institut Jules Bordet, Brussels, Belgium<br>•University Hospital Gent, Gent, Belgium<br>•CMSE Namur, Namur, Belgium
45	NCT00003365	Sulindac and Plant Compounds in Preventing Colon Cancer<br><br>Study Documents:	Title Acronym:<br><br>Other Ids:<br>•CDR0000066350<br>•P30CA016056<br>•RUH-SSH-190-0600<br>•RUH-SSH-190-0698<br>•NCI-V98-1425	Terminated	•Colorectal Cancer	•Dietary Supplement: curcumin<br>•Dietary Supplement: rutin<br>•Drug: quercetin<br>•Drug: sulindac	Study Type: Interventional<br><br>Phase: Not Applicable<br><br>Study Design:<br>•Allocation: Randomized<br>•Primary Purpose: Prevention<br><br>Outcome Measures:	Enrollment:<br><br>Age: 18 Years and older (Adult, Older Adult)<br><br>Sex: All	•University of Medicine and Dentistry of New Jersey<br>•National Cancer Institute (NCI)<br>•Rutgers, The State University of New Jersey	•Other<br>•NIH	Study Start: August 1996<br><br>Primary Completion: July 2006<br><br>Study Completion: July 2006<br><br>First Posted: May 21, 2004<br><br>Results First Posted: No Results Posted<br><br>Last Update Posted: January 27, 2011	•Rockefeller University Hospital, New York, New York, United States

	NCT Number	Title	Other Names	Status	Conditions	Interventions	Characteristics	Population	Sponsor/ Collaborators	Funder Type	Dates	Locations
46	NCT02017353	Effect of Curcumin Addition to Standard Treatment on Tumour-induced Inflammation in Endometrial Carcinoma<br>Study Documents:	Title Acronym:<br>Other Ids:<br>• S55201<br>• 2013-001737-40	Completed	• Endometrial Carcinoma	• Dietary Supplement: Curcuphyt	Study Type: Interventional<br>Phase: Phase 2<br>Study Design:<br>• Allocation: N/A<br>• Intervention Model: Single Group Assignment<br>• Masking: None (Open Label)<br>• Primary Purpose: Treatment<br>Outcome Measures:<br>• Change in inflammatory markers in peripheral blood from baseline<br>• Number of Participants with Serious and Non-Serious Adverse Events<br>• Change from Baseline in Quality of Life score	Enrollment: 7<br>Age: 18 Years and older (Adult, Older Adult)<br>Sex: Female	• University Hospital, Gasthuisberg<br>• Reliable Cancer Therapies	• Other<br>• Industry	Study Start: October 2013<br>Primary Completion: May 2016<br>Study Completion: October 2016<br>First Posted: December 20, 2013<br>Results First Posted: No Results Posted<br>Last Update Posted: October 25, 2016	• University Hospital KU Leuven Campus Gasthuisberg, Leuven, Belgium
47	NCT01246973	Oral Curcumin for Radiation Dermatitis<br>Study Documents:	Title Acronym:<br>Other Ids:<br>• URCC 10054<br>• URCC 09005<br>• U10CA037420	Completed	• Radiation-induced Dermatitis	• Drug: Curcumin<br>• Drug: Placebo	Study Type: Interventional<br>Phase:<br>• Phase 2<br>• Phase 3<br>Study Design:<br>• Allocation: Randomized<br>• Intervention Model: Parallel Assignment<br>• Masking: Triple (Participant, Care Provider, Investigator)<br>• Primary Purpose: Prevention<br>Outcome Measures:<br>• Mean Radiation Dermatitis Severity Score<br>• Percentage of Subjects With Moist Desquamation	Enrollment: 686<br>Age: 21 Years to 120 Years (Adult, Older Adult)<br>Sex: Female	• University of Rochester<br>• National Cancer Institute (NCI)	• Other<br>• NIH	Study Start: February 2011<br>Primary Completion: November 2014<br>Study Completion: January 2015<br>First Posted: November 24, 2010<br>Results First Posted: March 7, 2016<br>Last Update Posted: March 7, 2016	

	NCT Number	Title	Other Names	Status	Conditions	Interventions	Characteristics	Population	Sponsor/ Collaborators	Funder Type	Dates	Locations
48	NCT03598309	Phase II Trial to Modulate Intermediate Endpoint Biomarkers in Former and Current Smokers<br><br>Study Documents:	Title Acronym:<br><br>Other Ids:<br>•MCC-19622<br>•8JK03	Recruiting	•Lung Diseases<br>•Lung Cancer, Protection Against	•Drug: Curcumin C3 complex®<br>•Drug: Lovaza®<br>•Other: Placebo	Study Type: Interventional<br><br>Phase: Phase 2<br><br>Study Design:<br>•Allocation: Randomized<br>•Intervention Model: Parallel Assignment<br>•Masking: Triple (Participant, Care Provider, Investigator)<br>•Primary Purpose: Prevention<br><br>Outcome Measures:<br>•Mean Change in Bronchial Nodule Size<br>•Rate of Nodules #4 mm<br>•Rate of Adherence<br>•Rate of Treatment Related Adverse Events (AEs)	Enrollment: 75<br><br>Age: 55 Years and older (Adult, Older Adult)<br><br>Sex: All	•H. Lee Moffitt Cancer Center and Research Institute<br>•James and Esther King Biomedical Research Program	•Other	Study Start: June 5, 2019<br><br>Primary Completion: October 2022<br><br>Study Completion: October 2023<br><br>First Posted: July 26, 2018<br><br>Results First Posted: No Results Posted<br><br>Last Update Posted: April 27, 2022	•H. Lee Moffitt Cancer Center and Research Institute, Tampa, Florida, United States
49	NCT02782949	Curcumin in Preventing Gastric Cancer in Patients With Chronic Atrophic Gastritis or Gastric Intestinal Metaplasia<br><br>Study Documents:	Title Acronym:<br><br>Other Ids:<br>•NCI-2016-00713<br>•N01-CN-2012-00042<br>•MAY2015-05-01<br>•N01CN00042<br>•P30CA015083	Active, not recruiting	•Chronic Atrophic Gastritis	•Drug: Curcumin<br>•Other: Laboratory Biomarker Analysis<br>•Other: Placebo Administration<br>•Other: Quality-of-Life Assessment	Study Type: Interventional<br><br>Phase: Phase 2<br><br>Study Design:<br>•Allocation: Randomized<br>•Intervention Model: Parallel Assignment<br>•Masking: Double (Participant, Investigator)<br>•Primary Purpose: Prevention<br><br>Outcome Measures:<br>•Absolute change in IL-1beta cytokine levels in the gastric mucosa<br>•Incidence of adverse events<br>•Change in histology gastric score<br>•Additional gastric mucosal cytokine/chemokine levels (IL-8, TNFalpha, and IP-10)<br>•Gastric mucosal deoxyribonucleic acid (DNA) damage	Enrollment: 100<br><br>Age: 21 Years and older (Adult, Older Adult)<br><br>Sex: All	•National Cancer Institute (NCI)	•NIH	Study Start: April 4, 2017<br><br>Primary Completion: May 31, 2022<br><br>Study Completion: May 31, 2022<br><br>First Posted: May 26, 2016<br><br>Results First Posted: No Results Posted<br><br>Last Update Posted: March 18, 2022	•Hospital Regional de Occidente, Santa Rosa De Copan, Honduras<br>•University of Puerto Rico, San Juan, Puerto Rico

	NCT Number	Title	Other Names	Status	Conditions	Interventions	Characteristics	Population	Sponsor/ Collaborators	Funder Type	Dates	Locations
50	NCT00118989	Curcumin for the Chemoprevention of Colorectal Cancer<br>Study Documents:	Title Acronym:<br>Other Ids: 802193	Terminated	• Adenomatous Polyps	• Dietary Supplement: Curcuminoids	Study Type: Interventional<br>Phase: Not Applicable<br>Study Design:<br>• Allocation: Randomized<br>• Intervention Model: Parallel Assignment<br>• Masking: Double (Participant, Investigator)<br>• Primary Purpose: Prevention<br>Outcome Measures:<br>• Cellular proliferation and apoptosis in the colonic mucosa<br>• COX-2 expression and activity	Enrollment: 56<br>Age: 18 Years and older (Adult, Older Adult)<br>Sex: All	• University of Pennsylvania<br>• Robert Wood Johnson Foundation	• Other	Study Start: July 2005<br>Primary Completion: September 2010<br>Study Completion: July 2012<br>First Posted: July 12, 2005<br>Results First Posted: No Results Posted<br>Last Update Posted: April 20, 2017	• University of Pennsylvania, Philadelphia, Pennsylvania, United States
51	NCT03493997	Multicentre International STudy for the Prevention With Ialuril® of Radio-induced Cystitis (MISTIC)<br>Study Documents:	Title Acronym: MISTIC<br>Other Ids: MISTIC	Completed	• Prostate Cancer	• Combination Product: Radiotherapy +IAluril®+Ialuril Soft Gels®<br>• Radiation: Radiotherapy only	Study Type: Interventional<br>Phase: Phase 2<br>Study Design:<br>• Allocation: Randomized<br>• Intervention Model: Parallel Assignment<br>• Masking: None (Open Label)<br>• Primary Purpose: Prevention<br>Outcome Measures:<br>• Rate of patients<br>• comparative analysis of score between the two groups through ICIQ<br>• comparative analysis of score between the two groups through EPIC<br>• comparative analysis of score of QOL<br>• comparative analysis of score between the two groups through IPSS	Enrollment: 100<br>Age: 18 Years and older (Adult, Older Adult)<br>Sex: Male	• Study Group for Urogenital Diseases, Italy	• Other	Study Start: April 4, 2017<br>Primary Completion: October 31, 2018<br>Study Completion: May 31, 2020<br>First Posted: April 11, 2018<br>Results First Posted: No Results Posted<br>Last Update Posted: July 23, 2020	• Ospedali Careggi, Firenze, Italy<br>• Università "Vita-Salute" San Raffaele, Milano, Italy<br>• University of Palermo, Italy, Palermo, Italy<br>• St. Cyril and Method University Hospital, Bratislava, Slovakia<br>• Fakultná nemocnica s poliklinikou, Prešov, Slovakia<br>• Universidad Autónoma de Barcelona, Barcelona, Spain<br>• Istanbul University, Istanbul, Turkey

	NCT Number	Title	Other Names	Status	Conditions	Interventions	Characteristics	Population	Sponsor/ Collaborators	Funder Type	Dates	Locations
52	NCT02336087	Gemcitabine Hydrochloride, Paclitaxel Albumin-Stabilized Nanoparticle Formulation, Metformin Hydrochloride, and a Standardized Dietary Supplement in Treating Patients With Pancreatic Cancer That Cannot be Removed by Surgery<br><br>Study Documents:	Title Acronym:<br><br>Other Ids:<br>• 14122<br>• NCI-2014-02612	Active, not recruiting	• Pancreatic Adenocarcinoma<br>• Unresectable Pancreatic Carcinoma<br>• Stage III Pancreatic Cancer AJCC v6 and v7<br>• Stage IV Pancreatic Cancer AJCC v6 and v7	• Drug: Gemcitabine Hydrochloride<br>• Drug: Paclitaxel Albumin-Stabilized Nanoparticle Formulation<br>• Drug: Metformin Hydrochloride<br>• Dietary Supplement: Therapeutic Dietary Intervention<br>• Other: Laboratory Biomarker Analysis<br>• Other: Quality-of-Life Assessment	Study Type:<br>Interventional<br><br>Phase:<br>Phase 1<br><br>Study Design:<br>• Allocation: N/A<br>• Intervention Model: Single Group Assignment<br>• Masking: None (Open Label)<br>• Primary Purpose: Treatment<br><br>Outcome Measures:<br>• Feasibility of the combination of gemcitabine hydrochloride, paclitaxel albumin-stabilized nanoparticle formulation, metformin hydrochloride, and a dietary supplement<br>• Compliance of the combination of gemcitabine hydrochloride, paclitaxel albumin-stabilized nanoparticle formulation, metformin hydrochloride, and a dietary supplement (percent of patients who are fully compliant)<br>• Toxicity of the combination of gemcitabine hydrochloride, paclitaxel albumin-stabilized nanoparticle formulation, metformin hydrochloride, and a dietary supplement (National Cancer Institute Common Terminology for Adverse Events criteria version 4)<br>• Progression-free survival<br>• Overall survival<br>• Time to treatment failure	Enrollment:<br>21<br><br>Age:<br>18 Years and older (Adult, Older Adult)<br><br>Sex:<br>All	• City of Hope Medical Center<br>• National Cancer Institute (NCI)	• Other<br>• NIH	Study Start:<br>January 14, 2016<br><br>Primary Completion:<br>October 4, 2020<br><br>Study Completion:<br>December 31, 2022<br><br>First Posted:<br>January 12, 2015<br><br>Results First Posted:<br>No Results Posted<br><br>Last Update Posted:<br>March 18, 2022	• City of Hope Medical Center, Duarte, California, United States<br>• City of Hope Rancho Cucamonga, Rancho Cucamonga, California, United States<br>• City of Hope South Pasadena, South Pasadena, California, United States<br>• City of Hope West Covina, West Covina, California, United States

	NCT Number	Title	Other Names	Status	Conditions	Interventions	Characteristics	Population	Sponsor/ Collaborators	Funder Type	Dates	Locations
53	NCT01269203	Efficacy of NF-kB Inhibition for Reducing Symptoms During Maintenance Therapy in Multiple Myeloma Patients<br><br>Study Documents:	Title Acronym:<br><br>Other Ids:<br>2010-0457	Withdrawn	•Multiple Myeloma	•Drug: Curcumin<br>•Other: Placebo	Study Type:<br>Interventional<br><br>Phase:<br>Phase 2<br><br>Study Design:<br>•Allocation: Randomized<br>•Intervention Model: Single Group Assignment<br>•Masking: Triple (Participant, Care Provider, Investigator)<br>•Primary Purpose: Treatment<br><br>Outcome Measures:<br>AUC from 3 months Post-Transplantation to 9 months Post-Transplantation	Enrollment:<br>0<br><br>Age:<br>18 Years and older (Adult, Older Adult)<br><br>Sex:<br>All	•M.D. Anderson Cancer Center<br>•National Institutes of Health (NIH)	•Other<br>•NIH	Study Start:<br>October 2012<br><br>Primary Completion:<br>October 2015<br><br>Study Completion:<br><br>First Posted:<br>January 4, 2011<br><br>Results First Posted:<br>No Results Posted<br><br>Last Update Posted:<br>September 7, 2012	

	NCT Number	Title	Other Names	Status	Conditions	Interventions	Characteristics	Population	Sponsor/ Collaborators	Funder Type	Dates	Locations
54	NCT00641147	Curcumin in Treating Patients With Familial Adenomatous Polyposis<br><br>Study Documents:	Title Acronym:<br><br>Other Ids:<br>• NCI-2013-00536<br>• CDR0000592794<br>• NA_00011821<br>• 1R01CA134620<br>• R01CA134620<br>• P30CA006973	Completed	• Familial Adenomatous Polyposis	• Drug: Curcumin<br>• Other: Laboratory Biomarker Analysis<br>• Other: Placebo	Study Type:<br>Interventional<br><br>Phase:<br>Phase 2<br><br>Study Design:<br>• Allocation: Randomized<br>• Intervention Model: Parallel Assignment<br>• Masking: Double (Participant, Investigator)<br>• Primary Purpose: Treatment<br><br>Outcome Measures:<br>• Polyp Number<br>• Mean Polyp Size in mm<br>• Number of Participants With a Decrease in Polyp Burden at 12 Months<br>• Number of Participants With Grade >=2 Adverse Events<br>• Medication Compliance<br>• Change in Ornithine Decarboxylase (ODC) Activity Levels<br>• Change in Total Polyamines Levels<br>• Change in Micro RNA 124-U6 (miR124-U6)<br>• Change in Spermidine/ Spermine N-1 Acetyl Transferase (SSAT)<br>• Change in Spermine Oxidase (SMOX)<br>• Change in Ki-67 Anti-proliferative Cell Nuclear Antibody Index Levels<br>• Change in Apoptosis Index Levels	Enrollment:<br>44<br><br>Age:<br>18 Years to 85 Years (Adult, Older Adult)<br><br>Sex:<br>All	• National Cancer Institute (NCI)	• NIH	Study Start:<br>October 2010<br><br>Primary Completion:<br>November 2016<br><br>Study Completion:<br>November 30, 2016<br><br>First Posted:<br>March 24, 2008<br><br>Results First Posted:<br>September 29, 2017<br><br>Last Update Posted:<br>September 29, 2017	• Johns Hopkins University/ Sidney Kimmel Cancer Center, Baltimore, Maryland, United States<br>• University of Puerto Rico, San Juan, Puerto Rico

	NCT Number	Title	Other Names	Status	Conditions	Interventions	Characteristics	Population	Sponsor/ Collaborators	Funder Type	Dates	Locations
55	NCT00248053	Use of Curcumin in the Lower Gastrointestinal Tract in Familial Adenomatous Polyposis Patients<br>Study Documents:	Title Acronym:<br>Other Ids: 05-04-12-04	Withdrawn	• Familial Adenomatous Polyposis	• Drug: curcumin	Study Type: Interventional<br>Phase: Phase 2<br>Study Design: • Allocation: Non-Randomized • Intervention Model: Crossover Assignment • Masking: None (Open Label)<br>Outcome Measures: • polyp number and size • side effects and medication compliance	Enrollment: 0<br>Age: 18 Years to 85 Years (Adult, Older Adult)<br>Sex: All	• Johns Hopkins University	• Other	Study Start: November 2005<br>Primary Completion: June 2008<br>Study Completion:<br>First Posted: November 3, 2005<br>Results First Posted: No Results Posted<br>Last Update Posted: September 20, 2012	
56	NCT01035580	Trial on Safety and Pharmacokinetics of Intravaginal Curcumin<br>Study Documents:	Title Acronym:<br>Other Ids: IRB00020139	Completed	• Uterine Cervical Dysplasia	• Drug: Curcumin<br>• Drug: curcumin	Study Type: Interventional<br>Phase: Phase 1<br>Study Design: • Allocation: N/A • Intervention Model: Single Group Assignment • Masking: None (Open Label) • Primary Purpose: Treatment<br>Outcome Measures: The primary endpoint is to reach the maximum selected dose (MSD) or maximum tolerated dose (MTD) of intravaginal curcumin among Pap test negative women without causing a dose-limiting toxicity.	Enrollment: 13<br>Age: 18 Years to 45 Years (Adult)<br>Sex: Female	• Emory University	• Other	Study Start: January 2010<br>Primary Completion: April 2011<br>Study Completion: January 2012<br>First Posted: December 18, 2009<br>Results First Posted: No Results Posted<br>Last Update Posted: November 28, 2013	• Emory University, Atlanta, Georgia, United States

	NCT Number	Title	Other Names	Status	Conditions	Interventions	Characteristics	Population	Sponsor/ Collaborators	Funder Type	Dates	Locations
57	NCT00113841	Curcumin (Diferuloylmethane Derivative) With or Without Bioperine in Patients With Multiple Myeloma<br>Study Documents:	Title Acronym:<br>Other Ids: 2003-0436	Completed	• Multiple Myeloma	• Drug: Curcumin<br>• Drug: Bioperine	Study Type: Interventional<br>Phase: Not Applicable<br>Study Design:<br>• Allocation: Randomized<br>• Intervention Model: Parallel Assignment<br>• Masking: None (Open Label)<br>• Primary Purpose: Treatment<br>Outcome Measures: Percent Change of NF-kB Protein Expression in Peripheral Blood Mononuclear Cells From Baseline Through 4 Weeks of Treatment	Enrollment: 42<br>Age: Child, Adult, Older Adult<br>Sex: All	• M.D. Anderson Cancer Center	• Other	Study Start: November 2004<br>Primary Completion: August 2009<br>Study Completion: August 2009<br>First Posted: June 13, 2005<br>Results First Posted: October 19, 2011<br>Last Update Posted: November 23, 2011	• U.T.M.D. Anderson Cancer Center, Houston, Texas, United States
58	NCT03482401	Disposition of Dietary Polyphenols and Methylxanthines in Mammary Tissues From Breast Cancer Patients<br>Study Documents:	Title Acronym: POLYSEN<br>Other Ids: 201770E081	Completed	• Breast Cancer	• Dietary Supplement: Polyphenol	Study Type: Interventional<br>Phase: Not Applicable<br>Study Design:<br>• Allocation: Randomized<br>• Intervention Model: Parallel Assignment<br>• Masking: None (Open Label)<br>• Primary Purpose: Screening<br>Outcome Measures:<br>• Quantification of dietary polyphenols and methylxanthines in breast tissues<br>• Quantification of dietary polyphenols and methylxanthines in plasma and urine	Enrollment: 40<br>Age: 18 Years and older (Adult, Older Adult)<br>Sex: All	• National Research Council, Spain<br>• Hospital General Universitario Reina Sofía de Murcia	• Other	Study Start: June 1, 2017<br>Primary Completion: December 1, 2018<br>Study Completion: December 31, 2019<br>First Posted: March 29, 2018<br>Results First Posted: No Results Posted<br>Last Update Posted: September 10, 2020	• Hospital General Universitario Reina Sofía, Murcia, Spain

	NCT Number	Title	Other Names	Status	Conditions	Interventions	Characteristics	Population	Sponsor/ Collaborators	Funder Type	Dates	Locations
59	NCT00927485	Use of Curcumin for Treatment of Intestinal Adenomas in Familial Adenomatous Polyposis (FAP)<br><br>Study Documents:	Title Acronym:<br><br>Other Ids:<br>Protocol A2210108-UPR	Completed	•Familial Adenomatous Polyposis	•Drug: Calcumin (Curcumin)<br>•Other: Risk Factor Questionnaire<br>•Other: Blood samples<br>•Other: Biopsies (Sigmoidoscopy)<br>•Other: Biopsies (Upper endoscopy)	Study Type:<br>Interventional<br><br>Phase:<br>Not Applicable<br><br>Study Design:<br>•Allocation: Randomized<br>•Intervention Model: Parallel Assignment<br>•Masking: Double (Participant, Investigator)<br>•Primary Purpose: Treatment<br><br>Outcome Measures:<br>•Number of Polyps<br>•Size of Polyps	Enrollment:<br>44<br><br>Age:<br>21 Years to 85 Years (Adult, Older Adult)<br><br>Sex:<br>All	•University of Puerto Rico<br>•National Institutes of Health (NIH)	•Other<br>•NIH	Study Start:<br>November 2007<br><br>Primary Completion:<br>November 2016<br><br>Study Completion:<br>December 2016<br><br>First Posted:<br>June 25, 2009<br><br>Results First Posted:<br>December 16, 2020<br><br>Last Update Posted:<br>December 16, 2020	•University of Puerto Rico Comprehensive Cancer Center, San Juan, Puerto Rico

	NCT Number	Title	Other Names	Status	Conditions	Interventions	Characteristics	Population	Sponsor/ Collaborators	Funder Type	Dates	Locations
60	NCT04871412	The Thoracic Peri-Operative Integrative Surgical Care Evaluation Trial - Stage II<br><br>Study Documents:	Title Acronym: POISE<br><br>Other Ids: 20200796-01H	Recruiting	• Lung Cancer<br>• Gastric Cancer<br>• Esophageal Cancer	• Dietary Supplement: Vitamin D3 Drops<br>• Dietary Supplement: Coriolus Versicolor<br>• Dietary Supplement: Trident SAP 66:33 Lemon<br>• Dietary Supplement: Probiotic Pro12<br>• Dietary Supplement: Provitalix Pure Whey Protein<br>• Dietary Supplement: Theracurmin 2X<br>• Dietary Supplement: Green Tea Extract<br>• Other: Nutrition Recommendations<br>• Other: Physical Activity Recommendations<br>• Behavioral: Psychological Recommendations	Study Type: Interventional<br><br>Phase: Phase 3<br><br>Study Design:<br>• Allocation: Randomized<br>• Intervention Model: Parallel Assignment<br>• Masking: None (Open Label)<br>• Primary Purpose: Treatment<br><br>Outcome Measures:<br>• Participant Recruitment Rates<br>• Participant Retention Rates<br>• Cross-over and contamination in the control arm - Supplement usage<br>• Cross-over and contamination in the control arm - Mediterranean Diet Scores<br>• Cross-over and contamination in the control arm - Physical Activity levels<br>• Cross-over and contamination in the control arm - Psychological Health Activities<br>• Communication<br>• Natural Killer Cell Function<br>• Qualitative Experience<br>• Inflammatory Response<br>• Neutrophil to Lymphocyte Ratio	Enrollment: 20<br><br>Age: 18 Years and older (Adult, Older Adult)<br><br>Sex: All	• Ottawa Hospital Research Institute<br>• The Canadian College of Naturopathic Medicine<br>• Lotte & John Hecht Memorial Foundation<br>• The Centre for Health Innovation<br>• University of Ottawa	• Other	Study Start: April 4, 2022<br><br>Primary Completion: May 1, 2024<br><br>Study Completion: May 1, 2025<br><br>First Posted: May 4, 2021<br><br>Results First Posted: No Results Posted<br><br>Last Update Posted: April 15, 2022	• The Ottawa Hospital, Ottawa, Ontario, Canada

	NCT Number	Title	Other Names	Status	Conditions	Interventions	Characteristics	Population	Sponsor/ Collaborators	Funder Type	Dates	Locations
61	NCT05306002	Nutritional Intervention and DNA Damage of Patients With HBOC<br><br>Study Documents:	Title Acronym:<br><br>Other Ids:<br>ONCONOV001	Active, not recruiting	•HBOC Syndrome<br>•DNA Damage<br>•Nutrition Therapy	•Combination Product: Antioxidant therapy	Study Type:<br>Interventional<br><br>Phase:<br>Not Applicable<br><br>Study Design:<br>•Allocation: N/A<br>•Intervention Model: Single Group Assignment<br>•Masking: None (Open Label)<br>•Primary Purpose: Treatment<br><br>Outcome Measures:<br>•DNA damage change<br>•Body composition change<br>•Muscular strength change<br>•Dietary change: Energy<br>•Dietary change: Macronutrients<br>•Dietary change: Micronutrients	Enrollment:<br>34<br><br>Age:<br>18 Years and older (Adult, Older Adult)<br><br>Sex:<br>Female	•Instituto de Seguridad y Servicios Sociales de los Trabajadores del Estado	•Other	Study Start:<br>November 28, 2017<br><br>Primary Completion:<br>March 22, 2020<br><br>Study Completion:<br>December 31, 2022<br><br>First Posted:<br>March 31, 2022<br><br>Results First Posted:<br>No Results Posted<br><br>Last Update Posted:<br>March 31, 2022	•María Fernanda Díaz Yáñez, Mexico City, Benito Juárez, Mexico

	NCT Number	Title	Other Names	Status	Conditions	Interventions	Characteristics	Population	Sponsor/ Collaborators	Funder Type	Dates	Locations
62	NCT03061591	Turmeric Supplementation on Polyp Number and Size in Patients With Familial Adenomatous Polyposis.<br><br>Study Documents:	Title Acronym:<br><br>Other Ids: TASMC-15-RK-0601-CTIL	Unknown status	•Familial Adenomatous Polyposis<br>•FAP<br>•FAP Gene Mutation	•Dietary Supplement: Wholistic Turmeric capsules<br>•Other: Placebo	Study Type: Interventional<br><br>Phase: Phase 2<br><br>Study Design:<br>•Allocation: Randomized<br>•Intervention Model: Parallel Assignment<br>•Masking: Quadruple (Participant, Care Provider, Investigator, Outcomes Assessor)<br>•Primary Purpose: Treatment<br><br>Outcome Measures:<br>•Number of polyps<br>•Size of polyps<br>•Histological apoptosis assays in frozen polyps and normal tissue (such as KI67, caspase 3 activity and COX-2 expression).<br>•Histological proliferation assays in frozen polyps and normal tissue (such as KI67, caspase 3 activity and COX-2 expression).<br>•Colonic microbiome composition, after curcumin therapy.<br>•Duodenal adenoma number.<br>•Duodenal adenoma size.<br>•Curcumin and curcuminoid levels in blood.<br>•Curcumin and curcuminoid levels in urine.	Enrollment: 40<br><br>Age: 18 Years to 70 Years (Adult, Older Adult)<br><br>Sex: All	•Tel-Aviv Sourasky Medical Center	•Other	Study Start: April 2017<br><br>Primary Completion: April 2018<br><br>Study Completion: April 2020<br><br>First Posted: February 23, 2017<br><br>Results First Posted: No Results Posted<br><br>Last Update Posted: February 23, 2017	

	NCT Number	Title	Other Names	Status	Conditions	Interventions	Characteristics	Population	Sponsor/ Collaborators	Funder Type	Dates	Locations
63	NCT01948661	Anthocyanin Extract and Phospholipid Curcumin in Colorectal Adenoma<br><br>Study Documents:	Title Acronym: MIRACOL<br><br>Other Ids: GAL 02	Unknown status	• Colorectal Adenoma<br>• Risk Reduction	• Dietary Supplement: Mirtoselect® + Meriva®<br>• Dietary Supplement: Placebo	Study Type: Interventional<br><br>Phase: Not Applicable<br><br>Study Design:<br>• Allocation: Randomized<br>• Intervention Model: Parallel Assignment<br>• Masking: Quadruple (Participant, Care Provider, Investigator, Outcomes Assessor)<br>• Primary Purpose: Prevention<br><br>Outcome Measures:<br>• Change of immunohistochemical expression of beta catenin in normal and adenomatous colonic tissue<br>• Change of IHC Nuclear Factor-K# (NFK#), Ki-67 Labeling Index and P53 in normal and adenomatous mucosa.	Enrollment: 100<br><br>Age: 18 Years to 75 Years (Adult, Older Adult)<br><br>Sex: All	• Ente Ospedaliero Ospedali Galliera<br>• Fondazione Umberto Veronesi<br>• Indena S.p.A	• Other<br>• Industry	Study Start: March 2014<br><br>Primary Completion: September 2019<br><br>Study Completion: December 2019<br><br>First Posted: September 23, 2013<br><br>Results First Posted: No Results Posted<br><br>Last Update Posted: August 1, 2018	• ASL 3, Ospedale Villa Scassi, S.C. Gastroenterologia, Genova, Italy<br>• Medical Oncology Ente Ospedaliero Ospedali Galliera, Genova, Italy<br>• ASL4 Chiavarese, Ospedale di Lavagna, SSD Gastroenterologia, Lavagna, Italy

	NCT Number	Title	Other Names	Status	Conditions	Interventions	Characteristics	Population	Sponsor/ Collaborators	Funder Type	Dates	Locations
64	NCT00176618	The Effects of Curcuminoids on Aberrant Crypt Foci in the Human Colon<br><br>Study Documents:	Title Acronym:<br><br>Other Ids:<br>•4611<br>•CINJ#120305	Terminated	•Aberrant Crypt Foci	•Drug: sulindac<br>•Drug: curcumin	Study Type:<br>Interventional<br><br>Phase:<br>Not Applicable<br><br>Study Design:<br>•Allocation: Randomized<br>•Intervention Model: Single Group Assignment<br>•Masking: None (Open Label)<br>•Primary Purpose: Prevention<br><br>Outcome Measures:<br>•To evaluate the effects of curcumin or the NSAID sulindac on the number of ACF in the left colon and rectum of normal volunteers found to have them on an initial magnifying chromoendoscopic screening exam<br>•To determine the turnover (proliferation, apoptosis, and differentiation) of colorectal epithelial cells in the crypts in situ in response to each treatment	Enrollment:<br>60<br><br>Age:<br>18 Years and older (Adult, Older Adult)<br><br>Sex:<br>All	•University of Medicine and Dentistry of New Jersey<br>•Rutgers, The State University of New Jersey	•Other	Study Start:<br>April 2004<br><br>Primary Completion:<br>June 2007<br><br>Study Completion:<br>June 2007<br><br>First Posted:<br>September 15, 2005<br><br>Results First Posted:<br>No Results Posted<br><br>Last Update Posted:<br>December 11, 2009	•Cancer Institute of New Jersey, New Brunswick, New Jersey, United States

	NCT Number	Title	Other Names	Status	Conditions	Interventions	Characteristics	Population	Sponsor/ Collaborators	Funder Type	Dates	Locations
65	NCT02300727	Study to See How Safe Curcumin is and How Well it Works When Used to Treat Mucositis in Patients Getting Chemotherapy<br><br>Study Documents:<br>• Study Protocol and Statistical Analysis Plan	Title Acronym:<br><br>Other Ids:<br>Curcumin:I/II	Terminated	• Mucositis	• Drug: Curcumin-MTD<br>• Drug: Mouthwash-standard pharmacy preparation<br>• Drug: Curcumin	Study Type:<br>Interventional<br><br>Phase:<br>• Phase 1<br>• Phase 2<br><br>Study Design:<br>• Allocation: Randomized<br>• Intervention Model: Parallel Assignment<br>• Masking: None (Open Label)<br>• Primary Purpose: Treatment<br><br>Outcome Measures:<br>• Number of Participants With Serious and Non-Serious Adverse Events<br>• Change in Toxicities Graded by Health Care Providers Using the Common Terminology Criteria for Adverse Events (CTCAE) Version 4.0<br>• Change in Subjective Patient Self-assessment of Pain.<br>• Change in Subjective Patient Self- Assessment of Oral Mucositis Measured by the Common Terminology Criteria for Adverse Events (CTCAE) and World Health Organization's (WHO's) Oral Toxicity Scale (OTS)<br>• Change in Health Providers Assessment of Oral Mucositis and Healing Time Measured by the Common Terminology Criteria for Adverse Events (CTCAE) and World Health Organization's (WHO's) Oral Toxicity Scale (OTS)	Enrollment:<br>6<br><br>Age:<br>18 Years and older (Adult, Older Adult)<br><br>Sex:<br>All	• Amy Beres<br>• Aurora BayCare Medical Center	• Other	Study Start:<br>February 2015<br><br>Primary Completion:<br>October 25, 2018<br><br>Study Completion:<br>October 25, 2018<br><br>First Posted:<br>November 25, 2014<br><br>Results First Posted:<br>May 31, 2019<br><br>Last Update Posted:<br>May 31, 2019	• Aurora BayCare Medical Center, Green Bay, Wisconsin, United States

	NCT Number	Title	Other Names	Status	Conditions	Interventions	Characteristics	Population	Sponsor/ Collaborators	Funder Type	Dates	Locations
66	NCT00689195	Pilot Study of Curcumin Formulation and Ashwagandha Extract in Advanced Osteosarcoma<br>Study Documents:	Title Acronym: OSCAT<br>Other Ids: 381	Unknown status	• Osteosarcoma	• Dietary Supplement: Curcumin powder<br>• Dietary Supplement: Ashwagandha extract	Study Type: Interventional<br>Phase: • Phase 1 • Phase 2<br>Study Design: • Allocation: Non-Randomized • Intervention Model: Parallel Assignment • Masking: None (Open Label) • Primary Purpose: Treatment<br>Outcome Measures: • response, toxicity, disease progression • quality of life	Enrollment: 24<br>Age: 8 Years to 65 Years (Child, Adult, Older Adult)<br>Sex: All	• Tata Memorial Hospital<br>• Pharmanza Herbals Pvt Limited (PHPL)	• Other	Study Start: May 2008<br>Primary Completion: June 2013<br>Study Completion: June 2013<br>First Posted: June 3, 2008<br>Results First Posted: No Results Posted<br>Last Update Posted: June 23, 2011	• Tata Memorial Hospital, Mumbai, Maharashtra, India
67	NCT05045443	Safety and Efficacy of Curcumin in Children With Acute Lymphoblastic Leukemia<br>Study Documents:	Title Acronym: CurcumPedALL<br>Other Ids: FMASU MD 134/2021	Recruiting	• Acute Lymphoblastic Leukemia, Pediatric	• Drug: Curcumin<br>• Dietary Supplement: Standard of Care	Study Type: Interventional<br>Phase: Phase 2<br>Study Design: • Allocation: Randomized • Intervention Model: Parallel Assignment • Masking: None (Open Label) • Primary Purpose: Supportive Care<br>Outcome Measures: Determine the Safety of curcumin in pediatric patients with ALL.	Enrollment: 60<br>Age: 1 Year to 18 Years (Child, Adult)<br>Sex: All	• Ain Shams University	• Other	Study Start: August 22, 2021<br>Primary Completion: June 2022<br>Study Completion: September 2022<br>First Posted: September 16, 2021<br>Results First Posted: No Results Posted<br>Last Update Posted: September 17, 2021	• Pediatric Hematology Oncology and BMT Unit, Faculty of Medicine Ain Shams University, Cairo, Non-US, Egypt

	NCT Number	Title	Other Names	Status	Conditions	Interventions	Characteristics	Population	Sponsor/ Collaborators	Funder Type	Dates	Locations
68	NCT00365209	Phase II A Trial of Curcumin Among Patients With Prevalent Subclinical Neoplastic Lesions (Aberrant Crypt Foci)<br>Study Documents:	Title Acronym:<br>Other Ids:<br>•NCI-2013-00449<br>•UIC-2005-0617<br>•CDR0000483003<br>•UCIRVINE-2005-45[illegible]<br>•UIC HS# 2005-0617<br>•CCUM-HUM00000731<br>•P30CA062203<br>•2005-0617<br>•UCI04-2-01<br>•N01CN35160	Completed	•Healthy, no Evidence of Disease<br>•Tobacco Use Disorder	•Other: laboratory biomarker analysis<br>•Other: pharmacological study<br>•Drug: curcumin	Study Type:<br>Interventional<br>Phase:<br>Phase 2<br>Study Design:<br>•Allocation: Non-Randomized<br>•Intervention Model: Single Group Assignment<br>•Masking: None (Open Label)<br>•Primary Purpose: Prevention<br>Outcome Measures:<br>•Baseline in Prostaglandin E2 (PGE2) Within Aberrant Crypt Foci (ACF)<br>•Post-treatment in Prostaglandin E2 (PGE2) Within Aberrant Crypt Foci (ACF)<br>•Baseline in 5-hydroxy-eicosatetraenoic Acid (5-HETE) Within Aberrant Crypt Foci (ACF)<br>•Post-treatment in 5-hydroxy-eicosatetraenoic Acid (5-HETE) Within Aberrant Crypt Foci (ACF)<br>•Baseline in Prostaglandin E2 (PGE2) Level in Normal Mucosa<br>•Post-treatment in Prostaglandin E2 (PGE2) Level in Normal Mucosa<br>•Baseline in 5-hydroxy-eicosatetraenoic Acid (5-HETE) Level in Normal Mucosa<br>•Post-treatment in 5-hydroxy-eicosatetraenoic Acid (5-HETE) Level in Normal Mucosa<br>•Change in Cyclooxygenases (COX-1, COX-2), and Lipoxygenase (5-LOX) Protein Abundance<br>•Changes in Total Aberrant Crypt Foci (ACF) Number<br>•and 12 more	Enrollment:<br>44<br>Age:<br>40 Years and older (Adult, Older Adult)<br>Sex:<br>All	•National Cancer Institute (NCI)	•NIH	Study Start:<br>October 2006<br>Primary Completion:<br>September 2008<br>Study Completion:<br>January 2011<br>First Posted:<br>August 17, 2006<br>Results First Posted:<br>August 27, 2015<br>Last Update Posted:<br>August 27, 2015	•Chao Family Comprehensive Cancer Center, Orange, California, United States

	NCT Number	Title	Other Names	Status	Conditions	Interventions	Characteristics	Population	Sponsor/ Collaborators	Funder Type	Dates	Locations
69	NCT01712542	Curcumin Bioavailability in Glioblastoma Patients<br><br>Study Documents:	Title Acronym:<br><br>Other Ids:<br>JohannWGUH_Curc	Completed	• Patient Harboring Glioblastoma That Will Undergo Surgery		Study Type:<br>Observational<br><br>Phase:<br><br>Study Design:<br>• Observational Model: Case-Only<br>• Time Perspective: Prospective<br><br>Outcome Measures:<br>Concentration of Curcumin in Glioblastoma	Enrollment:<br>15<br><br>Age:<br>18 Years and older (Adult, Older Adult)<br><br>Sex:<br>All	• Johann Wolfgang Goethe University Hospital	• Other	Study Start:<br>October 2012<br><br>Primary Completion:<br>May 2013<br><br>Study Completion:<br>May 2013<br><br>First Posted:<br>October 23, 2012<br><br>Results First Posted:<br>No Results Posted<br><br>Last Update Posted:<br>June 4, 2013	• Department of Neurosurgery, Johann Wolfgang Goethe-University, Frankfurt, Hessen, Germany
70	NCT02100423	Curcumin and Cholecalciferol in Treating Patients With Previously Untreated Stage 0-II Chronic Lymphocytic Leukemia or Small Lymphocytic Lymphoma<br><br>Study Documents:	Title Acronym:<br><br>Other Ids:<br>• CASE5913<br>• NCI-2014-00266<br>• P30CA043703	Completed	• Contiguous Stage II Small Lymphocytic Lymphoma<br>• Noncontiguous Stage II Small Lymphocytic Lymphoma<br>• Stage 0 Chronic Lymphocytic Leukemia<br>• Stage I Chronic Lymphocytic Leukemia<br>• Stage I Small Lymphocytic Lymphoma<br>• Stage II Chronic Lymphocytic Leukemia	• Drug: curcumin<br>• Dietary Supplement: cholecalciferol<br>• Other: laboratory biomarker analysis<br>• Other: pharmacological study	Study Type:<br>Interventional<br><br>Phase:<br>Phase 2<br><br>Study Design:<br>• Allocation: N/A<br>• Intervention Model: Single Group Assignment<br>• Masking: None (Open Label)<br>• Primary Purpose: Treatment<br><br>Outcome Measures:<br>• Overall response rate (biologic response rate + complete response [CR] + partial response [PR]) based on NCI-WG (for CLL) and Cheson criteria (for SLL)<br>• Time to first cytotoxic treatment<br>• Progression free survival<br>• Overall survival<br>• Duration of response	Enrollment:<br>35<br><br>Age:<br>18 Years and older (Adult, Older Adult)<br><br>Sex:<br>All	• Paolo Caimi, MD<br>• National Cancer Institute (NCI)<br>• Case Comprehensive Cancer Center	• Other<br>• NIH	Study Start:<br>September 26, 2014<br><br>Primary Completion:<br>April 4, 2018<br><br>Study Completion:<br>December 13, 2018<br><br>First Posted:<br>April 1, 2014<br><br>Results First Posted:<br>No Results Posted<br><br>Last Update Posted:<br>January 18, 2020	• Case Comprehensive Cancer Center, Cleveland, Ohio, United States

	NCT Number	Title	Other Names	Status	Conditions	Interventions	Characteristics	Population	Sponsor/ Collaborators	Funder Type	Dates	Locations
71	NCT00969085	Trial of Curcumin in Cutaneous T-cell Lymphoma Patients<br><br>Study Documents:	Title Acronym:<br><br>Other Ids:<br>2007-0838	Withdrawn	•Cutaneous T-cell Lymphoma	•Drug: Curcumin (Turmeric)<br>•Behavioral: Questionnaires<br>•Other: Photos	Study Type:<br>Interventional<br><br>Phase:<br>Phase 2<br><br>Study Design:<br>•Allocation: N/A<br>•Intervention Model: Single Group Assignment<br>•Masking: None (Open Label)<br>•Primary Purpose: Treatment<br><br>Outcome Measures:<br>Response Rate using Physician's Global Assessment (PGA) based on Severity-Weighted Assessment Tool (SWAT)	Enrollment:<br>0<br><br>Age:<br>Child, Adult, Older Adult<br><br>Sex:<br>All	•M.D. Anderson Cancer Center	•Other	Study Start:<br>November 2012<br><br>Primary Completion:<br>November 2014<br><br>Study Completion:<br><br>First Posted:<br>August 31, 2009<br><br>Results First Posted:<br>No Results Posted<br><br>Last Update Posted:<br>September 7, 2012	
72	NCT00247026	The Efficacy of Coenzyme Q10 And Curcumin in Patients With Myelodysplastic Syndromes<br><br>Study Documents:	Title Acronym:<br><br>Other Ids:<br>•385-mds 1-HMO-CTIL<br>•mds 1	Withdrawn	•Myelodysplastic Syndrome	•Drug: curcumin; coenzyme q10	Study Type:<br>Interventional<br><br>Phase:<br>•Phase 1<br>•Phase 2<br><br>Study Design:<br>•Allocation: Non-Randomized<br>•Intervention Model: Single Group Assignment<br>•Masking: None (Open Label)<br>•Primary Purpose: Treatment<br><br>Outcome Measures:<br>•major hematologic improvement in any lineage<br>•Time to disease progression<br>•Overall and progression-free survival<br>•Cytogenetic response	Enrollment:<br>50<br><br>Age:<br>16 Years and older (Child, Adult, Older Adult)<br><br>Sex:<br>All	•Hadassah Medical Organization	•Other	Study Start:<br>April 2007<br><br>Primary Completion:<br><br>Study Completion:<br><br>First Posted:<br>November 1, 2005<br><br>Results First Posted:<br>No Results Posted<br><br>Last Update Posted:<br>April 11, 2007	•Hadassah Medical Organization, Jerusalem, Israel

	NCT Number	Title	Other Names	Status	Conditions	Interventions	Characteristics	Population	Sponsor/ Collaborators	Funder Type	Dates	Locations
73	NCT03431896	Monitoring of Early Disease Progression in Hereditary Transthyretin Amyloidosis<br><br>Study Documents:	Title Acronym: MED-hATTR<br><br>Other Ids: 17-1301	Recruiting	• Amyloidosis<br>• Amyloid<br>• Amyloid Neuropathies, Familial<br>• Amyloid Cardiomyopathy<br>• Amyloid - Primary<br>• Transthyretin Amyloidosis<br>• AL Amyloidosis		Study Type: Observational<br><br>Phase:<br><br>Study Design:<br>• Observational Model: Cohort<br>• Time Perspective: Prospective<br><br>Outcome Measures:<br>• Average % change in oligomers in patients with new onset TTR amyloid symptoms<br>• % change of oligomer levels relative to baseline level in patients with ATTR specific medication changes	Enrollment: 30<br><br>Age: Child, Adult, Older Adult<br><br>Sex: All	• The Cleveland Clinic	• Other	Study Start: February 1, 2018<br><br>Primary Completion: February 15, 2024<br><br>Study Completion: February 15, 2024<br><br>First Posted: February 13, 2018<br><br>Results First Posted: No Results Posted<br><br>Last Update Posted: March 28, 2022	• Cleveland Clinic, Cleveland, Ohio, United States
