# Supplementary material for: Curcumin, thymoquinone, and 3, 3′-diindolylmethane combinations attenuate lung and liver cancers progression
Source: Front Pharmacol. 2022 Jun 29;13:936996. doi: 10.3389/fphar.2022.936996 (PMC9277483; doi:10.3389/fphar.2022.936996)
Supplement: Supplementary file 8 [file Image1.pdf]

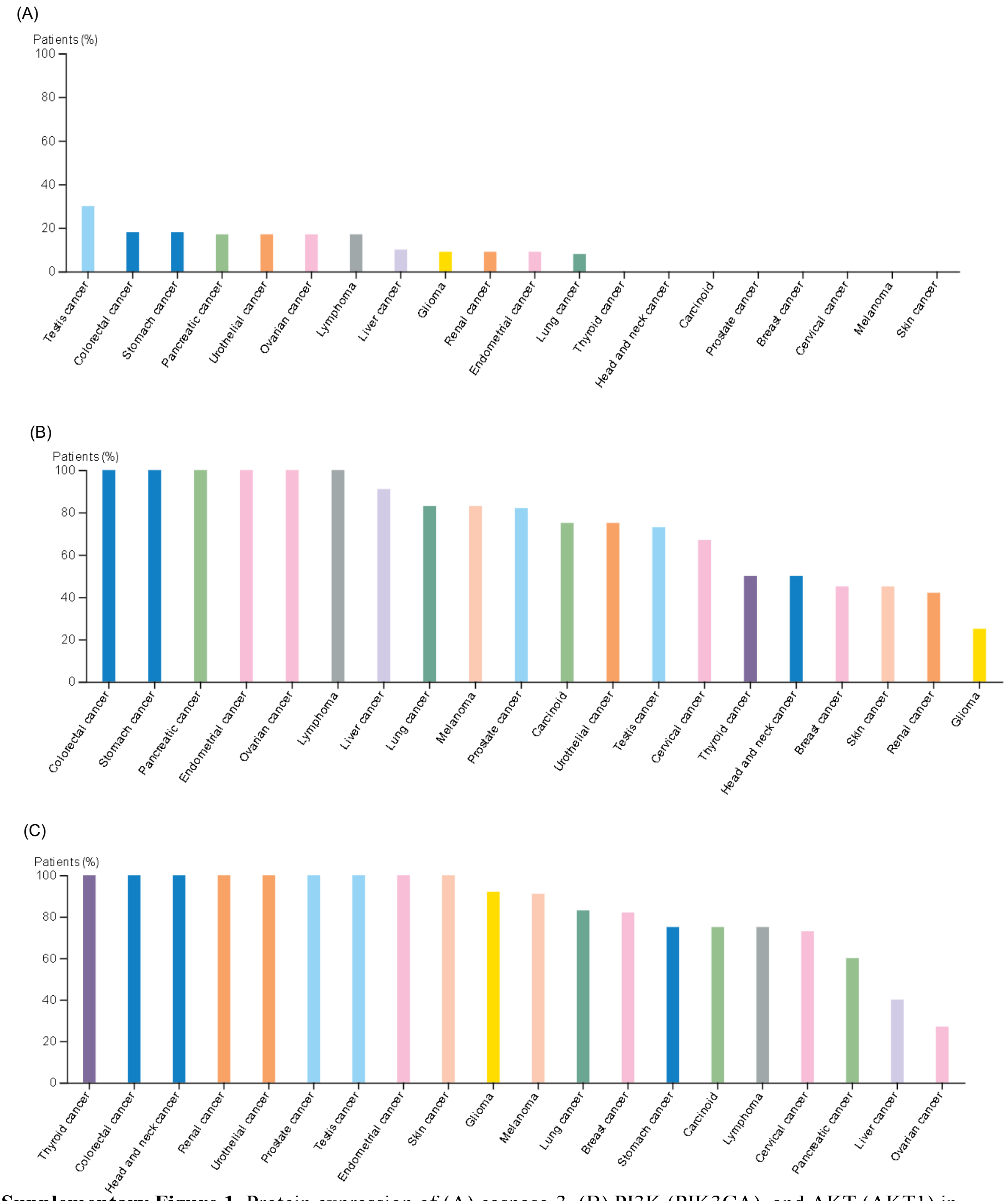

Supplementary Figure 1. Protein expression of (A) caspase-3, (B) PI3K (PIK3CA), and AKT (AKT1) in different cancer types searched by human protein atlas (HPA) (https://www.proteinatlas.org/).
